# Functionalization of Silica Nanoparticles for Tailored Interactions with Intestinal Cells and Chemical Modulation of Paracellular Permeability

**DOI:** 10.1002/smsc.202400112

**Published:** 2024-08-01

**Authors:** Claudia Iriarte‐Mesa, Janice Bergen, Kristina Danielyan, Francesco Crudo, Doris Marko, Hanspeter Kählig, Giorgia Del Favero, Freddy Kleitz

**Affiliations:** ^1^ Department of Functional Materials and Catalysis, Faculty of Chemistry University of Vienna Währinger Str. 42 1090 Vienna Austria; ^2^ Vienna Doctoral School in Chemistry (DoSChem) University of Vienna Währinger Str. 42 1090 Vienna Austria; ^3^ Core Facility Multimodal Imaging, Faculty of Chemistry University of Vienna Währinger Str. 38‐40 1090 Vienna Austria; ^4^ Department of Food Chemistry and Toxicology, Faculty of Chemistry University of Vienna Währinger Str. 38‐40 1090 Vienna Austria; ^5^ Department of Organic Chemistry, Faculty of Chemistry University of Vienna Währinger Str. 38 1090 Vienna Austria

**Keywords:** differentiated intestinal cells, membrane permeation, mesoporous silica nanoparticles, mucus barrier, surface functions, tight junction proteins

## Abstract

The intestinal compartment confines the gut microbiome while enabling food passage and absorption of active molecules. For the rational design of oral formulations aiming to overcome physiological barriers of the gut, it is crucial to understand how cells respond to the presence of nanoparticulate materials. Taking advantage of the versatility and biocompatibility of dendritic mesoporous silica nanoparticles (DMSNs), several post‐grafting strategies are developed to diversify the surface properties of spherical DMSNs and then probe interactions with the intestinal coculture cell model Caco‐2/HT29‐MTX‐E12. Herein, the functionalization of DMSNs with polyethylene glycol, phosphonate, methyl, and farnesol moieties enables the investigation of both particle penetration through the mucus layer and pathways relevant to intracellular uptake. Contributions of surface chemistry, charge, and colloidal stability are correlated with the modulation of particle movement through the mucus and the organization of cell–cell junctions. Hydrophilic and negative functionalities favor particle distribution toward the intestinal monolayer. Instead, hydrophobic DMSNs are hindered by the mucus, possibly limiting cell contact. Hybrid surfaces, combining phosphonate and long carbon chain functions, support diffusion through the mucus and foster the paracellular permeability as well as the transient barrier relapse, as indicated by increased cell–cell distances and reorganization of tight junctions.

## Graphical abstract

Herein, the crucial role of the surface chemistry of mesoporous silica nanoparticles in modulating intestinal barrier function is explored, considering the influence of the mucus and intracellular uptake pathways. Functionalization of spherical particles enables paracellular permeability through the cell monolayervia the re‐distribution of tight junction proteins and selective interactions with the cell membrane.

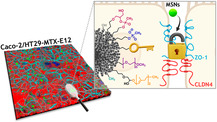

## 1 Introduction

In recent years, the oral delivery of peptide drugs and biologics has reached unprecedented attention.^[^ ^1^, ^2^, ^3^ ^]^ Despite its undeniable advantages, oral administration remains challenging due to the harsh conditions of the gastrointestinal tract and the presence of physiological barriers (e.g., digestive enzymes, mucus, and tight epithelium), which lead to poor drug bioavailability.^[^ ^1^, ^2^ ^]^ The intestinal compartment exhibits selective permeability that enables the absorption of essential nutrients while limiting the uptake of pathogens and contaminants.^[^ ^4^, ^5^ ^]^ These functions rely on a complex architecture that operates as a protective layer integrating both endogenous and exogenous stimuli (e.g., cytokines, bacteria, dietary factors, etc.).^[^ ^6^ ^]^ The regulation of barrier function is achieved by a unique interplay between structural components and molecular interactions that behave dynamically to maintain integrity and immune homeostasis in the gut.^[^ ^5^, ^6^ ^]^ Therefore, a deep understanding of how intestinal cells adjust in the presence of drug carriers is a crucial starting point for the design of optimized oral delivery formulations.^[^ ^7^, ^8^, ^9^ ^]^ At the same time, this can improve tolerability, and support pharmacological specificity and drug bioavailability.^[^ ^10^, ^11^, ^12^ ^]^

Mesoporous silica nanoparticles (MSNs) have demonstrated valuable potential in oral drug delivery applications.^[^ ^13^, ^14^, ^15^, ^16^ ^]^ Their high biocompatibility, limited toxicity, well‐defined porosity, and tunable surface chemistry have reinforced the rational design of MSNs for efficient interaction with intestinal cells and enhanced oral delivery of macromolecules.^[^ ^7^, ^12^ ^]^ It has been described that parameters such as size,^[^ ^17^, ^18^ ^]^ morphology,^[^ ^19^, ^20^, ^21^ ^]^ surface roughness,^[^ ^22^, ^23^ ^]^ and charge^[^ ^17^, ^24^ ^]^ affect the biodistribution, excretion, and toxicity of the nanoparticles, and also influence the differential interaction of MSNs with the cell membrane.^[^ ^11^, ^25^ ^]^ Special interest has also been placed on the surface chemistry of MSNs, which can likewise modulate colloidal stability,^[^ ^26^ ^]^ penetration through the mucus layer,^[^ ^27^, ^28^ ^]^ protein corona formation,^[^ ^29^, ^30^ ^]^ interaction with the epithelium,^[^ ^29^, ^31^ ^]^ cellular uptake,^[^ ^26^, ^32^, ^33^ ^]^ and intestinal permeation,^[^ ^19^, ^28^ ^]^ as well as retention and biodistribution of orally administered nanoparticles.^[^ ^34^, ^35^ ^]^

Various strategies for functionalization of MSNs have enabled the grafting of small molecules,^[^ ^32^, ^33^, ^36^, ^37^, ^38^ ^]^ liposomes,^[^ ^39^ ^]^ cell‐penetrating peptides,^[^ ^40^, ^41^ ^]^ as well as biocompatible polymers.^[^ ^18^, ^42^ ^]^ The biological performance of functionalized nanoparticles has been extensively studied. In this context, it has been described that neutral or negatively charged MSNs with hydrophilic grafted moieties can diffuse better through the mucus layer due to their limited interactions with the highly negative and hydrophobic proteoglycans called mucins.^[^ ^40^, ^43^, ^44^, ^45^ ^]^ Overall, the high viscosity and dense packing of the mucus, composed of water, globular proteins, salts, and lipids, in addition to mucins, limit transit of MSNs.^[^ ^46^ ^]^ Moreover, non‐polar hydrophobic nanoparticles exhibit enhanced contact with the lipid bilayer of the membrane.^[^ ^47^ ^]^ In contrast, charged and polar MSNs could either be adsorbed on the lipid headgroups or remain in the water layer.^[^ ^19^ ^]^ Positively charged nanoparticles have shown efficient interaction with mucus while being easily internalized by cells.^[^ ^32^, ^48^ ^]^ These complementary capacities have enabled the development of bioinert and biocompatible zwitterion‐functionalized MSNs in order to facilitate both mucus penetration and efficient absorption through the epithelial layer.^[^ ^49^ ^]^ Zwitterion‐functionalized materials have hydrophilic and neutral surfaces that are greatly beneficial for mucus permeation while resembling the cellular phospholipid membrane.^[^ ^49^, ^50^, ^51^ ^]^ Therefore, the anion and cation groups (e.g., phosphorylcholine, carboxybetaine, and sulfobetaine) of the zwitterion coatings have contributed to improved MSNs contact with the cell membrane and subsequent cellular uptake.^[^ ^49^ ^]^ On the other hand, some MSNs, especially those with spherical shape, can be adsorbed to external cell membranes rather than internalized.^[^ ^52^ ^]^ This may be associated with aggregation and lack of colloidal stability in biological media,^[^ ^19^, ^25^ ^]^ but also with selective interaction with intercellular tight junctions (TJs) rather than with receptors mediating cellular uptake.^[^ ^53^, ^54^ ^]^

TJs connect and hold cells together in the epithelium, forming a complex network of proteins (e.g., claudins, occludin, and zonula occludens) that extend across the space of adjacent cells and regulate paracellular permeability.^[^ ^54^ ^]^ The association of MSNs with TJs could disrupt these structures and increase epithelial permeability.^[^ ^55^, ^56^, ^57^ ^]^ Hence, the fundamental question arises whether chemically tuned interactions of MSNs with intestinal cells can selectively modulate intestinal permeability and lead to barrier function adjustment. Triggering physical and biochemical adaption could ultimately privilege nanoparticle transport either via intracellular or paracellular routes.^[^ ^48^, ^54^ ^]^ Although these mechanisms have been addressed from different perspectives,^[^ ^58^, ^59^, ^60^ ^]^ much remains to be understood about responses that can be achieved by surface chemistry modifications. An updated and in‐depth insight into tailored surface functionalization affecting the activity profile of MSNs, in close alignment with the integrity, architecture, and permeability of the intestinal barrier, could greatly support the rational design of nanocarriers, enabling differential interaction with the epithelium to boost new routes for systemic or local delivery of orally administered drugs.

Herein, DMSNs were used to explore the contribution of surface chemistry in the interaction between materials and intestinal cells. Spherical DMSNs have shown excellent potential as drug carriers in oral delivery,^[^ ^61^ ^]^ exhibiting a unique open central–radial structure with large pore channels and high biocompatibility,^[^ ^62^ ^]^ in addition to the ease of controlling size and porosity based on the synthesis conditions.^[^ ^62^, ^63^ ^]^ Motivated by this, functionalized DMSNs were generated to include functional groups with different polarities and systematically explore the interactions with the mucus layer and the cell surface.

## 2 Results and Discussion

**Scheme 1:**
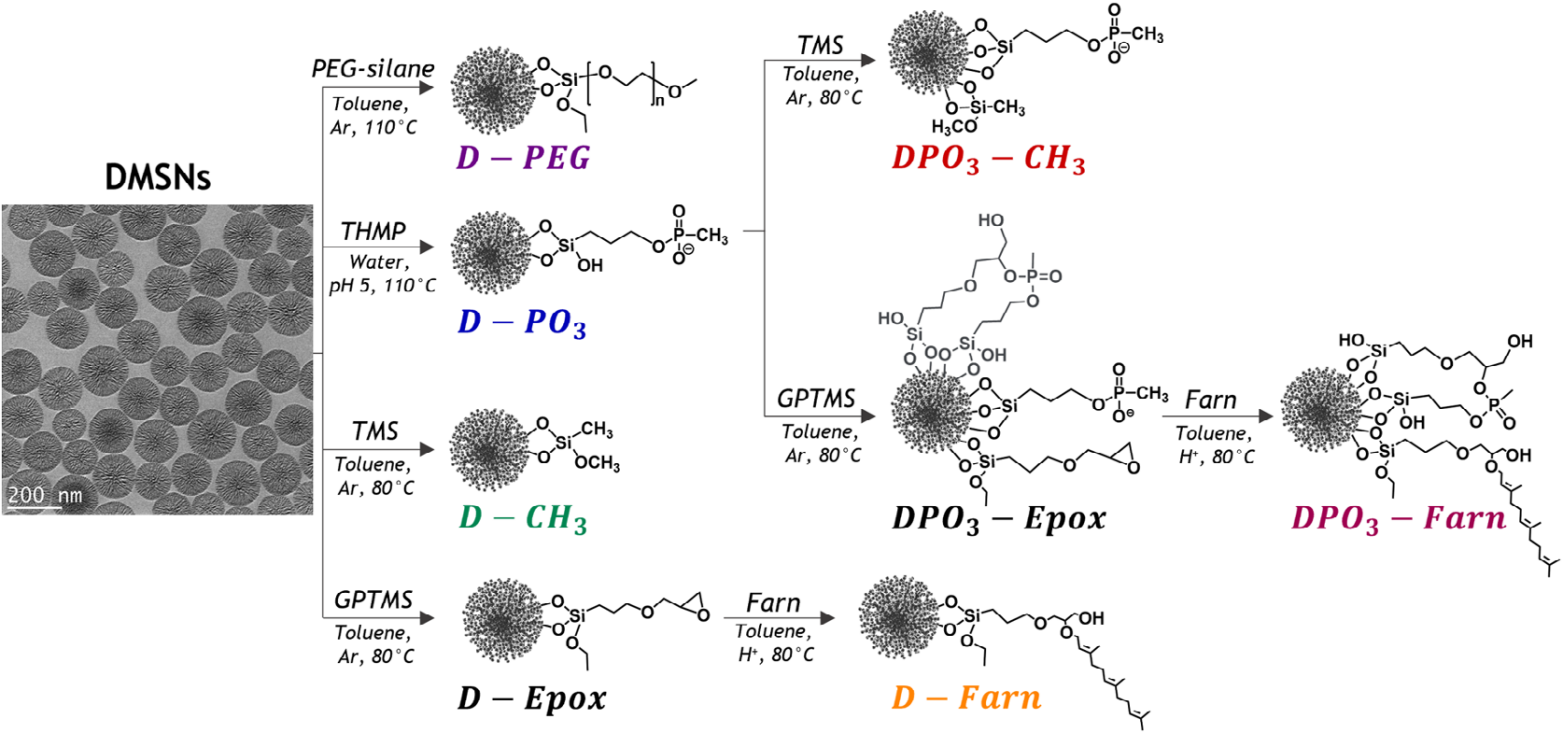
Synthetic steps for the functionalization of **DMSNs**. In the TEM image of the calcined‐**DMSNs,** the scale bar represents 200 nm (see text for acronyms).

DMSNs were synthesized from a standard protocol^[^ ^63^ ^]^ that yielded high‐quality silica nanoparticles with reproducible fine control of the size (i.e., 130 ± 10 nm) and spherical morphology, as observed by transmission electron microscopy (TEM). The calcined material (**DMSNs**) exhibited a narrow particle size distribution, as shown in the representative TEM image of **Scheme** **1**, and a hydrodynamic diameter of 162 (±1) nm, measured by dynamic light scattering (DLS). The hydrodynamic diameter of the colloidal system included both the particle size and the associated solvent shell, hence the increase with respect to the TEM measurements.

Starting from native **DMSNs**, several functionalization strategies were implemented to diversify the surface properties of the materials (Scheme 1). First, **DMSNs** were functionalized with an ethoxysilane‐functionalized polyethylene glycol (PEG‐silane, 2 kDa) using a conventional post‐grafting strategy (**D‐PEG**).^[^ ^30^ ^]^ PEG is a hydrophilic, neutral, and biocompatible polymer widely used to increase the colloidal stability of MSNs,^[^ ^26^ ^]^ as well as to prevent serum protein adsorption^[^ ^30^ ^]^ and improve mucus permeation.^[^ ^40^ ^]^ In parallel, phosphonate moieties were inserted on the particle surface (phosphonate‐grafted DMSNs (**D‐PO**_**3**_)). For this, 3‐(trihydroxysilyl)propyl‐methyl‐phosphonate silane (THMP) was used for the post‐grafting, which had the purpose of increasing not only the colloidal stability of the particles but also their negative charge and hydrophilicity. THMP‐functionalized nanoparticles have shown better control over the loading and release of positively charged drugs, as well as improved cellular uptake efficiency.^[^ ^36^ ^]^ Additionally, trimethoxymethylsilane (TMS) and (3‐glycidyloxypropyl)trimethoxysilane (GPTMS) were grafted by adapting procedures previously reported.^[^ ^30^, ^64^ ^]^ The first protocol allowed to obtain methylated DMSNs (**D‐CH**_**3**_) and increase the hydrophobic properties of the silica surface, while the second one rendered the intermediate **D‐Epox**, which resulted in terminal glycidyl moieties (i.e., epoxide) being highly reactive.^[^ ^30^ ^]^ The acid‐catalyzed alkoxylation of the epoxide‐grafted **D‐Epox** with farnesol, a natural sesquiterpene alcohol that is present in numerous essential oils of plants,^[^ ^47^, ^65^ ^]^ allowed the insertion of a highly hydrophobic acyclic hydrocarbon chain that facilitates penetration across the plasma membrane (**D‐Farn**).^[^ ^66^ ^]^ Finally, two more functionalized materials were prepared by combining the surface properties of the phosphonate‐functionalized **D‐PO**_**3**_ with the hydrophobicity of the methyl and farnesol moieties. Thus, using **D‐PO**_**3**_ as a precursor, materials exhibiting hybrid surface were obtained (**DPO**_**3**_ **‐CH**_**3**_ and **DPO**_**3**_ **‐Farn**), expecting to favor the penetration through the mucosal layer as well as the interaction with intestinal cells. For the preparation of **DPO**_**3**_ **‐Farn**, it was necessary to obtain first the intermediate **DPO**_**3**_ **‐Epox**, which subsequently reacted with farnesol according to the procedure described for **D‐Farn** synthesis (Scheme 1).

**Figure 1:**
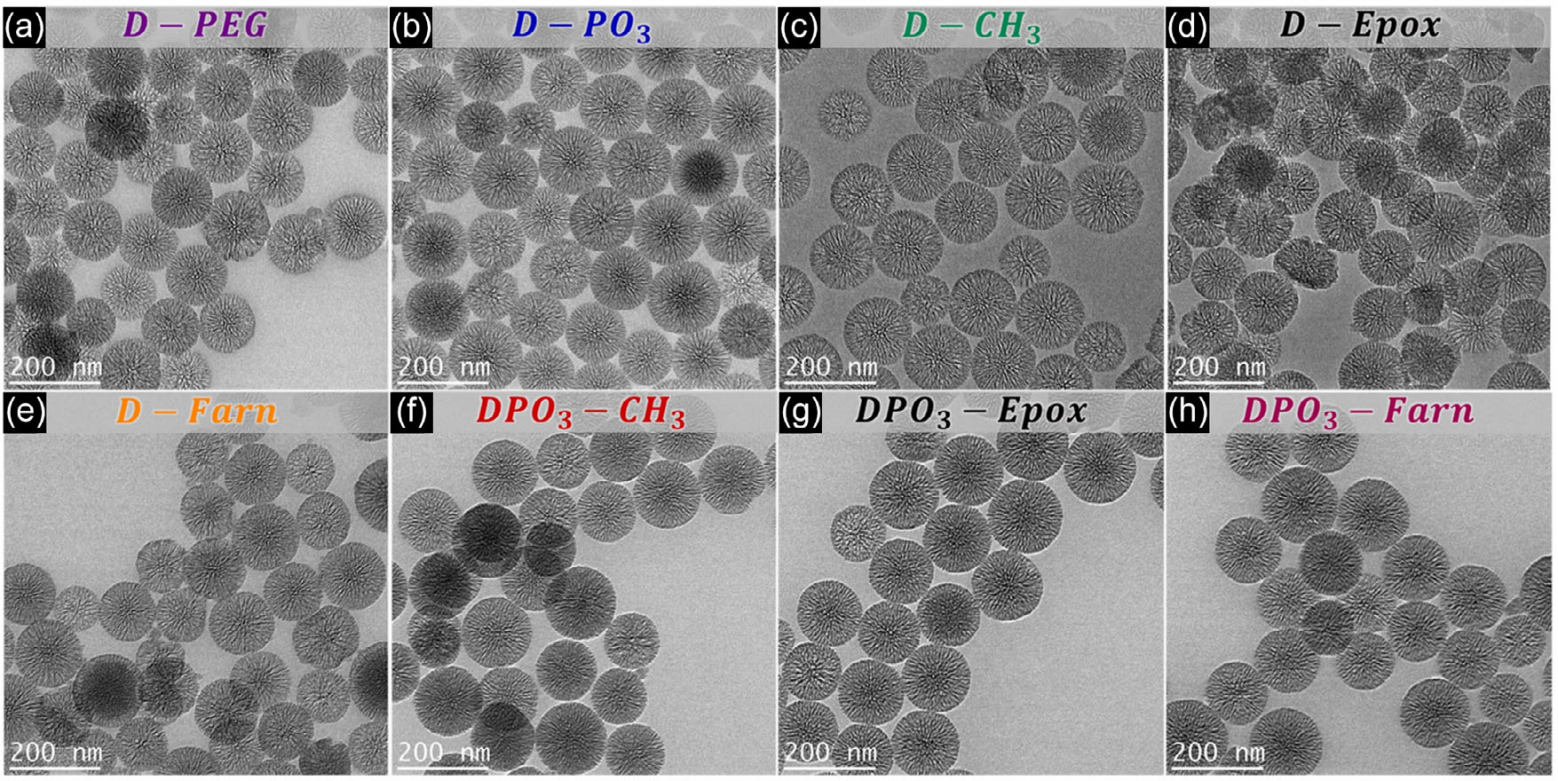
Representative transmission electron microscopy (TEM) images of functionalized DMSNs: a) **D‐PEG**, b) **D‐PO**_**3**_, c) **D‐CH**_**3**_, d) **D‐Epox**, e) **D‐Farn**, f) **DPO**_**3**_ **‐CH**_**3**_, g) **DPO**_**3**_ **‐Epox**, and h) **DPO**_**3**_ **‐Farn**. Scale bars represent 200 nm.

The functionalized particles were characterized to confirm their size, morphology, surface properties, and porosity before evaluating biological activity. TEM images of the grafted DMSNs are shown in **Figure** **1**. All materials exhibited spherical morphology without significant changes in terms of mesoporous structure and particle size, i.e., 130 (±10) nm, similar to the **DMSNs**. The increase observed in the hydrodynamic diameter of the modified particles (Figure S1a and Table S1, Supporting Information) compared to their respective precursors and the native calcined **DMSNs** (i.e., 162 ± 1 nm) was found consistent with the inclusion of organic moieties on the surface of the nanoparticles upon functionalization.

All colloidal dispersions tested via DLS exhibited narrow particle size distributions (PSDs), stable over time (Figure S1b, Supporting Information), in agreement with low polydispersity indexes (PDIs) found below 0.3 (Figure S1c, Supporting Information). Colloidal stability was also evaluated as a function of pH. In the physiological pH range (i.e., between 7.35 and 7.45), all nanoparticles, except **D‐PEG**, exhibited narrow PSD (Figure S1d, Supporting Information), which was consistent with PDI values below 0.3 (Figure S1e, Supporting Information). The higher PDI obtained for **D‐PEG** indicated lower colloidal stability associated with particle aggregation.^[^ ^25^ ^]^ The post‐grafting protocols implemented were adjusted to introduce the selected organic functions to the surface of the **DMSNs** without turning the typical negative surface charge of silica to neutral or positive values. In this way, differences observed in the interaction of the functionalized DMSNs with the intestinal cells were attributed exclusively to the effect of the surface chemistry and not to drastic differences in external charge. Although the zeta potentials of all the materials were found negative in the pH range evaluated (i.e., between 2 and 10), the variations in the observed values with respect to the calcined **DMSNs** (i.e., −34 ± 1 mV) indicated the modification of the particle surfaces upon functionalization (Figure S1f and Table S1, Supporting Information). Accordingly, a decrease in the negative surface charge of **D‐PEG** (i.e., −25 ± 2 mV) was observed, as well as the corresponding increase for **D‐PO**_**3**_ (i.e., −49 ± 2 mV), in agreement with the grafting of neutral and highly negative functions, respectively. The insertion of the methyl, epoxide, or farnesol moieties did not significantly change the surface charge of the nanoparticles. In contrast, the hybrid surfaces containing the mixture of phosphonate and methyl (i.e., **DPO**_**3**_ **‐CH**_**3**_) or phosphonate and farnesol (i.e., **DPO**_**3**_ **‐Farn**) functions exhibited intermediate values between their precursor **D‐PO**_**3**_ and the materials containing only hydrophobic moieties (i.e., **D‐CH**_**3**_ and **D‐Farn**). Detailed information on surface charge characterization can be found in Table S1 (Supporting Information). Chemical functionalization was further confirmed by thermogravimetric analysis (TGA) from the detection of mass losses (%) between 150 and 700 °C (Figure S2a, Supporting Information), which were attributed to the exothermic decomposition of the organic moieties grafted on the functionalized materials (Figure S2b, Supporting Information).

The characterization of the porosity of the silica nanoparticles can be found in Figure S3 and Table S1, Supporting Information. Predominantly, a decrease in the specific surface area (S_BET_) and pore volume with increasing amounts of the organic functions was observed (Figure S3, Supporting Information). Such reductions are consistent with the introduction of the organosilanes in the silica pores without major pore blocking, which is in line with the grafting efficiencies calculated from TGA (Table S1, Supporting Information).

**Figure 2:**
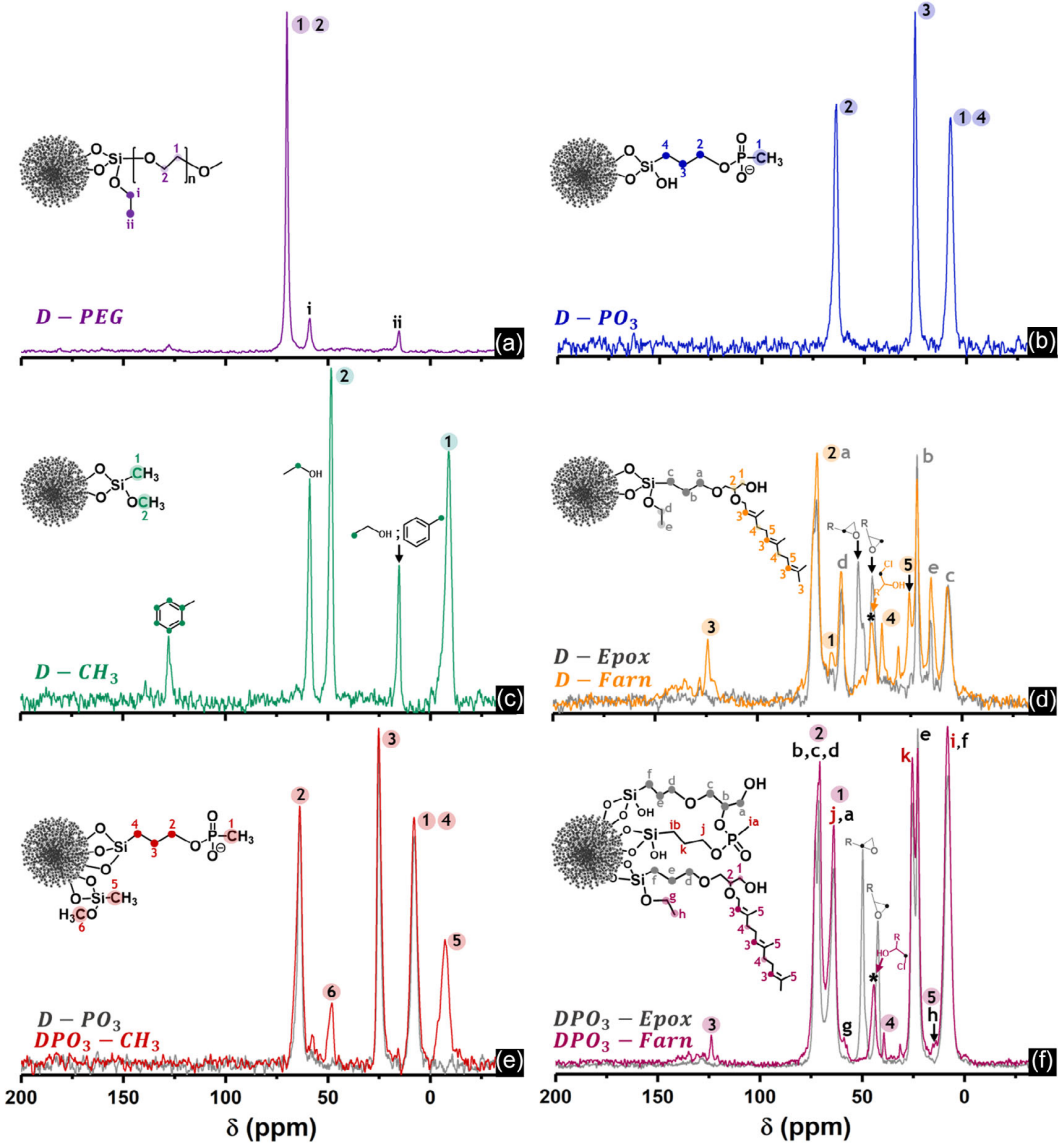
Solid‐state ^13^C CP NMR spectra of the functionalized silica nanoparticles: a) **D‐PEG**, b) **D‐PO**_**3**_, c) **D‐CH**_**3**_, d) **D‐Farn**, e) **DPO**_**3**_ **‐CH**_**3**_, and f) **DPO**_**3**_ **‐Farn**. The spectra of precursor samples (i.e., **D‐Epox**, **D‐PO**_**3,**_ and **DPO**_**3**_ **‐Epox**) were included within the respective panels (gray lines) for comparison.

The solid‐state ^29^Si cross‐polarization/magic angle spinning nuclear magnetic resonance spectroscopy (CP/MAS NMR) spectra of the functionalized materials (Figure S4a, Supporting Information) revealed the T^1^, T^2^, and T^3^ signals, which proved the successful chemical modification of **DMSNs**. No T^0^‐species were observed, confirming the absence of non‐covalently attached silanes (Table S2, Supporting Information). Moreover, the resonance signals observed in the solid‐state ^13^C CP/MAS NMR spectra of the grafted particles aligned with the structures of the organic molecules tethered on the silica surface (**Figure** **2**). In the spectrum of **D‐PEG** (Figure 2a), an intense peak was observed at 70 ppm, which was attributed to the repeating unit of the PEG polymer.^[^ ^30^, ^67^ ^]^ In the spectra of **D‐PO**_**3**_ (Figure 2b), **D‐Farn** (Figure 2d), **DPO**_**3**_ **‐CH**_**3**_ (Figure 2e), and **DPO**_**3**_ **‐Farn** (Figure 2f), the resonance signals of the carbons of the propyl chain of the silanes THMP or GPTMS were identified, in addition to carbon shifts resulting from farnesol moieties grafted on **D‐Farn** and **DPO**_**3**_ **‐Farn**. When comparing the spectra of **D‐Farn** and **DPO**_**3**_ **‐Farn** with those of the respective precursors, the absence of the oxirane signals observed previously at 44 and 51 ppm in **D‐Epox** (Figure 2d, gray lines) and **DPO**_**3**_ **‐Epox** (Figure 2f, gray lines) spectra indicated the epoxide ring‐opening upon acid‐catalyzed alkoxylation. In the spectra of **DPO**_**3**_ **‐CH**_**3**_ (Figure 2e), the chemical shifts from the THMP grafted in the precursor **DPO**_**3**_ were observed together with signals from TMS, as in the **D‐CH**_**3**_ spectrum (Figure 2c), confirming the mixture of both silanes present in the surface of the functionalized material. Toluene and ethanol residues from the TMS‐grafting were additionally detected in the **D‐CH**_**3**_ spectrum (Figure 2c). However, the remaining solvents were removed through further labeling with fluorescein isothiocyanate (FITC) and subsequent washing steps (Scheme S3, Supporting Information), not influencing, therefore, the biological activity of the methyl‐functionalized particles. This was corroborated by the absence of the corresponding resonance signals in the solid‐state ^13^C CP/MAS NMR spectra of the FITC‐labeled **D‐CH**_**3**_ (i.e., **D‐CH**_**3**_ **‐FITC**, Figure S5a, Supporting Information). The assignment of all the resonance signals observed in the ^13^C CP/MAS NMR spectra in correlation with the structures of the molecules grafted on the functionalized materials can be found in Table S3 (Supporting Information).

The presence of phosphonate species (i.e., ROP(O)CH_3_O^−^) in the ^31^P MAS NMR spectra of **D‐PO**_**3**_ and **DPO**_**3**_ **‐CH**_**3**_ (Figure S6, Supporting Information) was confirmed from the intense single peak appearing at 25 ppm (Table S4, Supporting Information). In the **DPO**_**3**_ **‐Epox** and **DPO**_**3**_ **‐Farn** spectra, a second phosphorous resonance was found at 31 ppm in addition to the ROP(O)CH_3_O^−^ signal. This strongly indicated the partial reaction of phosphonate moieties of THMP of the precursor **D‐PO**_**3**_ with the epoxide function of GPTMS upon the second functionalization step (**DPO**_**3**_ **‐Epox**, Scheme 1).^[^ ^66^ ^]^ The intensity ratio of both signals in the **DPO**_**3**_ **‐Epox** spectrum suggested the presence of a mixture of THMP functions as the primary species (i.e., ROP(O)CH_3_O^−^), together with a cyclic THMP–GPTMS conjugate. Nevertheless, the ^13^C CP/MAS NMR spectra of **DPO**_**3**_ **‐Epox** (Figure 2f) confirmed the presence of epoxide moieties still closed (i.e., oxirane ring) for the subsequent functionalization with farnesol. After the reaction of **DPO**_**3**_ **‐Epox** with farnesol (**DPO**_**3**_ **‐Farn**), the ROP(O)CH_3_O^−^ signal in the ^31^P MAS NMR spectrum of the **DPO**_**3**_ **‐Farn** was suppressed, and the intense resonance observed at 31.6 ppm was attributed to the THMP–GPTMS conjugate attached to silica.

The acidic conditions required for the epoxide ring‐opening and further reaction with farnesol favored the interaction of free GPTMS moieties with the grafted THMP and, thus, the formation of the cyclic conjugate as the major species. To test this hypothesis and corroborate the structure proposed for **DPO**_**3**_ **‐Farn**, the oxirane moieties of **DPO**_**3**_ **‐Epox** were opened in an acid medium following the same conditions adjusted for the acid‐catalyzed alkoxylation but without adding farnesol (Scheme S1, Supporting Information). After the addition of HCl (37%), the resonances previously observed at 42.2 and 49.7 ppm in the solid‐state ^13^C CP/MAS NMR spectrum of **DPO**_**3**_ **‐Epox** (Figure 2f, gray line) disappeared, which confirmed the opening of the oxirane ring of GPTMS (Figure S5b, Supporting Information). A new resonance peak was detected at 44 ppm in the spectrum of the control sample (i.e., **DPO**_**3**_ **‐Epox + HCl**), also observed in the spectra of **D** **‐Farn** (Figure 2d) and **DPO**_**3**_ **‐Farn** (Figure 2f) and attributed to the formation of a chlorohydrin derivative.^[^ ^68^ ^]^ The generation of chlorohydrins from the reaction of epoxides with HCl in anhydrous solvents has previously been reported.^[^ ^68^, ^69^ ^]^ These derivatives have exhibited enhanced biological properties in pharmaceutical applications.^[^ ^70^ ^]^ The ^31^P MAS NMR spectrum of the control sample **DPO**_**3**_ **‐Epox + HCl** showed comparable resonance signals and intensity ratios as in the **DPO**_**3**_ **‐Farn** spectrum (Figure S6, Supporting Information), which confirmed the reaction of the epoxide moieties with the grafted THMP.

The functionalization with farnesol was additionally carried out following an alternative protocol. First, GPTMS was reacted with an excess of farnesol in an acidic medium. The obtained GPTMS‐Farn conjugate was then added to **DMSNs** or **D‐PO**_**3**_, which rendered the control samples **D‐Farn‐(1 step)** and **DPO**_**3**_ **‐Farn‐(1 step)**, respectively (Scheme S2, Supporting Information). Solid‐state ^29^Si CP/MAS and ^13^C CP/MAS NMR spectra of the synthetic controls **DPO**_**3**_ **‐Epox + HCl**, **D‐Farn‐(1 step)**, and **DPO**_**3**_ **‐Farn‐(1 step)** can be found in Figure S4b and S5 (Supporting Information), respectively, and the assigned chemical shifts in Table S2 and S3 (Supporting Information). In addition to the GPTMS‐Farn resonance (32.9 ppm), the ^31^P MAS NMR spectrum of **DPO**_**3**_ **‐Farn‐(1 step)** showed an intense signal at 25.4 ppm, which indicated remaining ROP(O)CH_3_O^−^ moieties from THMP due to the reaction of most of the oxirane functions of GPTMS with farnesol instead of with the phosphonate silane grafted on the particles (Figure S6, Table S4, Supporting Information). This contrasted with the structure composition of the organic functions grafted on **DPO**_**3**_ **‐Farn**, highlighting the importance of strict control over reaction conditions to guarantee homogeneous surface modification.

The activity profiling of the materials obtained after functionalization, i.e., **D‐PEG**, **D‐PO**_**3**_, **D‐CH**_**3**_, **D‐Farn**, **DPO**_**3**_ **‐CH**_**3**_, **DPO**_**3**_ **‐Farn**, together with the non‐functionalized **DMSNs**, was characterized using a coculture of Caco‐2/HT29‐MTX‐E12 cells as intestinal model. After differentiation, these cells form a polarized 3D epithelial layer, characterized by a high transepithelial electrical resistance and functional properties resembling the intestinal barrier; these include mucus secretion, TJs’ expression, apical and basolateral sides, and a brush border with microvilli on the apical surface.^[^ ^25^, ^71^ ^]^ The evaluation of the chemical surface–activity relationship was designed considering the preservation of intestinal barrier integrity, as well as the intra‐pericellular localization of the particles and cell–cell organization. To isolate the contribution of the mucus in the biological performance of the nanomaterials, experiments were additionally carried out by removing the mucus layer formed after 7 days of differentiation (− Mucus). For this, *N‐*acetylcysteine was used following a previously described procedure.^[^ ^72^ ^]^ Maintenance of viability after 6 h incubation of Caco‐2/HT29‐MTX‐E12 cells with **DMSNs** and functionalized particles was confirmed by the Neutral Red assay (Figure S7, Supporting Information).^[^ ^73^ ^]^ For all materials, no significant decrease in the signal of the dye bound to the lysosomes of viable cells was detected with respect to the controls (i.e., non‐treated cells). Cell viability was maintained even after the removal of the mucus layer, followed by nanoparticle treatment (600 μg mL^−1^).

**Figure 3:**
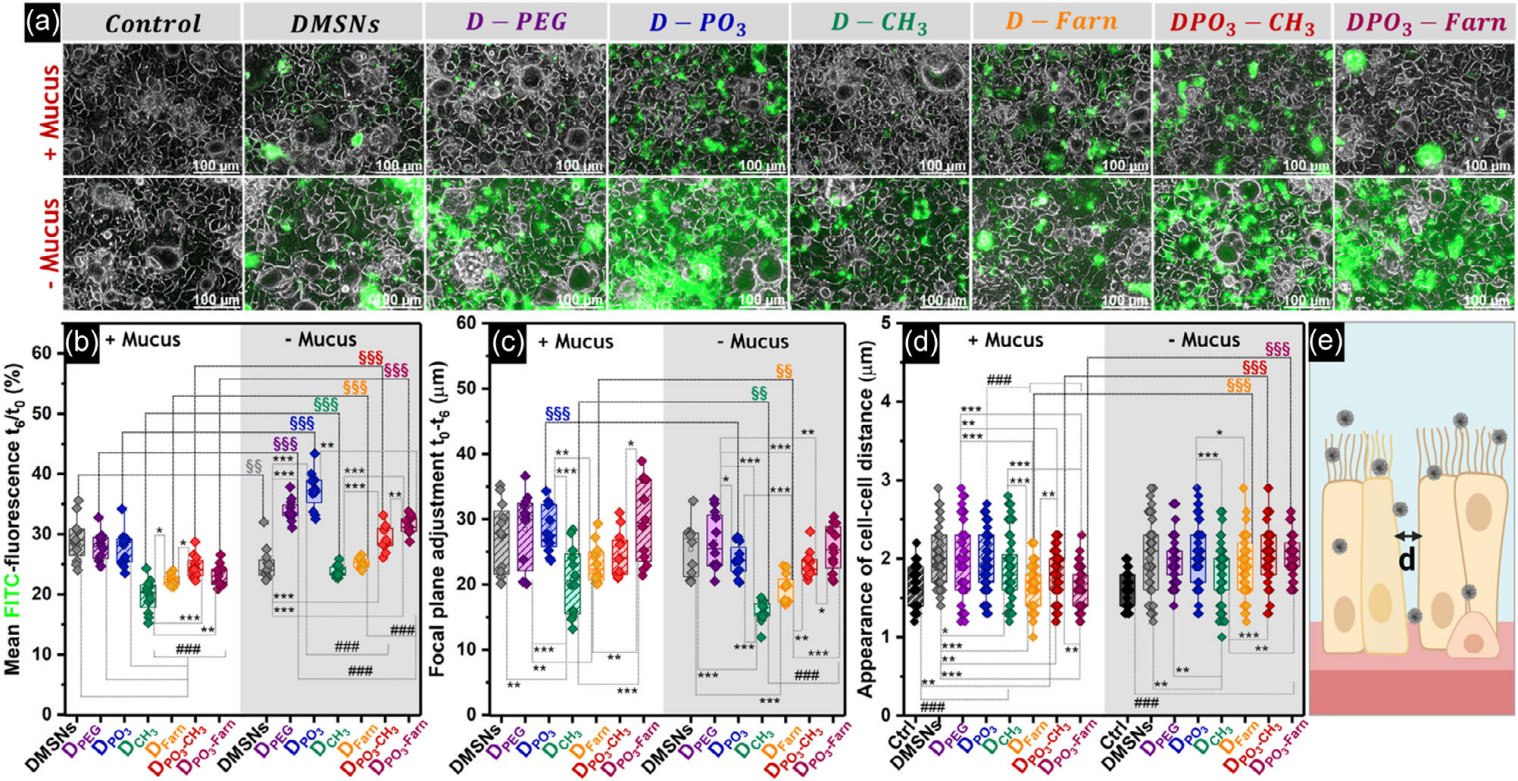
a) Appearance of Caco‐2/HT29‐MTX‐E12 cells after treatment with FITC‐labeled particles (20× magnification). The control corresponds to non‐treated cells incubated in complete cell culture medium. Scale bars represent 100 μm. b) Quantification of the residual FITC fluorescence due to particle–cell interactions (%) with and without the mucus layer. c) Quantification of the focal plane adjustment obtained from the difference between the optical parameters set immediately after cell treatment with the FITC‐labeled particles (*t*_0_) and after 6 h of incubation (*t*_6_) with and without the mucus layer (*t*_0_ *–t*_6_). At least 18 paired images were analyzed (*n* = 18). d) Appearance of cell–cell distance of Caco‐2/HT29‐MTX‐E12 cells after 6 h of incubation with silica particles, measured from n ≥ 60 cells. Statistically significant differences according to one‐way ANOVA and Fisher tests when treatments are compared in the presence or absence of mucus (*), when the effect of mucus is compared for the same treatment (§), or when a specific treatment is compared with a group of others (#), are indicated with */§/# (*p* < 0.05), **/§§/## (*p* < 0.01), or ***/§§§/### (*p* < 0.001). For each condition, data were obtained from three independent cell preparations (biological triplicates) measured in technical duplicates. e) Schematic representation of cell accommodation upon particle treatment created with BioRender.com.

The interaction of FITC‐labeled‐functionalized materials (Scheme S3, Supporting Information) with the intestinal cells was evaluated via live cell imaging after 6 h incubation at 37 °C (**Figure** **3**a). The analysis of phase contrast images in combination with fluorescence acquisition (10× magnification) was performed according to a protocol recently published.^[^ ^25^ ^]^ Cells were imaged immediately after the application of the particles (*t*_0_), as well as after 6 h incubation (*t*_6_). Loose particles were removed by gentle washings, and two paired images were subsequently acquired at *t*_6_, one maintaining the exact coordinates and focus as that for *t*_0_ and the second one refocusing on the particle layer. The fluorescence signal variation after nanoparticle treatments was quantified in relation to the initial FITC fluorescence detected at *t*_0_. Thus, the residual values calculated (*t*_6_/*t*_0_, %) were considered as a measure of particle–cell interaction in the presence or absence of the mucus layer (Figure 3b). The *t*_0_ images additionally confirmed that the nanoparticles were homogeneously distributed at the beginning of the experimental procedure (Figure S8, Supporting Information).

In the presence of mucus, **DMSNs**, **D‐PEG,** and **D‐PO**_**3**_ treatments returned similar residual fluorescence values (Figure 3b), which were significantly higher than those corresponding to hydrophobic nanoparticles (i.e., **D‐CH**_**3**_ and **D‐Farn**) or particles with “hybrid” surface compositions (i.e., **DPO**_**3**_ **‐CH**_**3**_ and **DPO**_**3**_ **‐Farn**). Mucus removal before nanoparticle incubation returned a significant increase of the residual fluorescence for all the treatments with functionalized silica, an effect not observed for native **DMSNs** (Figure 3b). Interestingly, the insertion of methyl and farnesol moieties in phosphonate‐functionalized particles (**DPO**_**3**_ **‐CH**_**3**_ and **DPO**_**3**_ **‐Farn**, respectively) improved particle–cell interactions in comparison to the non‐phosphonate **D‐CH**_**3**_ and **D‐Farn** (− Mucus, Figure 3b). These observations confirmed the crucial contribution of the mucus as a first barrier that limits the transport/availability of nanoparticles before possible interaction with the cell surface.

After 6 h incubation, the difference between the focus of the two images acquired at the exact coordinates (*t*_0_ – *t*_6_) was used to calculate the penetration of the particles toward the cell monolayer (Figure 3c). A broader distribution of the data was observed when experiments were performed in the presence of mucus. This can be possibly attributed to particle retention within the respective layer.^[^ ^25^ ^]^ **DMSNs**, **D‐PEG**, and **D‐PO**_**3**_ exhibited high motility through the mucus (i.e., on average, 27, 28, and 29 μm, respectively). The same trend was also observed after mucus removal; hydrophilic functions (**DMSNs**, **D‐PEG**, and **D‐PO**_**3**_ surfaces) seemed to favor diffusion through the residual mucus layer. Hence, **D‐CH**_**3**_ and **D‐Farn** exhibited the most moderate focal plane adjustment (i.e., on average, 16 and 19 μm, respectively), which was attributed to stronger interactions of their hydrophobic moieties with the residual mucin proteins. In turn, **DPO**_**3**_ **‐CH**_**3**_ and **DPO**_**3**_ **‐Farn** showed similar performance to their precursor **D‐PO**_**3**_ (± Mucus; Figure 3c).

To further describe the effect of the mucus in the biological performance of the MSNs, immunofluorescence staining of the mucin protein MUC5AC (Figure S9 and S10, Supporting Information) was carried out after incubation with FITC‐labeled materials (i.e., **DMSNs**, **D‐PO**_**3**_, and **DPO**_**3**_ **‐Farn**). For both the control (i.e., non‐treated cells) and all the particle treatments, a thin layer of mucus could be detected in the cellular proximity (± Mucus, Figure S9a,b, Supporting Information). The evaluation of orthogonal views obtained from the corresponding 3D reconstructions allowed the visualization of the distribution of the MUC5AC staining also along the *z*‐axis (Figure S9c, Supporting Information). Signals detected up to the apical zone of the cells and on top of the coculture were quantified from the respective intensity projections (Figure S9d,e, Supporting Information). Incubation with the **DMSNs**, **D‐PO**_**3**_, and **DPO**_**3**_ **‐Farn** particles did not affect the signal of MUC5AC (Figure S9d,e, Supporting Information). Additionally, these data confirmed that the treatment with *N*‐acetylcysteine (− Mucus) significantly reduced the signal of MUC5AC above the cells, corresponding to a 38% decrease with respect to cells containing mucus (Figure S9e, Supporting Information). Focusing on the particle quantification, in the presence of mucus, FITC‐labeled **D‐PO**_**3**_ returned the strongest signal (Figure S9f, Supporting Information). Upon preincubation with *N*‐acetylcysteine, **D‐PO**_**3**_ and **DPO**_**3**_ **‐Farn** materials exhibited higher FITC fluorescence intensities in comparison to the non‐functionalized **DMSNs** (Figure S9f, Supporting Information). These responses generally aligned with the data acquired from live cell imaging (Figure 3b) and confirmed the interplay between the mucus layer and functionalization strategies in modulating particle–cell interactions. Along these lines, analysis of *z*‐axis plot profiles obtained from these 3D‐reconstruction images (Figure S10a, Supporting Information) confirmed that the relative position of the MUC5AC distribution (i.e., on average, 1.9 μm above cell nuclei for the controls) was significantly modified only by the application of **D‐PO**_**3**_ (Figure S10b, Supporting Information). Furthermore, MUC5AC thickness along the *z*‐axis in the cellular layer (i.e., on average, 7.2 μm for the controls) was reduced by the **DMSNs** and **D‐PO**_**3**_ independently from the pre‐incubation with *N*‐acetylcysteine (Figure S10c, Supporting Information). In turn, *N*‐acetylcysteine treatment significantly reduced the thickness of the mucus distributed on top of the coculture (Figure S10d, Supporting Information), correlating with the quantification of the MUC5AC fluorescence intensity on such region (Figure S9e, Supporting Information).

**Figure 4:**
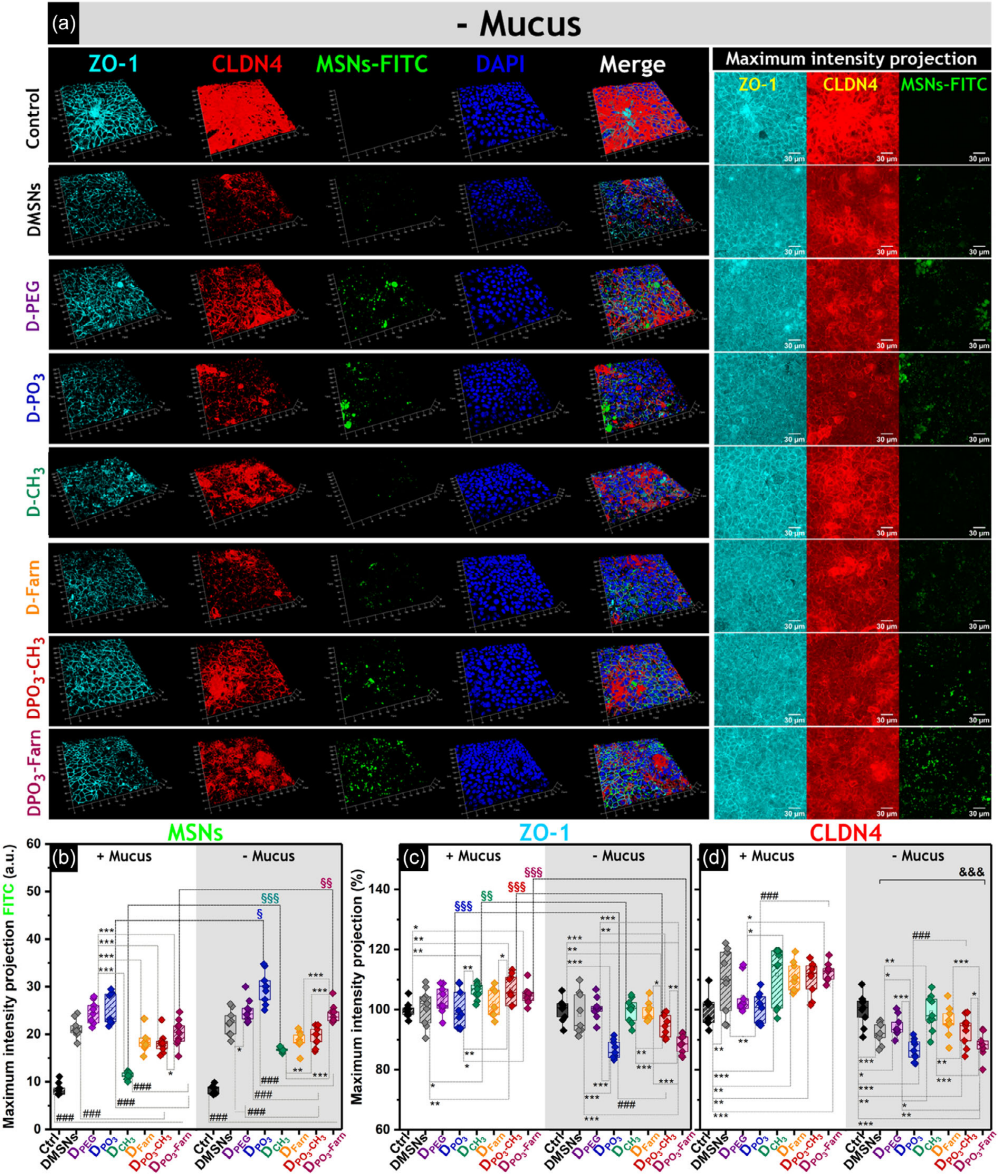
Representative immunofluorescence staining of ZO‐1 (cyan) and CLDN4 (red). The 3D reconstructions (63x magnification) were obtained by z‐stack imaging after incubation with *N*‐acetylcysteine (− Mucus) and 6 h treatment with FITC‐labeled silica nanoparticles (green). The control corresponds to non‐treated cells incubated in complete cell culture medium. The scale bar segmentation is 10 μm, and the nuclei are represented in blue (DAPI staining). Scale bars of the Z‐projection images of ZO‐1, CLDN4, and FITC channels for corresponding 3D reconstructions stand for 30 μm. Quantification of the mean fluorescence intensity of b) FITC, c) ZO‐1, and d) CLDN4, using maximum intensity projection images obtained from 3D reconstructions (*n* = 9). Statistically significant differences according to one‐way ANOVA and Fisher tests when treatments are compared in the presence or absence of mucus (*), when the effect of mucus is compared for the same treatment (§), or when a specific treatment is compared with a group of others (#), are indicated with */§/# (*p* < 0.05), **/§§/## (*p* < 0.01), or ***/§§§/### (*p* < 0.001). For CLDN4‐staining, maximum intensity projections corresponding to particle treatments after mucus removal were significantly lower than the respective values measured with mucus, which was indicated by &&& (*p* < 0.001). Data sets were obtained from three independent cell preparations (biological triplicates).

Since the functionalized nanoparticles showed differential interplay with the intestinal cell model, the potential to alter the organization of the epithelial layer was additionally explored. For this, image analysis was focused on the cell–cell junctions, postulating a possible interaction of silica with the outer surface of the cell membrane. Phase contrast images taken at 20× magnification after 6 h incubation with **DMSNs** and with all functionalized materials allowed the measurement of the appearance of the cell–cell distances (Figure 3d,e). In the presence of mucus, **DMSNs**, **D‐PEG**, **D‐PO**_**3**_, and **D‐CH**_**3**_ treatments significantly incremented the intercellular distances as compared to controls (i.e., non‐treated cells). For **D‐Farn**, **DPO**_**3**_ **‐CH**_**3**_, and **DPO**_**3**_ **‐Farn**, reduced activity can be considered coherent with the notion that limited diffusion through the mucus resulted in a reduced particle–cell interaction with minimal modification of the cell layer architecture. However, a significant increase in cell–cell distances could be observed for all treatments when the mucus layer was previously removed (− Mucus; Figure 3d). To further characterize particle–cell interactions in detail, the expression and distribution of TJ proteins were evaluated. Thus, immunofluorescence staining of Zonula occludens‐1 (ZO‐1) and Claudin‐4 (CLDN4) was performed after 6 h treatment with FITC‐labeled particles in the presence of mucus (Figure S11, Supporting Information) or after its reduction with *N*‐acetylcysteine (**Figure** **4**a).

ZO‐1 is described as a significant cytoplasmic component of TJs since it binds most transmembrane and cytoskeletal proteins, exhibiting the intrinsic capacity to cross‐link them into bigger arrays.^[^ ^6^ ^]^ Consequently, other TJ constituents like occludin and claudins can only form organized strands at the cell's apical end when ZO‐1 incorporates them into the junction complex.^[^ ^59^ ^]^ On the other hand, claudins are considered essential in enabling paracellular transport, playing a pivotal role in TJ morphology and stabilization.^[^ ^6^, ^59^ ^]^ Particularly, CLDN4 is distributed on the lateral surfaces of intestinal epithelial cells, where it assists in the regulation of ions and macromolecule movement across the intestinal epithelium, thus contributing to the modulation of intestinal permeability.^[^ ^6^, ^74^ ^]^

ZO‐1, CLDN4, and FITC‐fluorescence signals were quantified from the maximum intensity projection of the respective channels in the 3D reconstructions of the cell monolayers (Figure 4a and S11, Supporting Information). FITC fluorescence was used to benchmark the interaction of the MSNs with the intestinal cells after 6 h treatment (Figure 4b). Since the data pattern largely aligned with the quantification values previously obtained from fluorescence imaging at lower magnification (10×, Figure 3b), as well as with the FITC‐fluorescence quantification from MUC5AC staining experiments (Figure S9f, Supporting Information), it can be postulated that differences among the treatments are more likely related to the differential surface chemistry of the particles rather than to quantification artifacts or bias associated with the selection of optical fields.

Overall, the quantification of the TJs staining in the presence of mucus showed no significant reduction upon nanoparticle treatments with respect to the control (i.e., non‐treated cells). This was observed for both ZO‐1 (Figure 4c) and CLDN4 (Figure 4d), where rather an increase in the maximum fluorescence was detected for **D‐Farn** (Figure 4d), **D‐CH**_**3**_, **DPO**_**3**_ **‐CH**_**3**_, and **DPO**_**3**_ **‐Farn** treatments (Figure 4c,d), i.e., particles containing hydrophobic functional groups grafted on their surfaces. Aligning to this, no alteration of the transepithelial electrical resistance (TEER) was measured after 6 and 24 h incubations with functionalized particles (Figure S12a, Supporting Information). However, subsequent application of Lucifer Yellow revealed a significant increase in dye permeability with respect to the control (i.e., non‐treated cells), which was exclusive to the treatments with phosphonate‐functionalized nanoparticles, i.e., **D‐PO**_**3**_, **DPO**_**3**_ **‐CH**_**3**_, and **DPO**_**3**_ **‐Farn** (Figure S12b, + Mucus). This might relate to cell accommodation in the presence of phosphonate moieties, which could ultimately enhance epithelial permeability. Along these lines, the removal of the mucus layer intensified such effects, possibly fostering the interaction of the nanoparticles with the cell surface. In this respect, for **D‐PO**_**3**_, **DPO**_**3**_ **‐CH**_**3**_, and **DPO**_**3**_ **‐Farn** treatments, the fluorescence intensity corresponding to ZO‐1 staining decreased compared to the control (Figure 4c, − Mucus) while remaining unchanged for the other particle treatments. For CLDN4 staining (Figure 4d, − Mucus), a decrease in fluorescence intensity was observed for all treatments, except for those involving the most hydrophobic **D‐CH**_**3**_ and **D‐Farn** particles, which exhibited similar values of fluorescence intensity as the controls. These results strongly indicated a selective interaction of the nanoparticles with the TJs, highly dependent on the surface chemistry, with the phosphonate functions (i.e., **D‐PO**_**3**_, **DPO**_**3**_ **‐CH**_**3**_, and **DPO**_**3**_ **‐Farn**) exhibiting a more pronounced effect on TJs organization.

Since the rearrangement of TJ proteins in the presence of functionalized materials seemed to be strongly related to the barrier integrity, the distribution profiles of both ZO‐1 and CLDN4 stainings along the *z*‐axis were also analyzed (± Mucus; Figure S13a,b, Supporting Information). This approach was applied to gain insights into the effects of the materials on the localization of the junctional proteins along the cell–cell interface. Here, it was possible to observe that the distance at which the highest fluorescence intensity of the ZO‐1 staining was found (i.e., relative *z*‐position, 1.8 μm above cell nuclei for non‐treated cells) increased with respect to the nuclei position upon treatment with **D‐PO**_**3**_ (+ Mucus, Figure S13c, Supporting Information). This was especially visible after mucus removal and subsequent incubation with the phosphonated material (i.e., 3.2 μm above cell nuclei), as well as with **D‐CH**_**3**_ (i.e., 2.3 μm above cell nuclei). Instead, the relative z‐position of CLDN4 was less affected by the treatments (Figure S13d, Supporting Information), displaying the maximum fluorescence intensity at 0.6 μm above the nuclei. In the presence of mucus, the increase in the staining signal range along the *z*‐axis (i.e., z‐distance range covered by fluorescence signal), both for ZO‐1 (Figure S13e, Supporting Information) and for CLDN4 (Figure S13f, Supporting Information), closely agreed with the quantification of the expression of the TJs proteins discussed above (Figure 4c,d). Additionally, the mucus removal increased the thickness of the ZO‐1 signal in control cells; when the cells were incubated with **D‐PO**_**3**_, **D‐CH**_**3**_, and **DPO**_**3**_ **‐CH**_**3**_, the signal distribution was reduced in comparison to the same treatments in the presence of mucus (± Mucus; Figure S13e, Supporting Information). Overall, the apparent redistribution of ZO‐1 upon incubation with the MSNs could be interpreted as a differential affinity for the nanoparticles, which might modify the binding to TJs, ultimately impacting cell architecture and epithelial permeability.^[^ ^59^ ^]^

**Figure 5:**
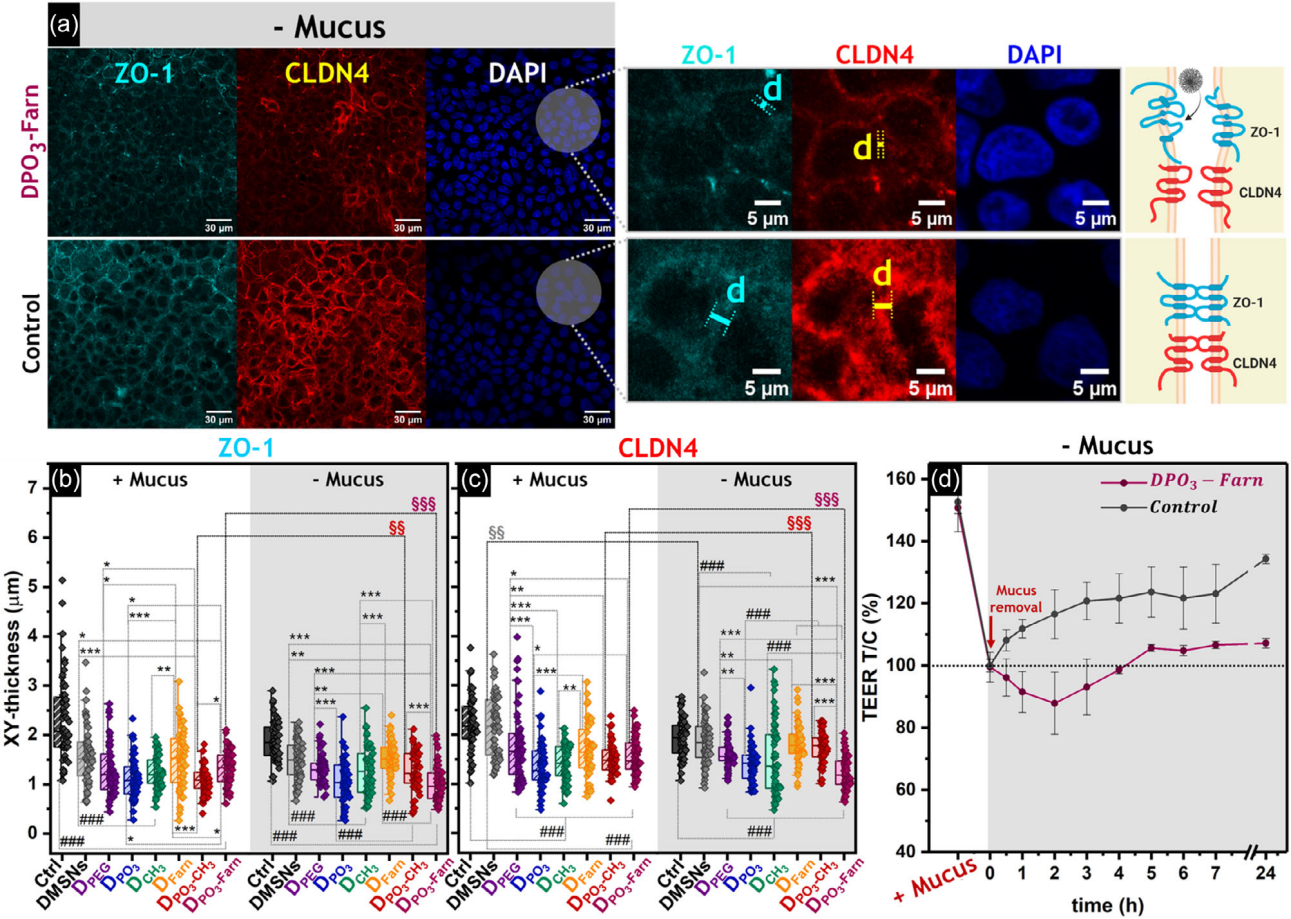
a) Representative immunofluorescence staining of TJs after 6 h treatment of Caco‐2/HT29‐MTX‐E12 cells with **DPO**_**3**_ **‐Farn** in the absence of mucus. The control corresponds to non‐treated cells incubated in complete cell culture medium. ZO‐1 staining is represented in cyan, CLDN4 in red, and the nuclei (stained with DAPI) in blue. Scale bars stand for 30 μm. Graphical representations of the XY‐thickness (d, μm) measured for ZO‐1 and CLDN4 stainings are included in the zoomed‐in images, whose scale bars stand for 5 μm. Schematic representations of the ZO‐1 and CLDN4 distributions were created with BioRender.com. Quantification of XY‐thickness (μm) of b) ZO‐1 and c) CLDN4 stainings after 6 h treatment with silica nanoparticles. Each dataset resulted from the analysis of *n* ≥ 60 cells from three independent preparations (biological triplicates). Statistically significant differences according to one‐way ANOVA and Fisher tests when treatments are compared in the presence or absence of mucus (*), when the effect of mucus is compared for the same treatment (§), or when a specific treatment is compared with a group of others (#), are indicated with */§/# (*p* < 0.05), **/§§/## (*p* < 0.01), or ***/§§§/### (*p* < 0.001). d) Transepithelial electrical resistance (TEER) upon treatment with nonfluorescent **DPO**_**3**_ **‐Farn** or in cell culture medium (control, i.e., non‐treated cells) in the absence of mucus. TEER was measured before cell treatment (+ Mucus), and the TEER (%) values were calculated as a percentage of the TEER obtained immediately after mucus removal (dashed line). All data were obtained from three independent cell preparations (biological triplicates) and are presented as mean ± standard deviations (*n* = 3). Student's t‐test confirmed significant differences between the TEER values corresponding to **DPO**_**3**_ **‐Farn** treatment and control in all the time slots measured after mucus removal (*p* < 0.001).

Focusing further on the cell–cell junctions, additional imaging was performed after the incubation of the Caco‐2/HT29‐MTX‐E12 cells with the nanoparticles (**Figure** **5** and S14, Supporting Information). The focal plane of the 2D optical fields acquired was adjusted by taking the cell nuclei as a reference (i.e., DAPI staining), and the distribution of both ZO‐1 and CLDN4 was further evaluated. As illustrated representatively for the treatment with **DPO**_**3**_ **‐Farn** (− Mucus, Figure 5a and zoomed images), it was possible to appreciate that the thickness of the fluorescence signal decreased considerably for both TJs stainings with respect to the control (i.e., non‐treated cells). This response seemed coherent for all treatments with functionalized particles for both ZO‐1 (Figure 5b) and CLDN4 (Figure 5c).

In the presence of mucus, the 2D appearance of both ZO‐1 and CLDN4 (Figure 5b,c) correlated with the relative mean intensities (%) of the immunofluorescence analysis (Figure S15, Supporting Information). The most notable effect was observed upon incubation with **D‐PO**_**3**_ in the presence of mucus (Figure 5b,c and S15a,b, Supporting Information). This can be considered in agreement with the most efficient redistribution of the ZO‐1 and CLDN4 along the *z*‐axis triggered by this treatment (**D‐PO**_**3**_, Figure S13e,f, Supporting Information) and possibly attributed to a better interaction of the phosphonated particles with the cells. In the absence of mucus, **DPO**_**3**_ **‐Farn** exhibited the most significant response in terms of reduction of TJ signal intensities (Figure S15, Supporting Information) and appearance (Figure 5b,c). Time‐dependent measurement of the TEER values after **DPO**_**3**_ **‐Farn** treatment (− Mucus, Figure 5d) was consistent with these observations, indicating an increase in monolayer permeability in the presence of nanoparticles. The TEER values significantly dropped after mucus removal and then recovered progressively for the control (i.e., non‐treated cells, − Mucus). However, for the cells incubated with **DPO**_**3**_ **‐Farn**, a sustained decrease of the TEER was observed within the first 2 h of incubation (where the minimum TEER was measured). Despite the apparent gradual recovery after the first 2 h of incubation with **DPO**_**3**_ **‐Farn**, the corresponding TEER values remained lower with respect to the control. Data collected after 24 h treatment aligned with the outcome of the Lucifer Yellow measurements (Figure S12b, Supporting Information, − Mucus), confirming the increased epithelial permeability in the presence of **DPO**_**3**_ **‐Farn**.

Altogether, the results obtained support an unquestionable role for the surface chemistry of the nanoparticles in modulating intestinal cells’ organization by affecting the distribution of TJs and increasing intercellular distances. Obviously, these responses might be mediated by several contributions. For example, selective chemical interactions could transiently regulate intestinal permeability by cytoskeletal rearrangement, as in the case of actin disruption.^[^ ^53^ ^]^ Indeed, this mechanism could be complementary or further foster the transcellular transport via receptor‐mediated endocytosis.^[^ ^58^ ^]^ With the purpose of deepening the understanding of the mechanisms behind the chemically modulated interactions of silica nanoparticles with the intestinal cells, functionalized particles were applied to the cells in the presence of selected molecules that are known to modify the architecture of the cell membrane, according to an experimental layout recently reported.^[^ ^25^ ^]^ Pitstop 2 and methyl‐β‐cyclodextrin (mβCD) were used as inhibitors of the clathrin and caveolae receptors, respectively.^[^ ^75^ ^]^ Pitstop 2 acts via blocking ligand access to the clathrin terminal domain and also can break down the nuclear pore complex (NPC) permeability barrier, decreasing importin‐β binding along with alteration of the NPC ultrastructure.^[^ ^76^, ^77^ ^]^ MβCD supports cholesterol depletion, which alters caveolae organization and membrane fluidity.^[^ ^78^, ^79^ ^]^ The dietary lipid oleic acid (OA) was also included in the layout since it alters membrane fluidity and rearranges the cytoskeleton of intestinal cells, modulating their mechanosensory apparatus.^[^ ^80^ ^]^

**Figure 6:**
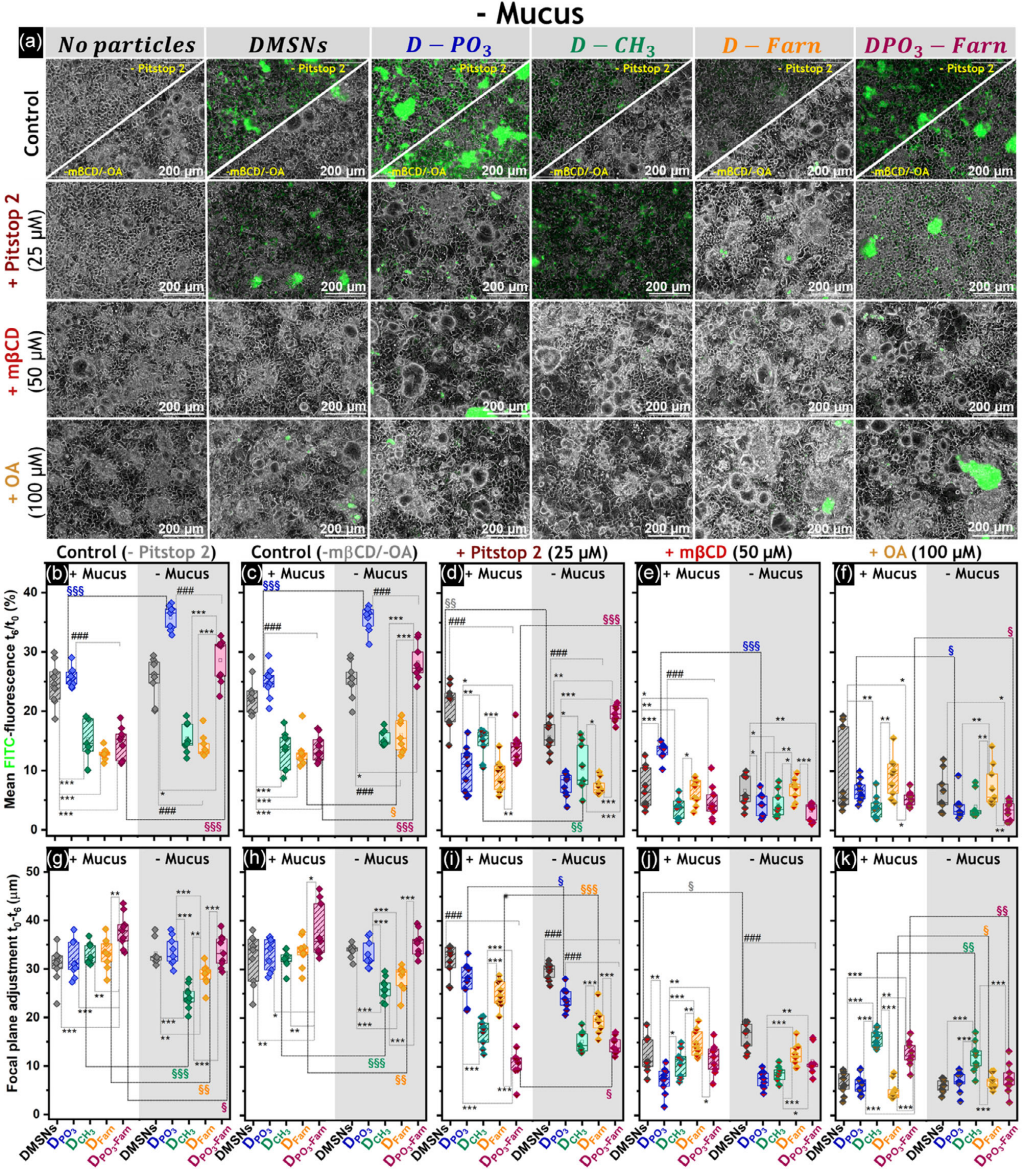
a) Appearance of Caco‐2/HT29‐MTX‐E12 cells after preincubation with Pitstop 2 (25 μM, 0.3% DMSO), mβCD (50 μM, 0.05% DMSO), and OA (100 μM, 0.03% DMSO) followed by 6 h treatment with FITC‐labeled particles in the absence of mucus (10× magnification). Scale bars stand for 200 μm. b–f) Quantification of the residual FITC fluorescence due to particle–cell interactions (%) with and without the mucus layer in control conditions, b) ‐ Pitstop 2 or c) ‐ mβCD/‐ OA, or after preincubation with d) Pitstop 2, e) mβCD, or f) OA, followed by particle treatments. g–k) Quantification of the focal plane adjustment obtained from the difference between the optical parameters set immediately after FITC‐labeled nanoparticles treatment (*t*_0_) and after 6 h incubation (*t*_6_) with or without mucus layer in control conditions, g) ‐ Pitstop 2 or h) ‐ mβCD/‐ OA, or after preincubation with i) Pitstop 2, j) mβCD, or k) OA, followed by particle treatments. Experiments were performed in biological triplicates, and at least 9 paired images were analyzed (before and after focus adjustment, *n* = 9). Statistically significant differences according to one‐way ANOVA and Fisher tests when treatments are compared in the presence or absence of mucus (*), when the effect of mucus is compared for the same treatment (§), or when a specific treatment is compared with a group of others (#), are indicated with */§/# (*p* < 0.05), **/§§/## (*p* < 0.01), or ***/§§§/### (*p* < 0.001).

The experimental conditions, including the working concentrations of Pitstop 2, mβCD, and OA, were chosen in order to preserve cell viability.^[^ ^25^ ^]^ After the treatment with such compounds, the cells were incubated with FITC‐labeled nanoparticles, i.e., **DMSNs**, **D‐PO**_**3**_, **D‐CH**_**3**_, **D‐Farn**, and **DPO**_**3**_ **‐Farn**, followed by live cell imaging (phase contrast and fluorescence) to evaluate particle–cell interactions. Both in the presence (Figure S16, Supporting Information) and absence (**Figure** **6**a) of mucus and for all the conditions tested and respective controls, the monolayer retained its integrity, and no cell detachment was observed (Figure S17–S21, Supporting Information).

Incubation with Pitstop 2, mβCD, and OA largely influenced the residual fluorescence of the nanoparticles on the cell monolayer with respect to the negative controls, suggesting modulation of particle–cell interactions (Figure 6b–f). However, these responses were also dependent on particle functionalization. In the presence of mucus, the preincubation with Pitstop 2 did not affect the measured residual fluorescence intensities (*t*_6_/*t*_0_) of **DMSNs**, **D‐CH**_**3**_, and **DPO**_**3**_ **‐Farn** in comparison to controls (Figure S22a–e, Supporting Information; Figure 6b–d). In turn, after mucus removal, Pitstop 2 significantly decreased residual fluorescence intensities of all particle treatments (Figure S22a–e, Supporting Information; Figure 6b–d). The interaction of **D‐PO**_**3**_ with the monolayer was the most affected by the preincubation with Pitstop 2 (Figure 6d and S22b, Supporting Information, ± Mucus). Even in the presence of mucus, only 10% of the initial FITC fluorescence was detected after 6 h treatment with the phosphonated particles (Figure 6d), in contrast to the 26% measured for the respective negative control (Figure 6b, − Pitstop 2). In the absence of mucus, the residual fluorescence of **D‐PO**_**3**_ was reduced from 36% (Figure 6b, − Pitstop 2) to 8% upon preincubation with Pitstop 2 (Figure 6d, + Pitstop 2). A similar, albeit more attenuated response was observed for **D‐Farn** (Figure 6d and S22d, Supporting Information, ± Mucus).

This trend was less visible upon incubation with **DPO**_**3**_ **‐Farn** (Figure 6d and Figure S22e, Supporting Information) despite the presence of both phosphonate and farnesol functions grafted on the surface of the particles. In this case, the residual fluorescence only decreased significantly, i.e., from 27% (Figure 6b, − Pitstop) to 19% (Figure 6d, + Pitstop 2), once the mucus layer was removed (Figure S22e, Supporting Information). For this material, the preincubation with mβCD (**DPO**_**3**_ **‐Farn**; Figure 6e) and OA (**DPO**_**3**_ **‐Farn**; Figure 6f) had a stronger influence on its interaction with the intestinal cells since only 4% of the initial fluorescence (*t*_0_) was detected after 6 h incubation, both in the presence and absence of mucus (Figure S22e, Supporting Information). Despite the significant decrease in the residual fluorescence intensities observed for all the particle treatments in these conditions (+ mβCD/ + OA, Figure S22a–e, Supporting Information), preincubation with mβCD altered more significantly the interaction of **D‐CH**_**3**_ and **DPO**_**3**_ **‐Farn** with the cell monolayer in comparison to the other materials (± Mucus, Figure 6e). A similar response was also observed for **D‐PO**_**3**_, which was more pronounced in the absence of mucus (Figure 6e). The modulation of barrier function with OA also led to significantly lower residual fluorescence intensities with respect to the control conditions (− OA, Figure 6c), with **D‐PO**_**3**_, **D‐CH**_**3,**_ and **DPO**_**3**_ **‐Farn** being the most affected materials (± Mucus, Figure 6f).

After preincubation with Pitstop 2, mβCD, and OA, silica diffusion capacity was also significantly affected both in the presence and absence of mucus (Figure 6g–k). Particularly, the effects of mβCD (Figure 6j) and OA (Figure 6k) were more significant and returned the lowest values of the focal plane adjustments (*t*_0_–*t*_6_, μm) for all the nanoparticle treatments (Figure S22f–j, Supporting Information). This clearly supports the fundamental role of membrane architecture and integrity in the regulation of particle−cell interactions. In this regard, it also aligned with the behavior of **D‐PO**_**3**_ and **DPO**_**3**_ **‐Farn** (i.e., + Mucus; Figure 6h,j,k), which rather differed in the amplitude of the response in the presence of Pitstop 2 (+ Mucus; Figure 6g,i and S22g,j, Supporting Information). Additionally, these results confirmed that despite the efficient interaction observed for the phosphonate‐functionalized particles (**D‐PO**_**3**_ and **DPO**_**3**_ **‐Farn**), both enhancing cell–cell permeability, the mechanism of association with the intestinal barrier differed from one material to another, being chemically modulated by distinct surface properties.

## 3 Conclusion

In summary, phosphonate‐functionalized particles **D‐PO**_**3**_ diffused better through the mucus barrier due to their higher hydrophilicity and negative surface charge. PEG grafting (i.e., **D‐PEG**) had a positive impact on particle transport through the mucus, albeit demonstrating more limited interaction with the intestinal cells. Dual functionality conferred by the combination of phosphonate groups with hydrophobic moieties did not entirely suppress the interactions with the mucins of the mucus layer during particle trafficking. In return, such hybrid surfaces (i.e., **DPO**_**3**_ **‐CH**_**3**_ and **DPO**_**3**_ **‐Farn**) allowed enhanced interaction with the cell membrane, with a more significant influence on paracellular permeation, easing cell separation. The redistribution of TJ proteins triggered by particle treatments highlighted the better performance of the phosphonated materials. Thus, the correlation between the surface properties of MSNs and their activity profile, in close alignment with the modulation of the barrier function, promises to support unprecedented rational design of nanocarriers with differential biological performance. The fundamental approaches covered herein aim to promote a wide range of applications of functionalized silica, including the modulation of transcellular and/or paracellular transports. These potentially span from the development of novel therapies for the treatment of intestinal diseases and protection against pathogens and food contaminants (e.g., via reinforcement of the tight architecture of the intestinal barrier) up to the systemic delivery of oral peptide drugs and biologics.

## 4 Experimental Section

The materials and methods used in this study have been described in detail in the Supporting Information.

## 5 List of Abbreviations

**Table:** 

APTES	3‐(aminopropyl)triethoxysilane
BSA	Bovine serum albumin
CLDN4	Claudin‐4
CP	Cross‐polarization
CTAC	Cetyltrimethylammonium chloride
D‐CH_3_	Methylated‐DMSNs
D‐CH_3_‐FITC	Methylated‐DMSNs labeled with fluorescein isothiocyanate
D‐Epox	Epoxide‐grafted DMSNs
D‐Farn	DMSNs functionalized with farnesol.
D‐Farn‐(1 step)	DMSNs functionalized with a GPTMS‐Farn conjugate (synthetic control)
DLS	Dynamic light scattering
DMSNs	(Non‐functionalized) dendritic mesoporous silica nanoparticles
DMSO	Dimethyl sulfoxide
D‐PEG	PEGylated DMSNs
D‐PO_3_	Phosphonate‐grafted DMSNs
DPO_3_‐CH_3_	Phosphonate‐grafted DMSNs further functionalized with TMS.
DPO_3_‐Epox	Phosphonate‐grafted DMSNs further functionalized with GPTMS.
DPO_3_‐Epox + HCl	Control obtained after the addition of HCl (37%) to phosphonated DMSNs containing GPTMS
DPO_3_‐Farn	Phosphonate‐grafted DMSNs functionalized with farnesol
DPO_3_‐Farn‐(1 step)	Phosphonated DMSNs functionalized with a GPTMS‐Farn conjugate (synthetic control)
Farn	Farnesol
FITC	Fluorescein isothiocyanate
GPTMS	3‐(glycidyloxypropyl)trimethoxysilane
GPTMS‐Farn	Conjugate obtained from the reaction of GPTMS with farnesol in an acidic medium
MAS NMR	Solid‐state magic angle spinning nuclear magnetic resonance spectroscopy
MSNs	Mesoporous silica nanoparticles
MUC5AC	Mucin‐5AC
mβCD	Methyl‐β‐cyclodextrin
NPC	Nuclear pore complex
OA	Oleic acid
PDI	Polydispersity index
PEG	Polyethylene glycol
PSD	Particle size distribution
S_BET_	Specific surface area calculated using the Brunauer–Emmet–Teller equation
TEA	Triethanolamine
TEER	Transepithelial electrical resistance
TEM	Transmission electron microscopy
TEOS	Tetraethyl orthosilicate
TGA	Thermogravimetric analysis
THMP	3‐(trihydroxysilyl)propyl‐methyl‐phosphonate silane
TJs	Tight junction proteins
TMS	Trimethoxymethylsilane
ZO‐1	Zonula occludens‐1

## Conflict of Interest

The authors declare no conflict of interest.

## Supporting information

Supplementary Material

## Data Availability

The data that support the findings of this study are available from the corresponding author upon reasonable request.
