# Supplementary material for: Functionalization of Silica Nanoparticles for Tailored Interactions with Intestinal Cells and Chemical Modulation of Paracellular Permeability
Source: Small Sci. 2024 Aug 1;5(1):2400112. doi: 10.1002/smsc.202400112 (PMC11934983; doi:10.1002/smsc.202400112)
Supplement: Supplementary file 1 — Supplementary Material [file SMSC-5-2400112-s001.pdf]

Functionalization of Silica Nanoparticles for Tailored Interactions with Intestinal Cells and Chemical Modulation of Paracellular Permeability

Claudia Iriarte-Mesa,[a,b] Janice Bergen,[b,c,d] Kristina Danielyan,[a] Francesco Crudo,[d] Doris Marko,[d] Hanspeter Kählig,[e] Giorgia Del Favero,[c,d] Freddy Kleitz[a*]*

***Corresponding Authors:** giorgia.del.favero@univie.ac.at; freddy.kleitz@univie.ac.at

[a] Department of Functional Materials and Catalysis, Faculty of Chemistry, University of Vienna, Währinger Str. 42, 1090 Vienna, Austria

[b] Vienna Doctoral School in Chemistry (DoSChem), University of Vienna, Währinger Str. 42, 1090 Vienna, Austria

[c] Core Facility Multimodal Imaging, Faculty of Chemistry, University of Vienna, Währinger Str. 38-40, 1090 Vienna, Austria

[d] Department of Food Chemistry and Toxicology, Faculty of Chemistry, University of Vienna, Währinger Str. 38-40, 1090 Vienna, Austria

[e] Department of Organic Chemistry, Faculty of Chemistry, University of Vienna, Währinger Str. 38, 1090 Vienna, Austria

TABLE OF CONTENTS

Materials ... 3
Methods ... 3
Synthesis of silica nanoparticles ... 3
Functionalization of silica nanoparticles (DMSNs). ... 4
Labeling of silica nanoparticles ... 6
Cell culture ... 7
Cell treatments. ... 7
Cell Viability: Neutral Red Assay. ... 8
Fluorescence microscopy experiments. ... 8
Immunofluorescence staining and microscopy. ... 10
Transepithelial Electrical Resistance (TEER). ... 11
Paracellular Permeability: Lucifer Yellow ... 12
Characterization of the materials. ... 13
Statistical analysis. ... 14

LIST OF FIGURES

S1. DLS profiles, zeta potential, and colloidal stability of silica nanoparticles ... 15
S2. Mass loss (%) and DSC profiles of functionalized silica nanoparticles ... 16
S3. N_2-sorption isotherms and NLDFT pore size distribution of silica nanoparticles ... 16
S4. Solid-state ^{29}Si CP/MAS NMR of the synthesized materials ... 18

S5. Solid-state ^{13}C CP/MAS NMR spectra of FITC labeled **D-CH$_3$** and synthetic controls **DPO$_3$-Epox+HCl**, **D-Farn-(1 step)**, and **DPO$_3$-Farn-(1 step)** 19

S6. Solid-state ^{31}P MAS NMR spectra of the functionalized DMSNs 21

S7. Cell viability of Caco-2/HT29-MTX-E12 cells treated with silica nanoparticles 23

S8. Phase contrast images of intestinal cells after incubation with silica nanoparticles in complete cell culture medium (10× magnification) 24

S9. Immunofluorescence staining of MUC5AC and quantification of FITC and MUC5AC intensities 25

S10. Z-plot profiles of MUC5AC and quantification of the corresponding relative Z-position and thickness 26

S11. 3D-reconstructions of the immunofluorescence staining of ZO-1 and CLDN4 after interaction of silica particles with intestinal cells 27

S12. Cell permeability of intestinal cells treated with DMSNs (TEER measurements and Lucifer Yellow assay) 28

S13. Z-plot profiles and quantification of the relative Z-position and thickness of TJs (ZO-1 and CLDN4) 29

S14. Immunofluorescence staining of ZO-1 and CLDN4 after the interaction of DMSNs with intestinal cells 30

S15. Quantification of the mean intensity fluorescences (%) of ZO-1 and CLDN4 31

S16. Phase contrast images (10× magnification) of intestinal cells after incubation with Pitstop 2, mβCD and OA in the presence of mucus and subsequent treatments with silica nanoparticles 32

S17. Phase contrast images (10× magnification) of intestinal cells after incubation in serum-free medium (– Pitstop 2, 10 min) followed by treatments with silica nanoparticles 33

S18. Phase contrast images (10× magnification) of intestinal cells after incubation in serum-free medium containing bovine serum albumin (– mβCD/– OA, 20 h) followed by treatments with silica nanoparticles 34

S19. Phase contrast images (10× magnification) of intestinal cells after incubation with Pitstop 2 (25 µM) followed by treatments with silica nanoparticles 35

S20. Phase contrast images (10× magnification) of intestinal cells after incubation with mβCD (50 µM) followed by treatments with silica nanoparticles 36

S21. Phase contrast images (10× magnification) of intestinal cells after incubation with OA (100 µM) followed by treatments with silica nanoparticles 37

S22. Extended comparison among particle treatments in the presence of different chemical modulators. 38

LIST OF SCHEMES

S1. Reaction of phosphonate and glycidyl moieties of **DPO$_3$-Epox** in acid medium 5

S2. Synthesis of the control samples **D-Farn-(1 step)** and **DPO$_3$-(1 step)** 6

S3. Synthetic steps for the labeling of silica nanoparticles 7

S4. Measurements of the appearance of cell-cell distance from live cell imaging 9

LIST OF TABLES

S1. Physico-chemical parameters of the silica-based nanoparticles 17

S2. Assignment of the signals in the solid-state ^{29}Si CP/MAS NMR spectra 18

S3. Assignment of the signals in the solid-state ^{13}C CP/MAS NMR spectra 19

S4. Assignment of the signals in the solid-state ^{31}P MAS NMR spectra 22

REFERENCES 39

Materials

Tetraethylorthosilicate (TEOS, 98%), hexane (≥99%), cetyltrimethylammonium chloride (CTAC, 25 wt% in H_2O), fluorescein isothiocyanate (FITC, ≥90%), (3-glycidyloxypropyl)trimethoxysilane (GPTMS, 98%), 3-(trihydroxysilyl)propyl methylphosphonate (THMP, monosodium salt solution, 50 wt% in H_2O), trimethoxymethylsilane (TMS, ≥98%), Neutral Red dye, oleic acid (OA, O1383-1G), anhydrous toluene (99.8%), and donkey serum (D9663) were purchased from Sigma Aldrich (Taufkirchen, Germany). PEG-silane ($(CH_3O)_3Si$-PEG-OCH_3, M_w 2 kDa) was purchased from Rapp Polymere GmbH (Tuebingen, Germany). Farnesol (96%, mixture of isomers) was obtained from Thermo Fisher Scientific (Waltham, MA, USA). Triethanolamine (TEA, ≥99%), anhydrous ethanol (EtOH, 95%), *N*-acetylcysteine (BioReagent, suitable for cell culture), (3-aminopropyl)triethoxysilane (APTES, 98%), and dimethyl sulfoxide (DMSO, ≥99%) were purchased from Alfa Aesar (Massachusetts, USA). Hydrochloric acid (HCl, 37%) was purchased from Fluorochem (Hadfield, UK). The cell-permeable clathrin inhibitor Pitstop 2 (ab120687) was purchased from Abcam Biochemicals (Cambridge, UK). Methyl-β-cyclodextrin (mβCD) was obtained from Sigma-Aldrich (St. Louis, US). Lucifer Yellow CH di-lithium salt was purchased from Santa Cruz Technologies (Dallas, TX, USA). HEPES buffer, $CaCl_2$, $MgCl_2$, glycine, and Triton X-100 were purchased from Carl Roth (Karlsruhe, Germany). Methanol (MeOH) and glacial acetic acid (HAc) were acquired from Honeywell (Seelze, Germany) and Merck (Darmstadt, Germany), respectively. Primary antibodies for immunofluorescence experiments were obtained from Abcam (Cambridge, U.K.): anti-Zonula occludens-1 (ZO-1, ab190085, goat polyclonal), anti-Claudin-4 (CLDN4, ab53156, rabbit polyclonal), and anti-Mucin 5AC (MUC5AC, ab77576, mouse monoclonal). Mounting medium containing DAPI (ab104139) was also acquired from Abcam (Cambridge, U.K.). The fluorescently labeled secondary antibodies were obtained from Probes-Life Technologies Invitrogen, Thermo Fisher Scientific (Waltham, MA, USA): Alexa Fluor™ 647 donkey anti-goat IgG (H+L, 705-605-003), Alexa Fluor™ 568 donkey anti-rabbit IgG (H+L, A10042), and Alexa Fluor™ 647 donkey anti-mouse IgG (H+L, A31571). Materials for cell culture and fluorescence imaging were purchased from GIBCO Invitrogen (Karlsruhe, Germany), Lonza Group Ltd (Basel, Switzerland), Sigma-Aldrich Chemie GmbH (Munich, Germany), and Sarstedt AG&Co (Nuembrecht, Germany).

Methods

Synthesis of silica nanoparticles. The synthesis of dendritic mesoporous silica nanoparticles (**DMSNs**) was performed following the procedure described by Juère *et al.*[1] with slight modifications. TEA (360 mg), CTAC (8 mL), and 72 mL of deionized water were mixed and stirred (150 rpm) for 1 h at 60 °C. An organic mixture of hexane (32 mL) and TEOS (8 mL) was

then added dropwise. The biphasic system was slowly stirred (150 rpm) at 60 °C overnight. The organic phase was subsequently removed, and the solution was centrifuged for 20 min at 10 000 rpm. After drying at 100 °C overnight, the as-made product was extracted for 2 h with 100 mL of EtOH and 1 drop of HCl (37%) at 90 °C. The nanoparticles were dried overnight at 100 °C and then were calcined at 550 °C for 5 h. Individual batches were synthesized and characterized. After confirming the reproducibility of the synthesis, as well as the retention of size, morphology, and zeta potential of samples obtained from different replicates, the good-quality batches were combined, and the final product (**DMSNs**, 6.3 g) was fully characterized.

Functionalization of silica nanoparticles (DMSNs). Dendritic silica nanoparticles (**DMSNs**) were functionalized with a PEG-silane (2 kDa) *via* a general post-grafting (PG) protocol reported by von Baeckmann *et al.*[2] First, 300 mg of calcined **DMSNs** were degassed overnight at 150 °C. The nanoparticles were then dispersed in 80 mL of anhydrous toluene at 110 °C, under stirring (600 rpm) and argon atmosphere. After 3 h, the PEG-silane (38 mg, 2 kDa) was dissolved in anhydrous toluene under an argon atmosphere and added to the silica dispersion. The reaction was stirred overnight (600 rpm) at 110 °C. The PEGylated-DMSNs (**D-PEG**) were recovered by centrifugation (9 000 x g, 20 min), washed once with toluene, twice with ethanol, and dried at 40 °C overnight.

For the functionalization of DMSNs with phosphonate groups (**D-PO$_3$**), the protocol described by Bouchoucha *et al.*[3] was implemented with some modifications. First, THMP (3.3 mL, 16 mmol·g^{-1} of silica) was dissolved in water (100 mL). The highly basic pH of the solution obtained (pH = 10.9) was brought to 5 with HCl (0.1 M) to avoid silica hydroxylation and dissolution during the grafting reaction. Slightly acidic pH could also catalyze the condensation between silanol groups on the silica surface and silanol groups of the phosphonate silane (THMP).[3] The THMP solution was then added to a dispersion of calcined **DMSNs** (800 mg in 100 mL of water), followed by overnight reflux at 100 °C. The phosphonate-functionalized DMSNs (**D-PO$_3$**) were isolated by centrifugation (9 000 x g, 20 min) and washed once with water and three times with ethanol before overnight drying at 40 °C.

A protocol like the PG method described above was implemented for the functionalization of silica nanoparticles with TMS, rendering the methyl-functionalized particles **D-CH$_3$** or **DPO$_3$-CH$_3$**, which were obtained from **DMSNs** and **D-PO$_3$**, respectively. First, 300 mg of the precursor materials were outgassed overnight at 150 °C (**DMSNs**) or 80 °C (**D-PO$_3$**), and the nanoparticles were then dispersed in anhydrous toluene (80 mL) for 3 h before adding TMS (0.7 mL, 16 mmol·g^{-1} of degassed silica). Both reactions were kept under an argon atmosphere and vigorous stirring (600 rpm) at 110 °C overnight. The functionalized products (*i.e.*, **D-CH$_3$** and **DPO$_3$-CH$_3$**) were collected by centrifugation (9 000 x g, 20 min) and washed once with toluene and three times with ethanol. The samples were kept overnight at 40 °C for drying.

The procedure described by von Baeckmann *et al.*[2] was followed for the grafting of GPTMS. The reaction allowed the introduction of epoxide moieties on the surface of **DMSNs** and **D-PO$_3$**, rendering **D-Epox** and **DPO$_3$-Epox**, respectively. The conditions for the PG reaction were the same as described above for TMS-functionalization. However, after degassing the **DMSNs** and **D-PO$_3$** (300 mg of each material), the nanoparticles were dispersed in toluene (80 mL), and the GPTMS (1 mL, 16 mmol·g^{-1} of degassed silica) was added, keeping the stirring (600 rpm) and argon atmosphere at 80 °C. The epoxide-functionalized materials (*i.e.*, **D-Epox** and **DPO$_3$-Epox**) were collected, washed, and dried as described above for methyl-functionalized samples (*i.e.*, **D-CH$_3$** and **DPO$_3$-CH$_3$**).

Finally, farnesol was conjugated to epoxide-functionalized materials (*i.e.*, **D-Epox** and **DPO$_3$-Epox**) by the acid-catalyzed alkoxylation of terminal epoxide moieties grafted on silica.[4] **D-Epox** and **DPO$_3$-Epox** (200 mg of each epoxide-functionalized material, 1 eq. of grafted GPTMS) were outgassed at 80 °C overnight before dispersing the nanoparticles in anhydrous toluene (40 mL). One drop of HCl (37%) was added to the silica dispersions, followed by the addition of farnesol (0.8 mL, 30 eq.). The reaction was kept overnight under stirring (600 rpm) at 80 °C. The products obtained from **D-Epox** (*i.e.*, **D-Farn**) and **DPO$_3$-Epox** (*i.e.,* **DPO$_3$-Farn**) were collected by centrifugation (9 000 x g, 20 min) and were washed once with toluene and twice with ethanol, before overnight drying at 40 °C.

For control purposes, the same procedure was carried out with 100 mg of **DPO$_3$-Epox** but without adding farnesol after acidification (Scheme S1). The product obtained (**DPO$_3$-Epox+HCl**) was recovered, washed, and dried as described above.

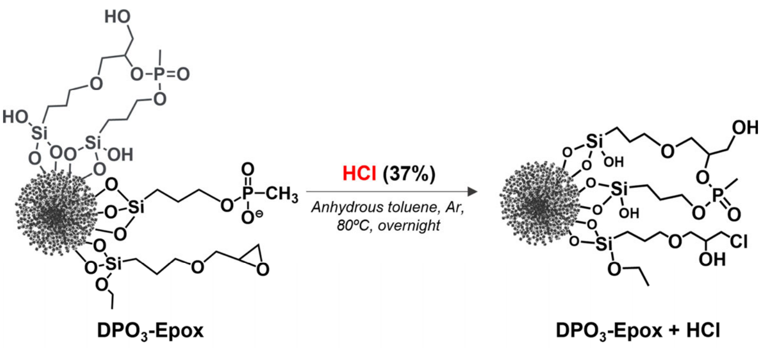

Scheme S1. Reaction of phosphonate and glycidyl moieties of **DPO$_3$-Epox** in an acid medium to obtain the synthetic control **DPO$_3$-Epox+HCl**. The opening of the oxirane ring of GPTMS in the presence of concentrated HCl (37%) rendered a chlorohydrin derivative.[5]

Two additional control samples were prepared from the previous reaction of farnesol with GPTMS in an acid medium. The conjugate obtained (GPTMS-Farn) was then used for the functionalization of calcined (**DMSNs**) and phosphonated particles (**D-PO$_3$**) in one step post-grafting reaction (Scheme S2).

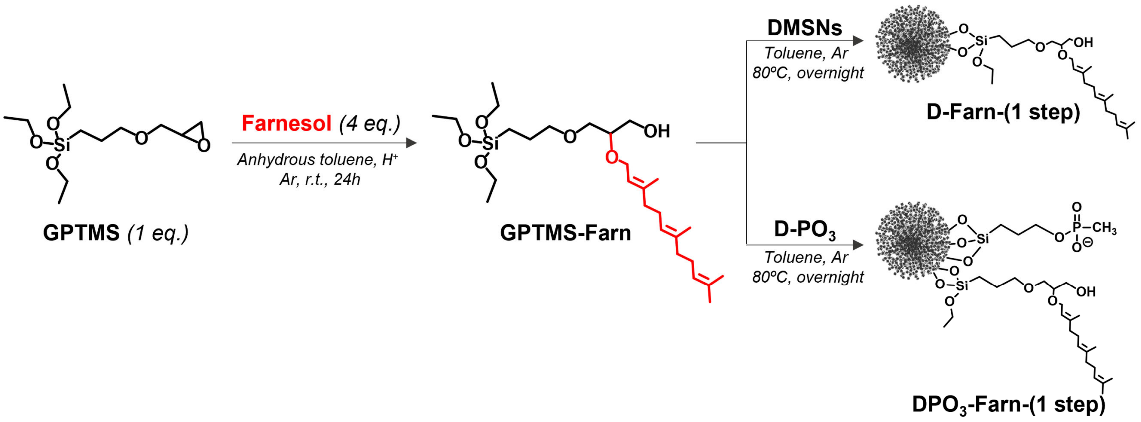

Scheme S2. Synthesis of the control samples **D-Farn-(1 step)** and **DPO₃-(1 step)**.

First, GPTMS (2.6 mL, 1 eq.) was dispersed in anhydrous toluene (6 mL). The mixture was stirred at room temperature (500 rpm), followed by the addition of 2 drops of HCl (37%). After 30 min, farnesol was added (4 mL, 4 eq.), and the reaction was kept overnight at room temperature and under an argon atmosphere. In parallel, 100 mg of **DMSNs** and **DPO$_3$** were degassed overnight at 150 °C or 80 °C, respectively. The nanoparticles were then dispersed in anhydrous toluene (30 mL), and the GPTMS-Farn stock solution (0.37 mol·L^{-1}) was added to silica (4.3 mL, 16 mmol·g^{-1} of degassed silica). The reactions were kept overnight under stirring (600 rpm) at 80 °C. The control samples obtained, *i.e.*, **D-Farn-(1 step)** and **DPO$_3$-(1 step)**, were treated as their respective equivalents **D-Farn** and **DPO$_3$-Farn**.

Labeling of silica nanoparticles. The samples obtained for *in vitro* cell testing (*i.e.*, **DMSNs**, **D-PEG**, **D-PO$_3$**, **D-CH$_3$**, **D-Farn**, **DPO$_3$-CH$_3$**, and **DPO$_3$-Farn**) were labeled with fluorescein isothiocyanate (FITC) based on a previously reported method.[6] First, a FITC-APTES silane was prepared by the covalent coupling of FITC with APTES (Scheme S3). FITC (20 mg, 4 eq.) was dissolved in 10 mL of anhydrous EtOH, and APTES (3 µL, 1 eq.) was added at room temperature under stirring (800 rpm) and argon atmosphere. The reaction was kept for 24 h in the dark while 100 mg of the functionalized silica nanoparticles (*i.e.*, **D-PEG**, **D-PO$_3$**, **D-CH$_3$**, **D-Farn**, **DPO$_3$-CH$_3$**, and **DPO$_3$-Farn**) were degassed overnight at 80 °C and dispersed in 30 mL of anhydrous toluene at 50 °C under stirring (700 rpm). The same procedure was followed for calcined **DMSNs**, but the temperature for degassing was increased up to 150 °C. Subsequently, the FITC-APTES stock solution (100 µL) was added to each dispersion of functionalized particles, and the grafting was further carried out overnight. For the labeling of **DMSNs**, 50 µL of the FITC-APTES conjugate was added. The FITC-labeled silica nanoparticles were recovered by centrifugation (9 000 rpm, 20 min), washed once with toluene and three times with ethanol, and then dried at 35 °C for 24 h. The procedure implemented rendered comparable amounts of grafted FITC after the labeling reactions (*i.e.*, 1-4 wt% according to TGA).

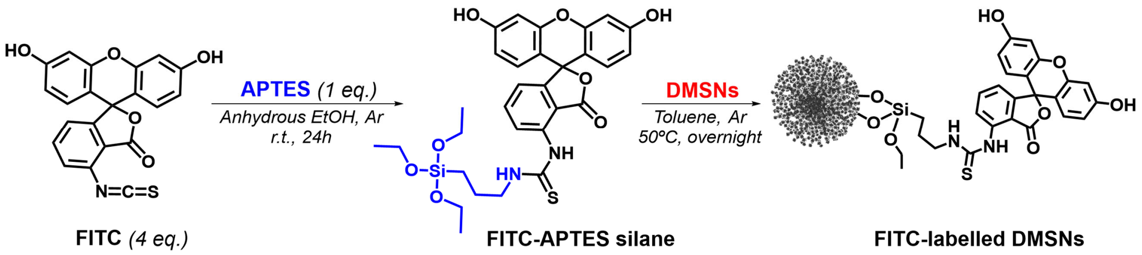

Scheme S3. Synthetic steps for the labeling of the silica nanoparticles.

Cell culture. Caco-2 cells and HT29-MTX-E12 were purchased from ATCC and cultivated in DMEM (high glucose, 4.5 g·L^{-1}) supplemented with fetal bovine serum (FBS, 10%), L-glutamine (1%, 2 mM), non-essential amino acids (1%), and penicillin-streptomycin (1%), according to the specification of the supplier. The cells were incubated in a humidified incubator with 5% CO_2 at 37 °C. For cell viability and live cell imaging experiments, a total cell density of 85 000 cells·cm^{-2} was seeded in 24 wells-cell culture plates (Sarstedt, 1.82 cm^2 growth area) using 500 µL of cell culture medium, which rendered approx. 15 4700 cells/well (139 230 Caco-2 cells and 15 470 HT29-MTX-E12 cells).[7] The Caco-2/HT29-MTX-E12 co-culture was incubated at 37 °C for 7 days, replacing the cell culture medium with fresh medium every two days. At the end of the differentiation time, a homogenous layer of mucus was visible on the cell co-culture.

Cell treatments. After 7 days of differentiation, the medium was removed, the cells were washed twice with Dulbecco's phosphate-buffered saline (DPBS), and 500 µL of freshly prepared dispersions of FITC-labeled silica nanoparticles (*i.e.*, **DMSNs**, **D-PEG**, **D-PO$_3$**, **D-CH$_3$**, **D-Farn**, **DPO$_3$-CH$_3$**, and **DPO$_3$-Farn**) were added. The particle suspensions were prepared in a complete culture medium (600 µg·mL^{-1}) according to a standard operating procedure (SOP). The suspensions were vortexed for 15 secs and placed in the ultrasonic bath for 20 min prior to cell treatment to ensure stable and homogenous dispersions. The cells were then incubated with the silica nanoparticles for 6 h at 37 °C.

For the experiments involving the inhibition of clathrin receptors, the inhibitor Pitstop 2 (8.25 mM in DMSO) was diluted in a serum-free medium (25 µM, 0.3% DMSO). This solution was added to the cells (500 µL/well) prior to particle treatment, followed by 10 min-incubation at 37 °C according to the specification of the supplier (+ Pitstop 2). For the negative control (– Pitstop 2), 500 µL of serum-free medium (+ 0.3% DMSO) was used for the incubation. The cells were washed twice with serum-free medium and then incubated with the FITC-labeled silica nanoparticles (*i.e.*, **DMSNs**, **D-PO$_3$**, **D-CH$_3$**, **D-Farn**, and **DPO$_3$-Farn**) for 6 h at 37 °C, which were previously dispersed in serum-free medium (600 µg·mL^{-1}).

For the experiments based on the biochemical modulation of the cell membrane (*i.e.*, mβCD and OA treatments), the cell medium was removed after 7 days of differentiation, and the cells

were washed twice with DPBS. Solutions of mβCD (50 μM, 0.05% DMSO) or OA (100 μM, 0.03% DMSO) were freshly prepared in serum-free medium containing bovine serum albumin (+ BSA, 1 mg·mL^{-1}) and added to the cell wells (500 μL/well), followed by 20 h-incubation at 37 °C. For the negative controls (– mβCD/– OA), 500 μL of serum-free medium (+ BSA, 1 mg·mL^{-1}), containing 0.05% DMSO, were used for the 20 h-incubation. The cells were then washed twice with serum-free medium, and the treatments with FITC-labeled nanoparticles (*i.e.*, **DMSNs**, **D-PO$_3$**, **D-CH$_3$**, **D-Farn**, and **DPO$_3$-Farn**) were applied in serum-free medium (600 μg·mL^{-1}), following the same procedure described above.

For the experiments performed in the absence of mucus (– Mucus), the procedure described by Behrens *et al.* [8] was implemented for mucus removal. The cells were first washed with DPBS (550 μL/well), and *N*-acetylcysteine dissolved in serum-free medium (10 mM, 500 μL/well) was added. After 1 h-incubation at 37 °C under agitation (220 rpm), the cells were washed twice with DPBS and treated according to the protocol described above for the evaluation of particle-cell interactions in the presence of the mucus layer and pre-incubation with Pitstop 2, mβCD or OA.

Cell Viability: Neutral Red Assay. The Neutral Red Assay was performed as previously described.[9–11] After 7 days of differentiation, the cells were treated for 6 h with non-fluorescent particles (*i.e.*, **DMSNs**, **D-PEG**, **D-PO$_3$**, **D-CH$_3$**, **D-Farn**, **DPO$_3$-CH$_3$**, and **DPO$_3$-Farn**), previously dispersed in complete cell culture medium (600 μg·mL^{-1}). For the measurement of the cell viability in the absence of mucus, the mucus layer was previously removed according to the protocol described above. The assay was carried out in biological triplicate, including technical duplicates for each condition. The experimental layouts included solvent and negative controls (*i.e.*, non-treated cells). Neutral Red dye was dissolved in DPBS to reach a stock concentration of 4 mg·mL^{-1}. The day before use, the Neutral Red stock solution was further diluted to 40 μg·mL^{-1} in cell culture medium (NR medium) and incubated at 37 °C. To remove undissolved dye crystals, NR medium was centrifuged for 10 min at 600 × *g* and subsequently filtered with filter paper. After 6 h-incubation with the nanoparticles, the cells were washed twice with DPBS (550 μL/well), and the medium was replaced by NR medium (500 μL/well), followed by 3 h incubation (37 °C) and subsequent washing with DPBS (550 μL/well) to remove unbound dye. Cells were then treated with 500 μL of de-staining solution (50:50:1 ethanol absolute/H_2O/glacial acetic acid) and shaken for 10 min at 500 rpm on a plate shaker. After transferring 130 μL of de-staining solution to a fresh 96-well plate in triplicate, absorbance was measured at 540 nm using a Cytation3 imaging reader (BioTek, Winooski, VT, USA). Results were related to the respective solvent control (test/control [%]), and data groups were compared with the Student's *t*-test, accepting $p < 0.05$ as the threshold value.

Fluorescence microscopy experiments. Live cell imaging experiments were performed after differentiation protocol, followed by mucus removal (when required), pharmacological

treatments (Pitstop 2, mβCD, and OA), and application of FITC-labeled particles. Imaging of the cells treated immediately after the addition of the particle suspensions (t_0) and after 6 h-incubation at 37 °C (t_6) was performed on a Lionheart FX Automated microscope from BioTek (Vermont, USA). At the end of the 6 h-treatments and before imaging, the cells were washed twice DPBS (550 µL/well) to remove non-internalized FITC-labeled particles before the addition of Cell Imaging Solution (LCI, 500 µL/well). Image acquisition and quantification were performed using GEN5 Microplate Reader and Imager Software Version 3.05 from BioTek (Vermont, USA). The images were composed of the phase contrast and GFP [469, 525 nm] channels. All experiments were carried out using at least three independent cell preparations (biological triplicates). Quantification of the focal plane adjustment at 10× magnification was used to corroborate that particles were able to penetrate through the mucus layer, which was calculated from the difference between the focus value set immediately after FITC-labeled particles-treatment (t_0) and after 6 h-incubation (t_6) at the exact coordinates (*i.e.*, t_0-t_6). At least 18 paired optical fields were analyzed in three independent biological replicates. The mean FITC-fluorescence intensity of the optical fields (10× magnification) acquired at t_0 and t_6 was additionally quantified (GFP [469, 525 nm] channel). Thus, the residual fluorescences were calculated by analyzing the paired images at the exact coordinates ([(t_6)/(t_0)]%). To determine the appearance of cell-cell distances, at least 6 phase contrast-fluorescence images (20× magnification) corresponding to three independent biological replicates were analyzed in ImageJ 1.54i software.[7] A minimum of 10 measurements were performed in each phase contrast image ($n \geq 60$). The separation between the cells (Scheme S4, brighter areas) was determined using the boundaries of the cells defined by the cell membranes as a reference, which related to the areas of darker contrast. Data groups were compared with the one-way ANOVA and Fisher Tests, accepting $p < 0.05$ as the threshold value.

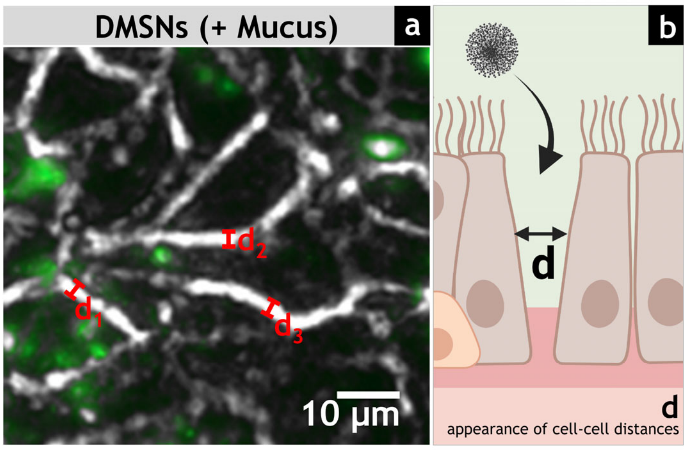

Scheme S4. (a) Graphical representation of three different measurements of cell-cell distances obtained after 6 h of incubation with DMSNs in the presence of mucus (20× magnification). Scale bars stand for 10 µm. (b) Schematic representation of cell accommodation upon particle treatment created with BioRender.com.

Immunofluorescence staining and microscopy. For immunofluorescence experiments, a total cell density of 85 000 cells·cm^{-2} was seeded in 8 well IbiTreat µ-slides (REF: 80.826, Ibidi GmbH, Gräfelfng, Germany) using 200 µL of cell culture medium, which rendered approx. 85 000 cell/well (*i.e.*, 76 500 Caco-2 cells and 8 500 HT29-MTX-E12 cells). After 7-days differentiation, the medium was removed, and the cells were washed twice with DPBS before the application of 200 µL of FITC-labeled nanoparticle dispersions (*i.e.*, **DMSNs**, **D-PEG**, **D-PO$_3$**, **D-CH$_3$**, **D-Farn**, **DPO$_3$-CH$_3$**, and **DPO$_3$-Farn**), freshly prepared in complete culture medium (600 µg·mL^{-1}). For experiments performed in the absence of mucus, the mucus layer was removed before particle treatment using an *N*-acetylcysteine solution in serum-free medium (10 mM, 200 µL/well) and according to the protocol previously described.

Immunofluorescence experiments were performed to stain the tight junction proteins Zonula occludens-1 (ZO-1) and Claudin-4 (CLDN4).[12,13] After 6 h-treatment with silica nanoparticles, the cells were washed twice with DPBS (250 µL/well) and were then fixed by applying MeOH/HAc (90:10), followed by overnight incubation at 4 °C. Thereafter, cells were permeabilized with 0.2% Triton X-100 in phosphate-buffered saline (PBS-A) for 15 min at room temperature. Non-specific binding sites were blocked with 2% donkey serum for 1 h at room temperature. Cells were then incubated overnight at 4 °C with the primary antibodies anti-ZO-1 (dil. 1:500 PBS-A) and anti-CLDN4 (dil. 1:500 PBS-A). Primary antibodies were removed by three consecutive washings with 0.05% Triton X-100 (washing buffer) for 10 min each. After two additional washing steps with PBS-A for 5 min each, the secondary antibodies Alexa Fluor™ 647 donkey anti-goat IgG (H+L) (dil. 1:1000 PBS-A) and Alexa Fluor™ 568 donkey anti-rabbit IgG (H+L) (dil. 1:1000 PBS-A) were applied, and the cells were then incubated in the dark at room temperature for 1.5 h. After three washing steps with 0.05% Triton X-100 and 2 washing steps with PBS-A, cells were incubated with MeOH/HAc (90:10) for 15 min at room temperature as a post-fixation step. The cells were subsequently washed with PBS-A, and the fixative was quenched with glycine (100 mM in PBS-A). Thereafter, the cells were embedded in a mounting medium containing DAPI and kept at 4 °C until imaging.

The same procedure described above was applied for the staining of the mucus protein Mucin 5AC (MUC5AC) after 6h-treatment with FITC-labeled nanoparticles (*i.e.*, **DMSNs**, **D-PO$_3$**, and **DPO$_3$-Farn**), with or without previous application of *N*-acetylcysteine (± Mucus). After particle treatment, fixation, permeabilization, and blocking steps, the cells were incubated overnight at 4 °C with the anti-Mucin 5AC primary antibody (dil. 1:300 PBS-A) according to a previously reported protocol.[13] After washings to remove unbounded anti-Mucin 5AC, the secondary antibody Alexa Fluor™ 647 donkey anti-mouse IgG (H+L) (A31571, dil. 1:1000 PBS-A) was applied and incubated in the dark at room temperature for 1.5 h. The cells were washed with the washing buffer as described above, incubated with the fixative for 15 min, and treated with glycine (100 mM in PBS-A) before adding the mounting medium containing DAPI.

A Zeiss LSM710 laser scanning confocal microscope (ELYRA PS.1 system) equipped with a 63X/1.4 plan-apochromatic oil immersion objective (Zeiss Microscopy GmbH, Germany) was used for imaging. For all the conditions (± Mucus) and particle treatments tested (*i.e.*, **DMSNs**, **D-PEG**, **D-PO$_3$**, **D-CH$_3$**, **D-Farn**, **DPO$_3$-CH$_3$**, and **DPO$_3$-Farn**), at least 3 images were acquired for each of the biological triplicates tested (n = 9), which were composed by the ZO-1, CLDN4, FITC and DAPI channels when the tight junction proteins were stained. Z-stack imaging was additionally performed, obtaining three 3D reconstructions from each replicate (n = 9). For the Mucin 5AC staining, only z-stack imaging was performed for all treatments tested (*i.e.*, **DMSNs**, **D-PO$_3$**, and **DPO$_3$-Farn**), with and without mucus. The 3D reconstructions acquired from the biological triplicates prepared (n = 9) were composed of the MUC5AC, FITC, and DAPI channels.

The Image analysis was performed with ImageJ. The XY-thickness values of the ZO-1 and CLDN4 stainings were measured using the cell nuclei staining (DAPI) as a reference for the selection (XY-thickness, µm). From those linear selections, the mean fluorescence intensities of both ZO-1 and CLDN4 stainings were measured within the regions of interest (ROIs). Each dataset resulted from the analysis of n ≥ 60 cells from three independent preparations (biological triplicates). ZO-1, CLDN4, MUC5AC, and FITC-fluorescence signals were also quantified from the maximum intensity projection of the respective channels in each 3D reconstruction obtained from z-stack imaging (n = 9). To distinguish the contribution of the labeled particles from background autofluorescence, the signal from the FITC channel was acquired for non-treated cells (control) and included in the graphs for comparison.

For each reconstruction, Z-plot profiles of the ZO-1, CLDN4, MUC5AC, and DAPI stainings were obtained. From the respective cross-sectional intensity profiles (n = 9), the maximum intensity was acquired, together with the respective Z-thickness (µm) of the corresponding stainings after thresholding (*i.e.*, Z-distance range covered by fluorescence intensities detected above the mean threshold chosen). For ZO-1 and CLDN4, the mean intensity thresholds were set at 5 and 10, respectively. For MUC5AC, two different intensity threshold values were chosen for the calculation of the total Z-thickness of the mucus layer (*i.e.,* threshold = 5), as well as for the Z-thickness of the Mucin 5AC observed in the apical region (*i.e.,* threshold = 20). The Z-distance (µm) corresponding to the maximum intensity of each cross-sectional intensity profile (Z_{max}) was used to calculate the relative Z-position (µm) of ZO-1, CLDN4, and MUC5AC stainings, taking the nuclei position (*i.e.*, Z-distance of the maximum DAPI-mean intensity, Z_{DAPI}) as reference (*i.e.*, Z_{max} - Z_{DAPI}).

Transepithelial Electrical Resistance (TEER). TEER measurements were carried out to assess the integrity of the cell monolayer as described in previous protocols.[13,14] A total cell density of 85 000 cells·cm^{-2} was seeded in Sarstedt TC inserts for 12 well-plates (Ref. 833931041, PET

membrane, pore size 0.4 µm, 1.1 cm^2 growth area). Complete cell culture medium (1.5 mL) was placed on the basolateral side, while 500 µL of the cell suspension in complete cell culture medium was added to the apical side. This rendered approx. 93 500 cells/well (*i.e.*, 84 150 Caco-2 cells and 9 350 HT29-MTX-E12 cells). After 7-days differentiation, the cell medium was removed, and the co-culture was treated with 500 µL of freshly prepared dispersions of non-fluorescent nanoparticles (*i.e.*, **DMSNs**, **D-PEG**, **D-PO$_3$**, **D-CH$_3$**, **D-Farn**, **DPO$_3$-CH$_3$**, and **DPO$_3$-Farn**), which were added to the apical side (600 $\mu g \cdot mL^{-1}$). The medium of the basolateral side was replaced by a fresh, complete cell culture medium. TEER was measured before and after the treatment with the nanoparticles and subsequent incubation (37 °C) for 6 h and 24 h, using an Epithelial Voltohmmeter (EVOM2) coupled to a chopstick electrode pair (STX2, both World Precision Instruments, Sarasota, FL, USA). Prior to measurements, the chopstick electrodes were rinsed with ethanol and equilibrated in a complete medium at room temperature for 20 min.[13] The experiments were carried out using three independent cell preparations (biological triplicates), including technical duplicates.

TEER measurements were also performed in the absence of mucus (biological triplicates). After 7 days-incubation, the mucus layer was removed as described above. TEER was measured before and immediately after mucus removal. The cells were then treated with **DPO$_3$-Farn**, and the TEER was followed over time (*i.e.*, after 0.5, 1, 2, 3, 4, 5, 6, 7, and 24 h-incubation) at 37 °C. Controls (*i.e.*, non-treated cells) were included in the experimental layout for both conditions tested (± Mucus). The mean value of the three measurements assessed per well was calculated, and the TEER of the blank well was subtracted from the treated wells. All samples' values were presented as percentages of the respective wells before particle treatment (*i.e.*, non-treated cells).

Paracellular Permeability: Lucifer Yellow. After 24 h-incubation with **DMSNs**, **D-PEG**, **D-PO$_3$**, **D-CH$_3$**, **D-Farn**, **DPO$_3$-CH$_3$**, and **DPO$_3$-Farn**, the hydrophilic compound Lucifer Yellow (LY) was used to evaluate the tightness of the monolayers and study changes in paracellular permeability of treated cells.[13] For the assay, both compartments of the transwell system were washed with Hank's balanced salt solution (HBSS) buffer containing 25 mM D-glucose, 20 mM HEPES, 1.25 mM $CaCl_2$ and 0.5 mM $MgCl_2$ at pH 7.4. Subsequently, 0.5 mL of a 0.1 $mg \cdot mL^{-1}$ Lucifer Yellow CH di-lithium salt solution in HBSS buffer (0.1 $mg \cdot mL^{-1}$) and 1.5 mL of pure HBSS buffer were added to the apical and basolateral compartments, respectively. After a 1 h incubation at 37 °C, the fluorescence of the basolateral medium of all wells, as well as pure LY solution, were measured in triplicate (excitation: 485 nm, emission: 535 nm). The assay was additionally carried out in three independent preparations (biological triplicates) after mucus removal and subsequent incubation of the cells with **DPO$_3$-Farn** (600 $\mu g \cdot mL^{-1}$) for 24 h at 37 °C. Control wells (*i.e.*, non-treated cells) were included in the experimental layout for all the

conditions tested (± Mucus). The fluorescence of the sample wells was related to pure LY after subtraction of the blank (HBSS) and was reported as % permeability.

Characterization of the materials. Transmission electron microscopy (TEM) images were collected with a Philips CM200 microscope at an accelerating voltage of 200 kV. The samples for TEM imaging were prepared by dropping EtOH (4 µL) containing the suspended powder sample on a holey carbon film-coated 300 mesh copper grid. Dynamic light scattering (DLS) analyses and zeta potential measurements were performed on a Malvern DTS Nano Zetasizer at a 173° scattering angle (equilibrium time set at 3 min, 3 measurements for each sample). To ensure correct calibration before zeta potential measurements, a standard suspension (carboxylate-modified polystyrene latex microspheres) with a zeta potential of −40 (± 6) mV was measured. The samples were dispersed in H_2O at a concentration of 0.7 $mg{\cdot}mL^{-1}$, vortexed (10 min), and sonicated (90 min) prior to the analysis. The hydrodynamic diameter and zeta potential of the colloidal dispersions were measured over time at different pH values (pH range: 2-10). N_2-physisorption isotherms were measured at -196 °C (77 K) using an Autosorb-iQ3 sorption analyzer (Anton Paar, Boynton Beach, USA). Prior to the analysis, calcined **DMSNs** were outgassed 10 h at 150 °C, while the functionalized nanoparticles (**D-PEG**, **D-PO$_3$**, **D-CH$_3$**, **D-Farn**, **DPO$_3$-CH$_3$**, and **DPO$_3$-Farn**) were outgassed at 80 °C. The specific surface area (S_{BET}) was determined using the Brunauer-Emmet-Teller (BET) equation[15] in the relative pressure range of 0.05 - 0.3 P/P_o. The total pore volume was determined at $P/P_0 = 0.95$. The pore size distributions (PSD) were estimated using the non-local density functional theory (NLDFT) method on the equilibrium branch, considering an amorphous SiO_2 (oxide) surface and a cylindrical pore model. The calculations were carried out using the ASiQwin 5.2 software provided by Anton Paar Quantatech Inc.[16] Thermogravimetric (TGA) and differential scanning calorimetry (DSC) analyses were executed using a Netzsch STA-449 F3 Jupiter instrument from 25 to 800 °C under airflow of 20 $mL{\cdot}min^{-1}$ as carrier gas with a heating rate of 10 $°C{\cdot}min^{-1}$. The mass losses (%) were estimated in the temperature range from 150 to 700 °C. Solid-state magic angle spinning nuclear magnetic resonance spectroscopy (MAS NMR) was performed on a Bruker Avance NEO 500 wide bore system (Bruker BioSpin, Rheinstetten, Germany) at room temperature. A 4 mm triple resonance magic angle spinning (MAS) probe was used. Cross-polarization (CP) was employed using a ramped contact pulse, thereby sweeping the proton radio frequency field from 50 to 100%. For ^{29}Si NMR, the resonance frequency was 99.38 MHz, the MAS spinning speed was 8 kHz, and the CP contact time was 5 ms. The resonance frequency for ^{13}C NMR was 125.78 MHz, the MAS rotor spinning was set to 14 kHz, and the CP contact time to 3 ms. ^{31}P NMR experiments were performed at a frequency of 202.49 MHz with a MAS spinning speed of 14 kHz and a CP contact time of 3 ms. Chemical shifts (δ) were reported in ppm and externally referenced for ^{13}C to adamantane by setting its low field signal to 38.48 ppm,

for ^{29}Si to sodium trimethylsilylpropane sulfonate (DSS) by setting the signal to 0 ppm, and for ^{31}P to $NH_4H_2PO_4$ by setting the signal to 0 ppm.

Statistical analysis. The statistical evaluation and generation of all graphs were performed using OriginPro, version 2019 (OriginLab Corporation, Northampton, MA, USA). One-way ANOVA and Fisher tests were applied to compare treatment groups in fluorescence microscopy experiments. Data was presented in box overlaps, with additional displays of mean values for each distribution. For data comparison corresponding to TEER measurements, as well as Neutral Red and Lucifer Yellow assays, the Student's *t*-test was used instead, and data were presented as mean ± standard deviations. All experiments were carried out using at least three biological replicates. The sample size (*n*), p-values, and inclusion of technical duplicates in each experimental workflow were specified in the method description and corresponding figure captions. In all cases, p-values smaller than 0.05 were considered statistically significant.

Figures and Tables

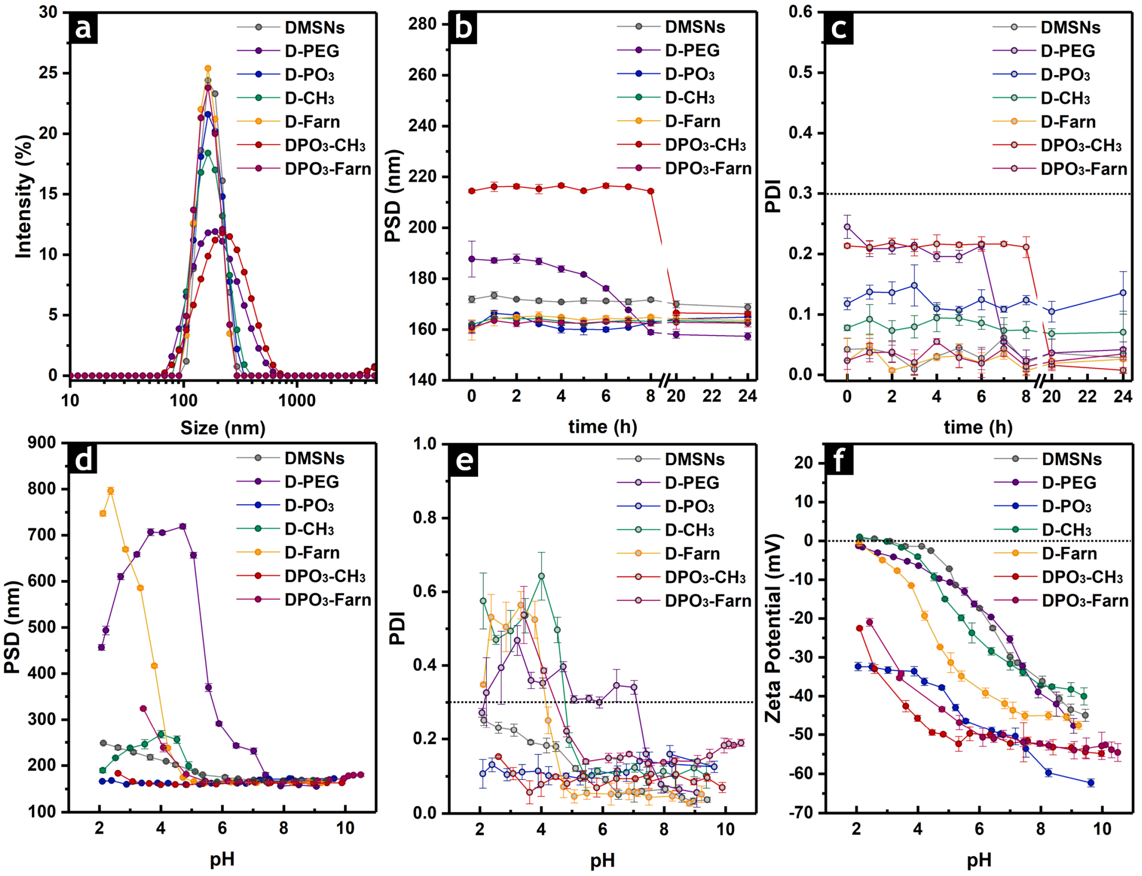

Figure S1. (a) DLS profiles of the silica nanoparticles, *i.e.*, **DMSNs**, **D-PEG**, **D-PO$_3$**, **D-CH$_3$**, **D-Farn**, **DPO$_3$-CH$_3$**, and **DPO$_3$-Farn**, dispersed in H_2O (0.7 mg·mL^{-1}). (b) Particle size distribution (PSD) and (c) respective polydispersity indexes (PDI) tested *via* DLS analysis. (d) pH-dependent PSD values and (e) respective PDI values tested *via* DLS (from pH 2 to 10). (f) Zeta potential of the silica nanoparticles measured over the pH range of 2-10.

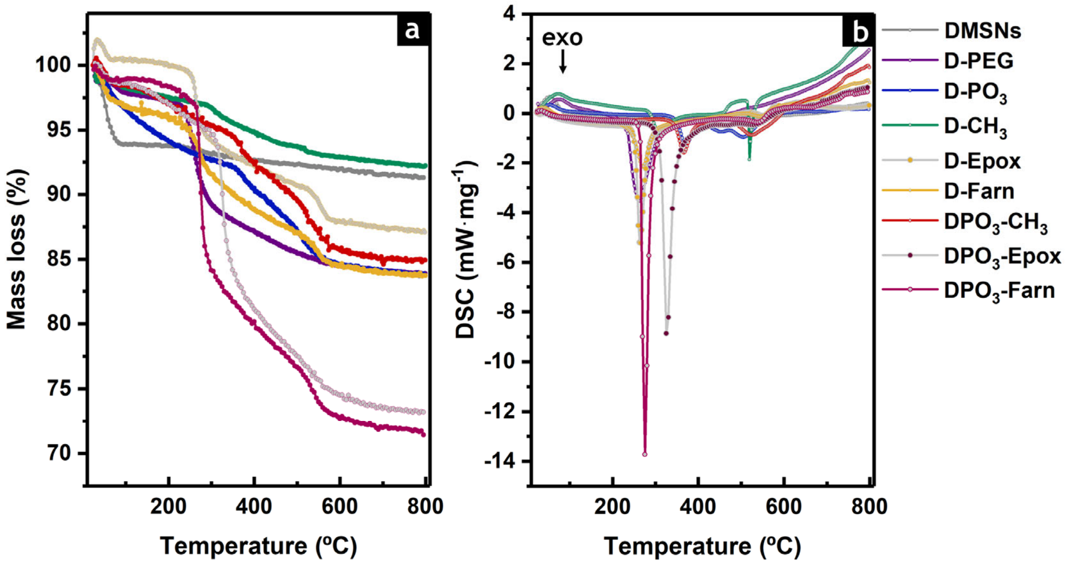

Figure S2. (a) Mass loss (%) and (b) DSC profiles of the different materials obtained, *i.e.*, **DMSNs**, **D-PEG**, **D-PO$_3$**, **D-CH$_3$**, **D-Farn**, **DPO$_3$-CH$_3$**, and **DPO$_3$-Farn**.

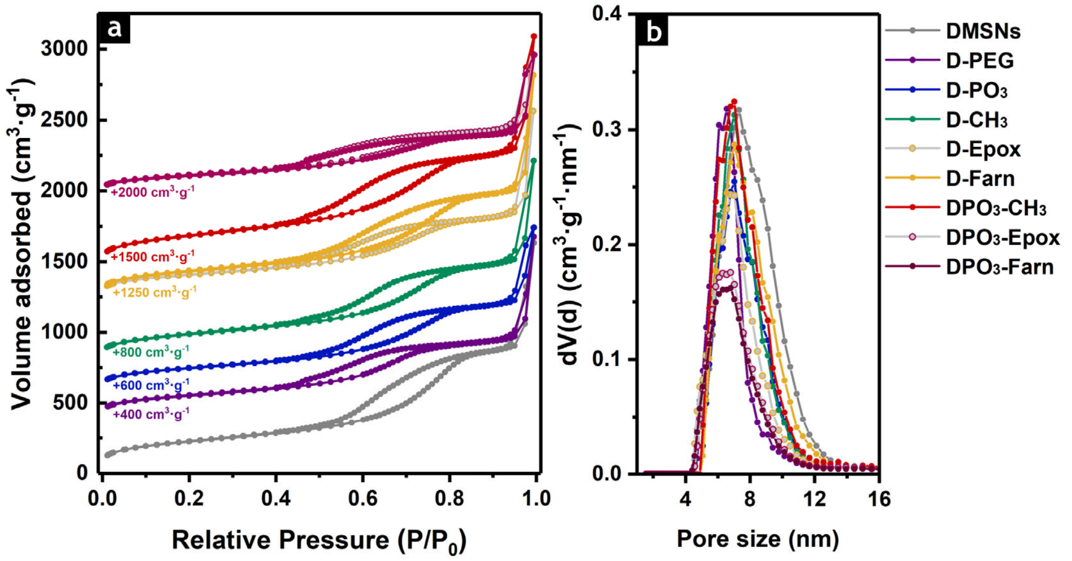

Figure S3. (a) N_2-physisorption isotherms measured at –196 °C (77 K), and (b) respective NLDFT pore size distributions of the materials obtained, *i.e.*, **DMSNs**, **D-PEG**, **D-PO$_3$**, **D-CH$_3$**, **D-Farn**, **DPO$_3$-CH$_3$**, and **DPO$_3$-Farn**, including the precursors **D-Epox** and **D PO$_3$-Epox**, whose isotherms overlap with **D-Farn** and **DPO$_3$-Farn**, respectively.

Table S1. Physico-chemical parameters of the different silica-based nanoparticles obtained.

Material	S_{BET}[a] ($m^2 \cdot g^{-1}$)	Pore size[a] (nm)	Pore volume[a] ($cm^3 \cdot g^{-1}$)	Particle size[b] (nm)	Zeta potential (mV)	Mass loss[c] (%)	Mass loss[g] (FITC,%)
**DMSNs**	808	7.3	2.03	162 (± 1)	-34 (± 1)	-	+ 3.0
**D-PEG**	560	6.6	1.48	217 (± 7)	-25 (± 2)	12.1	+ 2.9
**D-PO_3**	697	7.0	1.95	172 (± 1)	-49 (± 2)	7.6	+ 2.7
**D-CH_3**	675	7.0	1.72	164 (± 1)	-32 (± 2)	6.7	+ 2.4
**D-Epox**	664	7.0	1.87	170 (± 3)	-31 (± 3)	11.4	-
**D-Farn**	572	6.8	1.56	173 (± 3)	-29 (± 2)	+ 2.4[d]	+ 1.8
**DPO_3-CH_3**	541	7.0	1.46	214 (± 1)	-35 (± 2)	+ 2.1[e]	+ 3.4
**DPO_3-Epox**	422	6.8	1.17	178 (± 1)	-40 (± 2)	+ 13.3[e]	-
**DPO_3-Farn**	398	6.8	1.08	178 (± 3)	-38 (± 3)	+ 2.4[f]	+ 1.6

[a] Porosity and textural properties (*i.e.*, specific surface area S_{BET}, pore volume, and pore size) were obtained from the N_2-physisorption analysis (77 K). [b] The particle size (hydrodynamic diameter) was measured using DLS. [c] The mass losses (%) were obtained from TGA and were estimated in the temperature range from 150 to 700 °C. The increases in mass losses after consecutive synthetic steps (+ wt%) were calculated using the mass loss (%) of the respective precursors as reference: [d] **D-Epox**, [e] **D-PO_3**, and [f] **DPO_3-Epox**. [g] Increased mass losses (%) obtained from TGA after the labeling of the silica nanoparticles, corresponding to the fluorescent moieties (+ wt%) grafted.

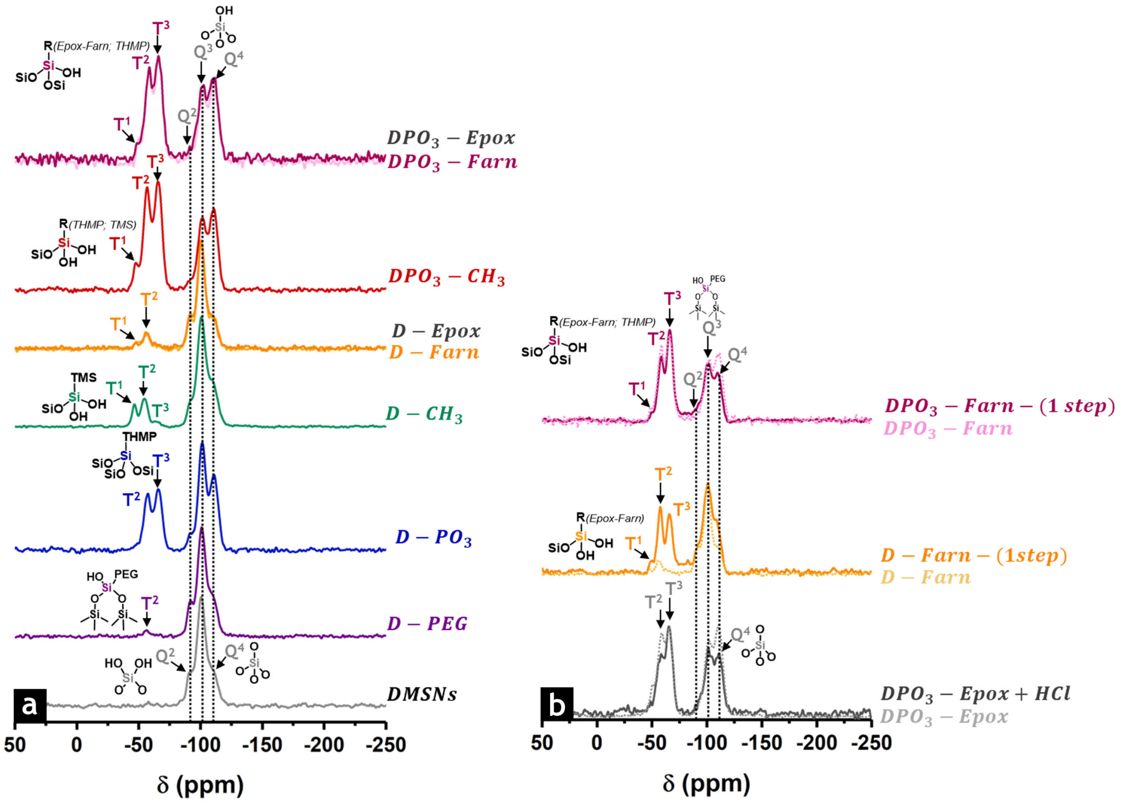

Figure S4. (a) Solid-state ^{29}Si CP/MAS NMR spectra of the different materials obtained (*i.e.*, **DMSNs**, **D-PEG**, **D-PO3**, **D-CH3**, **D-Epox**, **D-Farn**, **DPO3-CH3**, **D PO3-Epox**, and **DPO3-Farn**), as well as (b) synthetic controls, *i.e.*, **DPO3-Epox+HCl**, **D-Farn-(1 step)**, and **DPO3-Farn-(1 step)**. The spectra of the respective equivalent samples (*i.e.*, **D-Epox**, **D-Farn**, and **DPO3-Farn**) were overlapped (lighter lines) for comparison.

Table S2. Assignment of the signals of the solid-state ^{29}Si CP/MAS NMR spectra.

**Signal**[17,18]	**δ (ppm)**					
	**T^1** (≡SiO)Si(OH)$_2$R	**T^2** (≡SiO)$_2$Si(OH)R	**T^3** (≡SiO)$_3$SiR	**Q^2** Si(OH)$_2$(O–)$_2$	**Q^3** Si(OH)(O–)$_3$	**Q^4** Si(O–)$_4$
**DMSNs**	-	-	-	-90.8	-100.5	-109.0
**D-PEG**	-	-56.3	-	-91.3	-100.6	-110.1
**D-PO3**	-	-57.6	-65.6	-91.5	-101.2	-110.7
**D-CH3**	-46.3	-54.8	-65.2	-91.7	-101.8	-110.1
**D-Epox**	-48.8	-56.4	-	-90.1	-100.2	-108.9
**D-Farn**	-47.3	-55.4	-	-92.0	-100.7	-110.4
**DPO3-CH3**	-47.4	-56.9	-65.4	-92.7	-101.4	-111.0
**DPO3-Epox**	-50.8	-58.7	-66.1	-91.4	-101.7	-110.3
**DPO3-Farn**	-48.7	-58.8	-66.2	-	-102.5	-111.3
**D-Farn-(1 step)**	-49.1	-57.9	-65.8	-91.7	-101.2	-108.8
**DPO3-Farn-(1 step)**	-49.1	-58.5	-66.3	-91.7	-101.0	-110.0
**DPO3-Epox + HCl**	-	-58.6	-65.4	-92.3	-101.6	-110.6

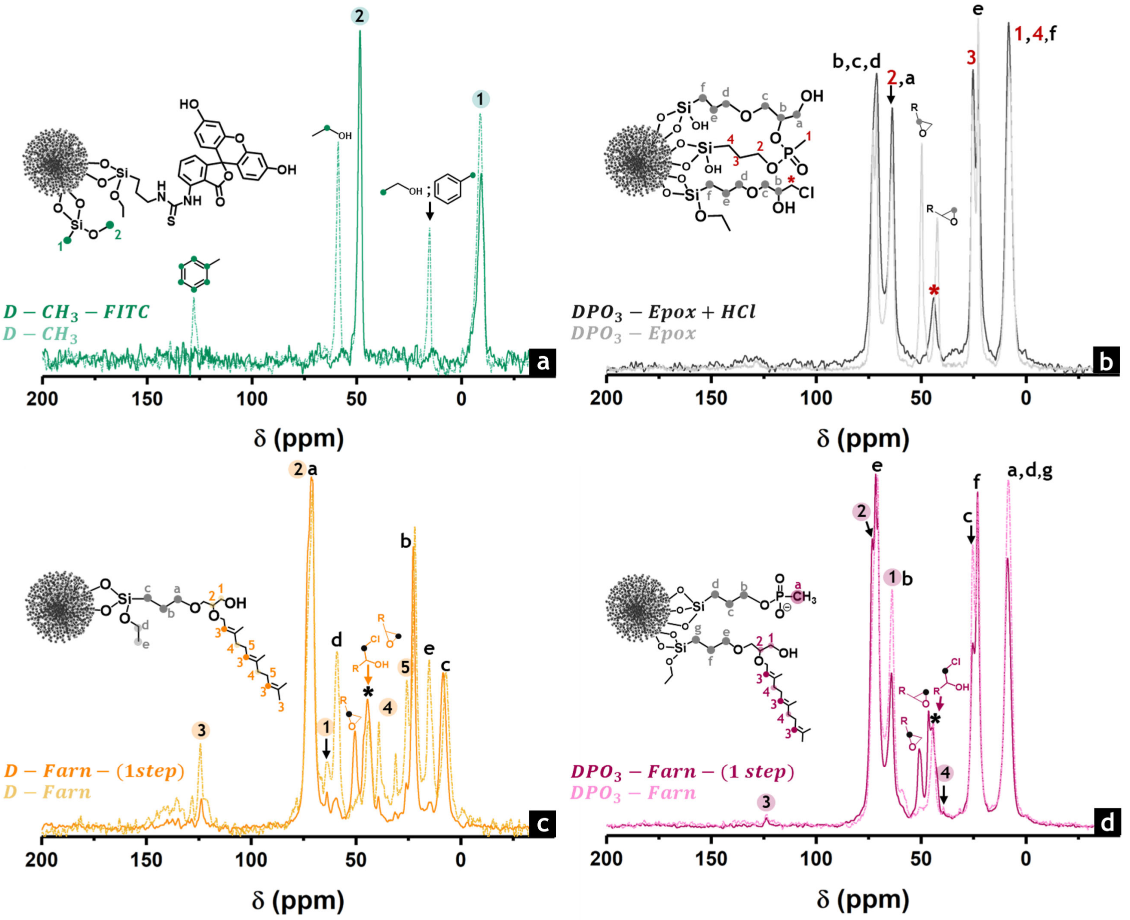

Figure S5. Solid-state ^{13}C CP-NMR spectra of (a) FITC-labeled **D-CH$_3$** and synthetic controls: (b) **DPO$_3$-Epox+HCl**, (c) **D-Farn-(1 step)**, and (d) **DPO$_3$-Farn-(1 step)**. The spectra of the respective equivalent samples (*i.e.*, **D-CH$_3$**, **DPO$_3$-Epox**, **D-Farn**, and **DPO$_3$-Farn**) were overlapped (light dotted lines) for comparison.

Table S3. Assignment of the signals of the solid-state ^{13}C CP-NMR spectra.

Sample	Signal[2,3]	Assignment, δ (ppm)
**D-PEG**	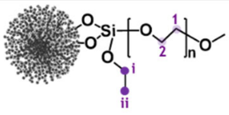	**1,2:** 70.1; **i:** 58.6; **ii:** 15.0
**D-PO$_3$**	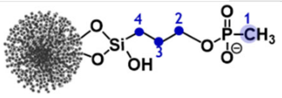	**1,4:** 8.1; **2:** 63.7; **3:** 25.4
**D-CH$_3$**	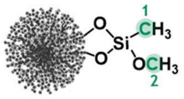	**1:** -8.7; **2:** 48.1;<br>*Toluene:* 15.3, 127.8;<br>*Ethanol:* 58.9, 15.3
**D-Farn**	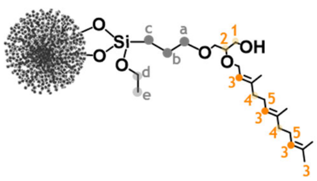	*Precursor (***D-Epox***):*<br>**a:** 71.0; **b:** 21.7; **c:** 7.2; **d:** 59.1; **e:** 15.5;<br>R-epoxide: 44.2, 50.9 (GPTMS);<br>**1:** 63.9; **2:** 71.6 (glycerol)<br>**3:** 123.9; **4:** 39.5; **5:** 26.0 (**Farn**)<br>*: 44.3(chlorohydrin)

**DPO$_3$-CH$_3$**	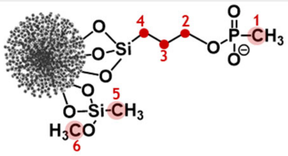	**1,4:** 8.1; **2:** 63.4; **3:** 25.4; **5:** -7.1; **6:** 48.2
**DPO$_3$-Farn**	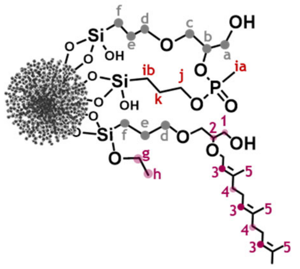	*Precursor (***DPO$_3$-Epox***):*<br>**a:** 64.4; **b, c, d:** 70.7; **e:** 22.7; **f:** 8.1;<br>**g:** 59.1; **h:** 14.1;<br>**i:** 8.1 (THMP); **j:** 64.4 (THMP);<br>**k:** 25.2 (THMP);<br>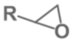: 42.4, 49.7 (GPTMS);<br>**1:** 64.4; **2:** 70.7 (glycerol)<br>**3:** 123.9; **4:** 39.2; **5:** 15.4<br>*: 44.1 (chlorohydrin)
**DPO$_3$-Epox + HCl**	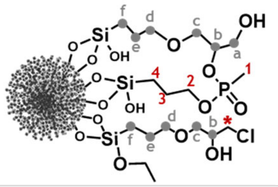	**a:** 64.0; **b, c, d:** 71.3; **e:** 22.3;<br>**f, 1, 4:** 8.3; **2:** 64.0; **3:** 25.3<br>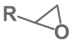: 42.2, 49.7 (Precursor: D_{PO_3-Epox});<br>*: 44.3 (chlorohydrin)
**D-Farn-(1 step)**	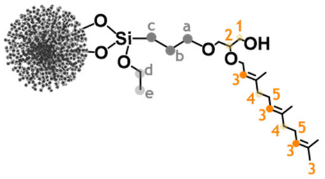	**a:** 71.6; **b:** 22.2; **c:** 8.4; **d:** 59.7; **e:** 14.7<br>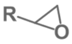: 44.2, 50.4 (GPTMS)<br>**1:** 63.9; **2:** 71.6 (glycerol)<br>**3:** 123.9; **4:** 39.5; **5:** 26.0 (**Farn**)<br>*: 44.2 (chlorohydrin)
**DPO$_3$-Farn-(1 step)**	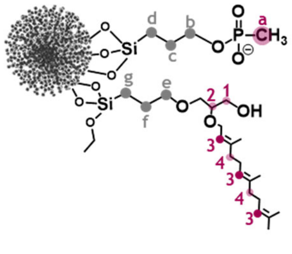	**a, d, g:** 8.7; **b:** 64.2; **c:** 25.7; **e:** 71.3;<br>**f:** 22.9;<br>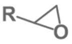: 46.5, 50.9 (GPTMS);<br>**1:** 63.7; **2:** 73.3 (glycerol)<br>**3:**123.9; **4:** 39.7 (**Farn**)<br>*: 44.5 (chlorohydrin)

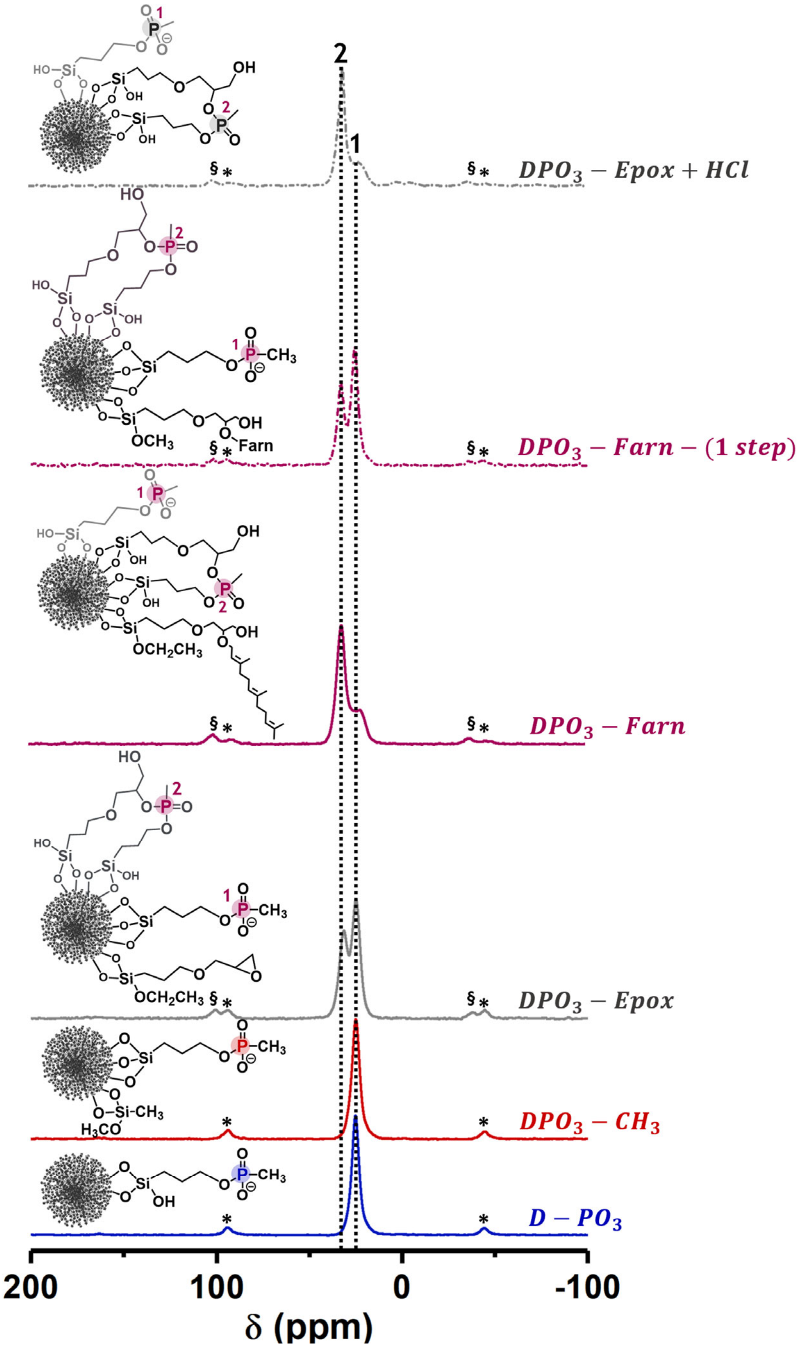

Figure S6. Solid-state ^{31}P MAS-NMR spectra of the silica nanoparticles functionalized with phosphonate moieties: **D-PO₃** (blue), **DPO₃-CH₃** (red), **DPO₃-Epox** (gray), and **DPO₃-Farn** (pink), as well as the synthetic controls **DPO₃-Farn-(1 step)** (pink dashed lines) and **DPO₃-Epox+HCl** (grey dashed lines). The spinning sidebands corresponding to the phosphonate species $ROP(O)CH_3O^{-}$) and the phosphorus of the cyclic THMP-GPTMS conjugate are marked with * and §, respectively.

Table S4. Assignment of the signals of the solid-state ^{31}P MAS-NMR spectra.

Sample	Signal[3]	δ (ppm)
**D-PO$_3$**	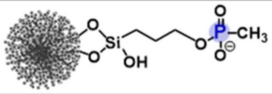	25.3
**DPO$_3$-CH$_3$**	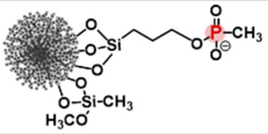	25.1
**DPO$_3$-Epox**	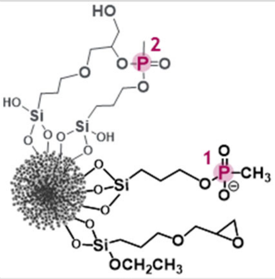	**1:** 25.2 *(majority product)*
		**2:** 31.3
**DPO$_3$-Farn**	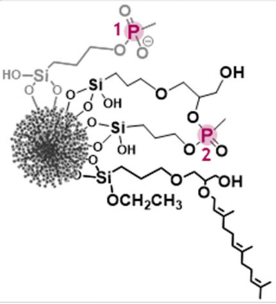	**1:** 21.3
		**2:** 31.4 *(majority product)*
**DPO$_3$-Farn-(1 step)**	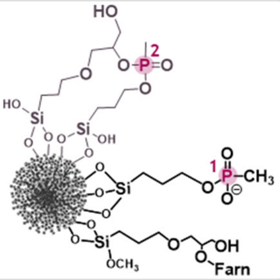	**1:** 25.7 *(majority product)*
		**2:** 32.9
**DPO$_3$-Epox + HCl**	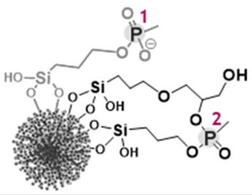	**1:** 22.5
		**2:** 31.1 *(majority product)*

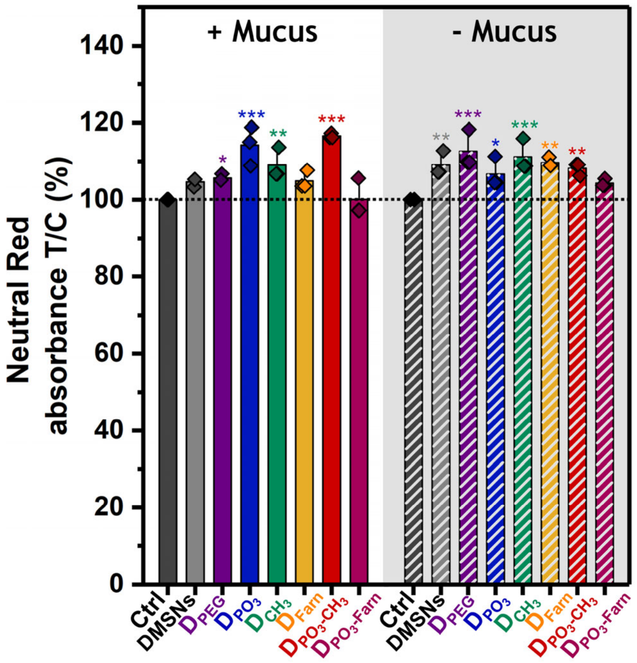

Figure S7. Cell viability of Caco-2/HT29-MTX-E12 co-culture measured by Neutral Red assay after 6 h of incubation with silica nanoparticles in the presence or absence of mucus. Results are presented as means + standard deviations (n=3), normalized to the control (*i.e.*, non-treated cells), and measured in three biological replicates performed in technical duplicate. Statistically significant differences, according to the Student's t-test, when treatments were compared to the respective controls with or without mucus, were expressed by * ($p < 0.05$), ** ($p < 0.01$), or *** ($p < 0.001$).

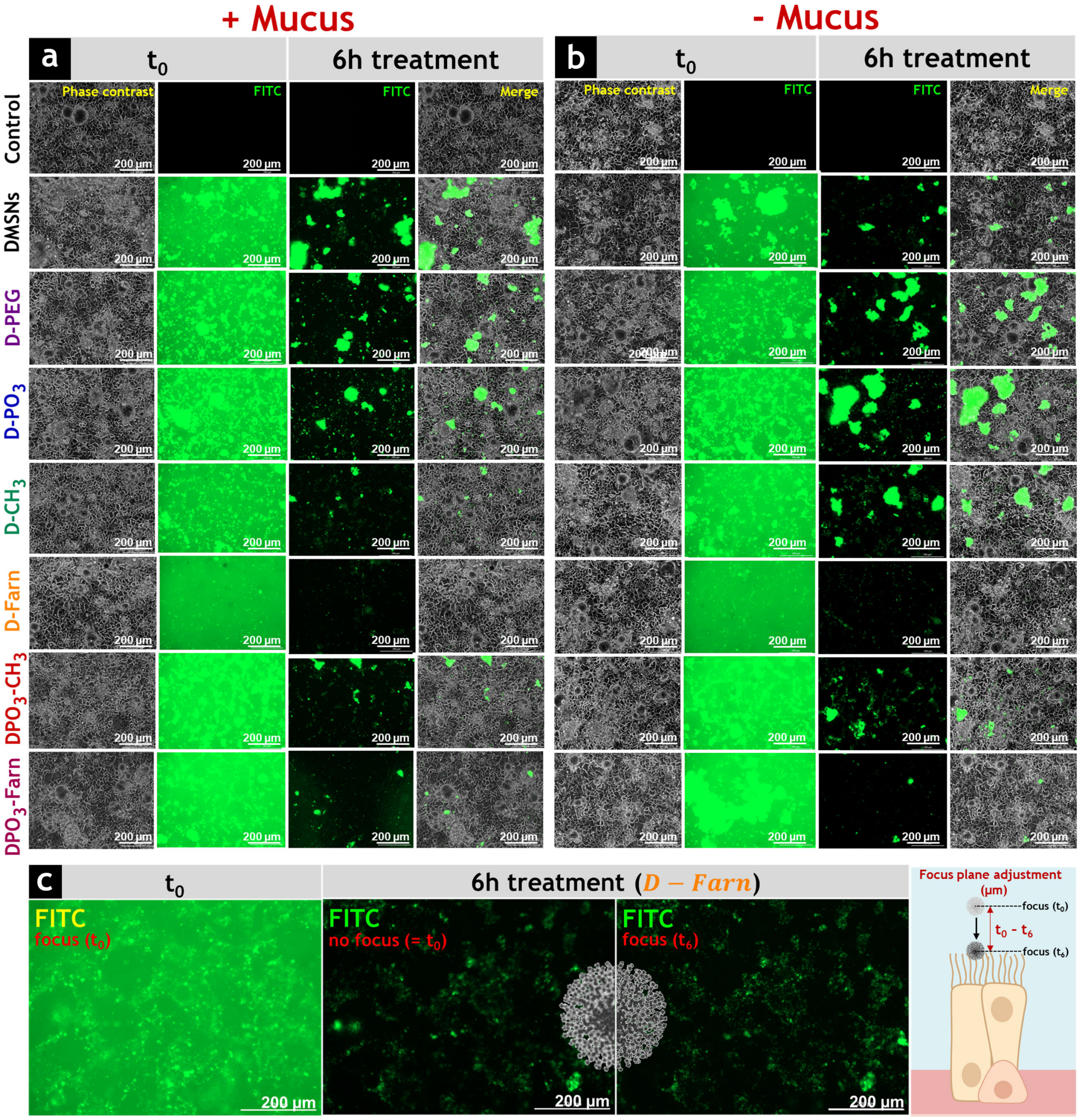

Figure S8. Representative phase contrast images (10× magnification) obtained immediately after nanoparticle treatments (t_0) and after 6 h-incubation at 37 °C (t_6). The images were acquired at the exact coordinates in both time points of the assay, and the particle-cell interactions were tested (a) in the presence or (b) absence of mucus. The control corresponds to non-treated cells incubated in the complete cell culture medium. (c) Representative phase contrast images (10× magnification) showing the change in the focus position (GFP [469, 525 nm] channel) detected after 6 h-incubation with FITC-labeled **D-Farn**. Scale bars stand for 200 µm. The schematic representation of the particle redistribution and the corresponding calculation of focal plane adjustment (*i.e.*, t_0-t_6) was created with BioRender.com.

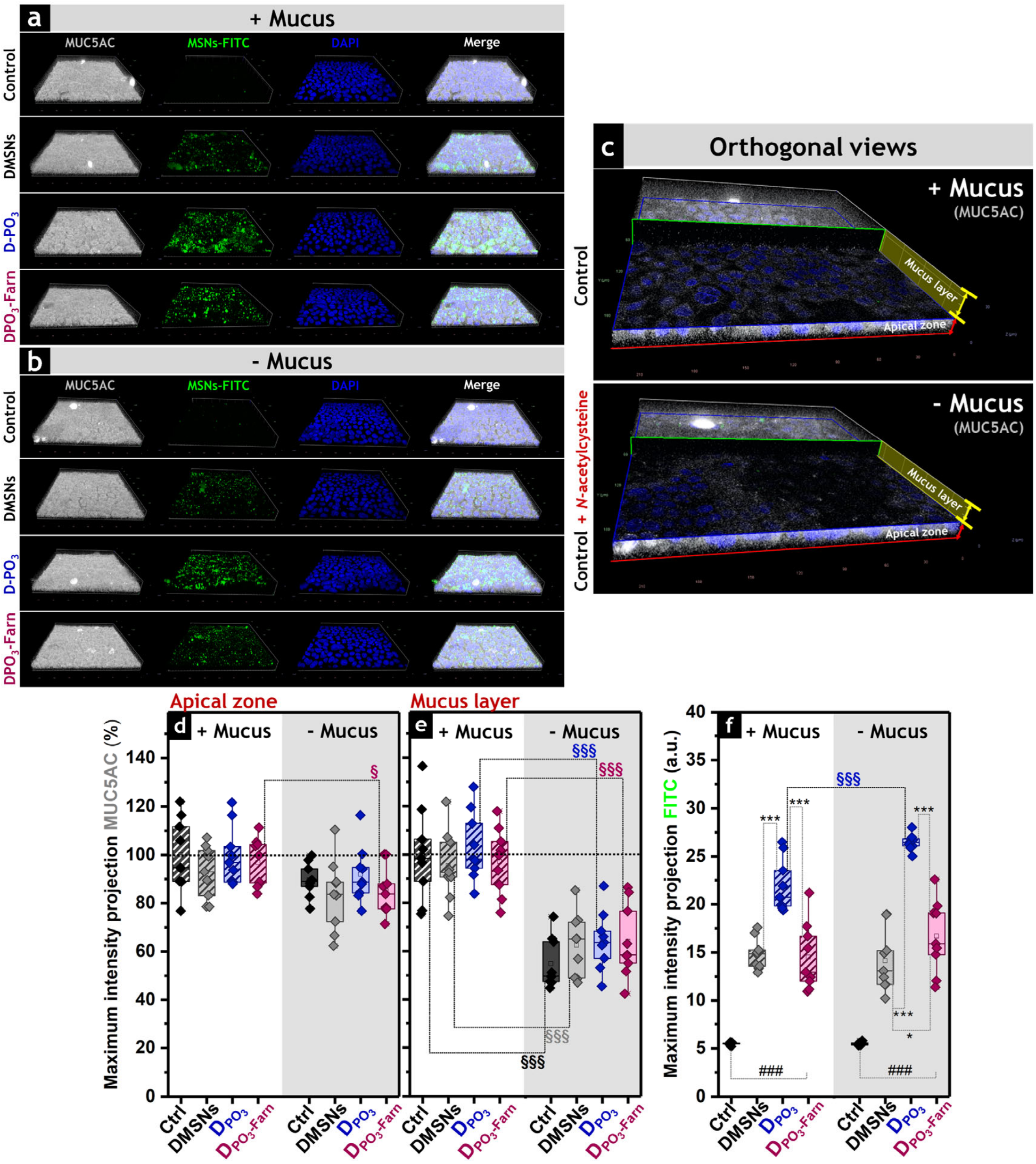

Figure S9. Representative immunofluorescence staining of MUC5AC (white) without (a) or with (b) previous treatment with *N*-acetylcysteine solution in serum-free medium (10 mM). The 3D reconstructions (63× magnification) were obtained by z-stack imaging after 6 h-treatment with FITC-labeled silica nanoparticles (green). The scale bar segmentation is 10 µm, and the nuclei are represented in blue (DAPI staining). (c) Representative orthogonal views of the control (*i.e.*, non-treated cells) where the MUC5AC staining was detected up to the apical zone of the cells and on top of the co-culture without (top) or with (bottom) pre-incubation with *N*-acetylcysteine solution (10 mM). Quantification of the maximum intensity projection of MUC5AC (%) (d) up to the apical zone and (e) on top of the cell monolayer (n = 9 optical fields). (f) Quantification of the mean fluorescence intensity of FITC obtained from the maximum intensity projections (n = 9 optical fields). Statistically significant differences according to one-way ANOVA and Fisher Tests when treatments are compared in the presence or absence of mucus (*), when the effect of mucus is compared for the same treatment (§), or when a specific treatment is compared with a group of others (#), are indicated with */§/# ($p < 0.05$), **/§§/## ($p < 0.01$), or ***/§§§/### ($p < 0.001$).

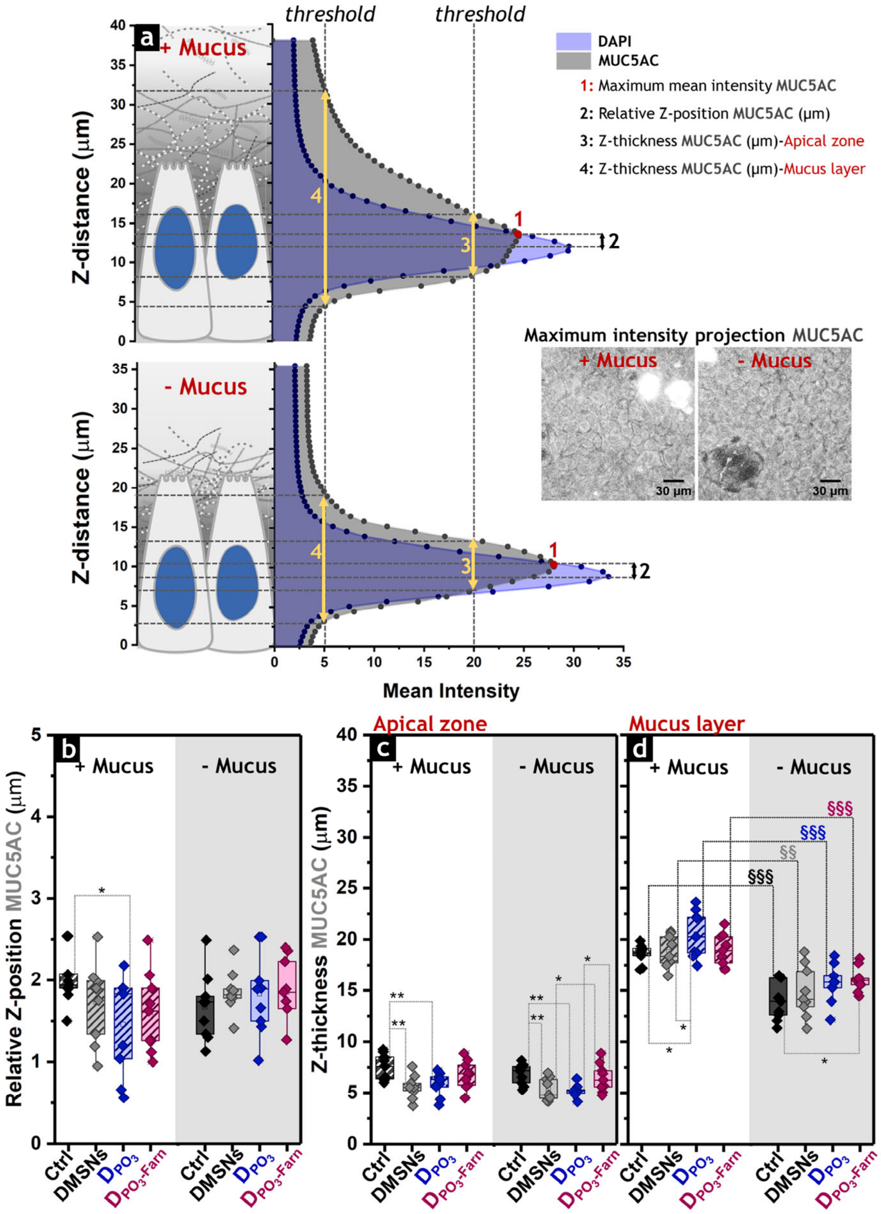

Figure S10. (a) Representative Z-plot profiles of MUC5AC (grey) and DAPI stainings (blue) obtained from 3D reconstructions of the Control (*i.e.*, non-treated cells) without (top) or with (bottom) pre-incubation with *N*-acetylcysteine solution in serum-free medium (10 mM) prior 6 h-treatment with silica particles. Schematic representations of the intestinal cells and mucus layer were created with BioRender.com. The insert shows representative Z-projection images of MUC5AC in the different conditions tested (± Mucus), whose scale bars stand for 30 µm. (b) Quantification of the relative Z-position (µm) of the MUC5AC staining after 6 h-incubation with FITC-labeled nanoparticles (*i.e.*, **DMSNs**, **D-PO$_3$**, and **DPO$_3$-Farn**) with or without mucus (n = 9 optical fields). (c) Quantification of the Z-thickness (µm) of the MUC5AC staining in the apical zone after 6 h-incubation in the presence of FITC-labeled nanoparticles, including or not previous treatment with *N*-acetylcysteine (n = 9 optical fields). (d) Quantification of the total Z-thickness (µm) of the MUC5AC. Statistically significant differences according to one-way ANOVA and Fisher Tests when treatments are compared in the presence or absence of mucus (*) or when the effect of mucus is compared for the same treatment (§), are indicated with */§ ($p < 0.05$), **/§§ ($p < 0.01$), or ***/§§§ ($p < 0.001$).

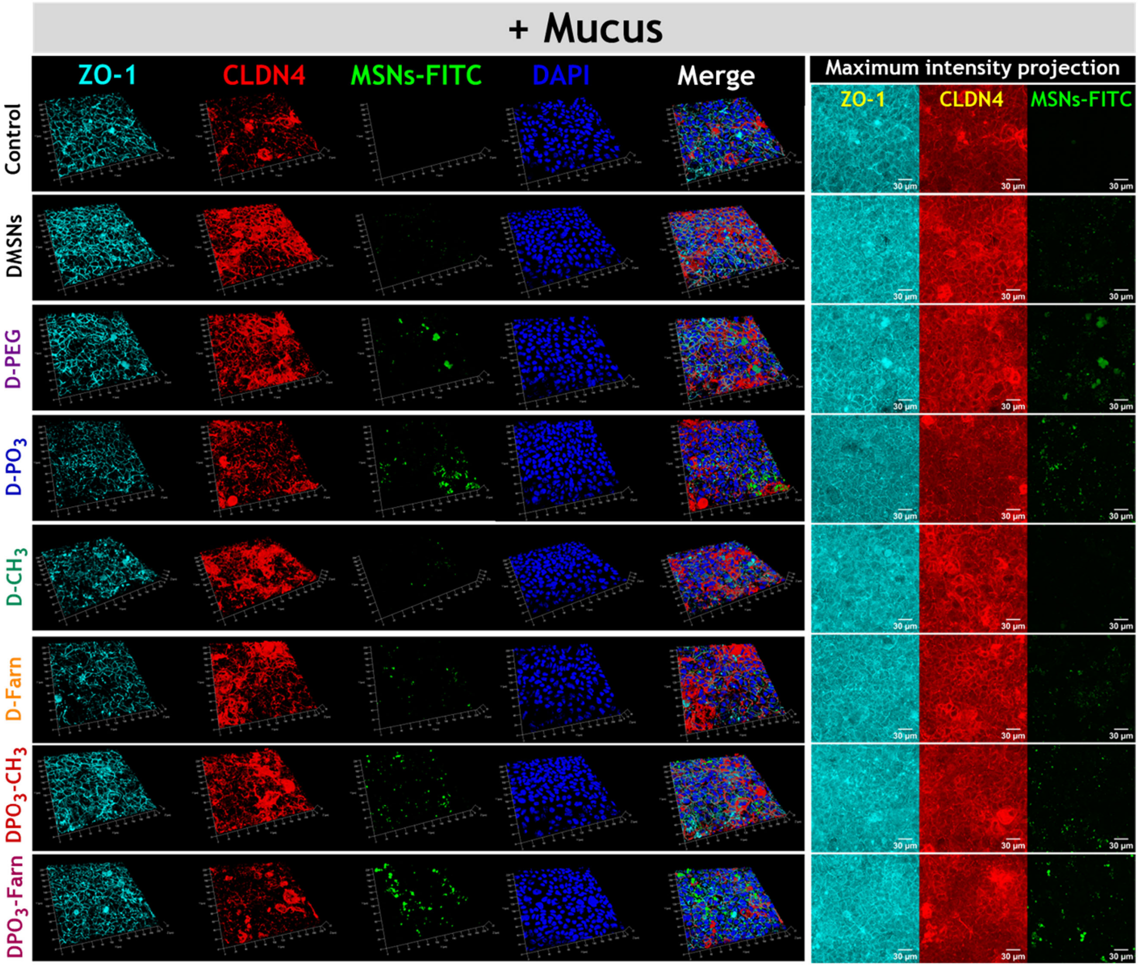

Figure S11. Representative immunofluorescence staining of ZO-1 (cyan) and CLDN4 (red). The 3D reconstructions (63× magnification) were obtained by z-stack imaging after 6 h-treatment with FITC-labeled silica nanoparticles (green) in the presence of mucus. The control corresponds to non-treated cells incubated in the complete cell culture medium. The scale bar segmentation is 10 µm, and the nuclei are represented in blue (DAPI staining). Scale bars of the Z-projection images of ZO-1, CLDN4, and FITC channels for each 3D reconstruction stand for 30 µm.

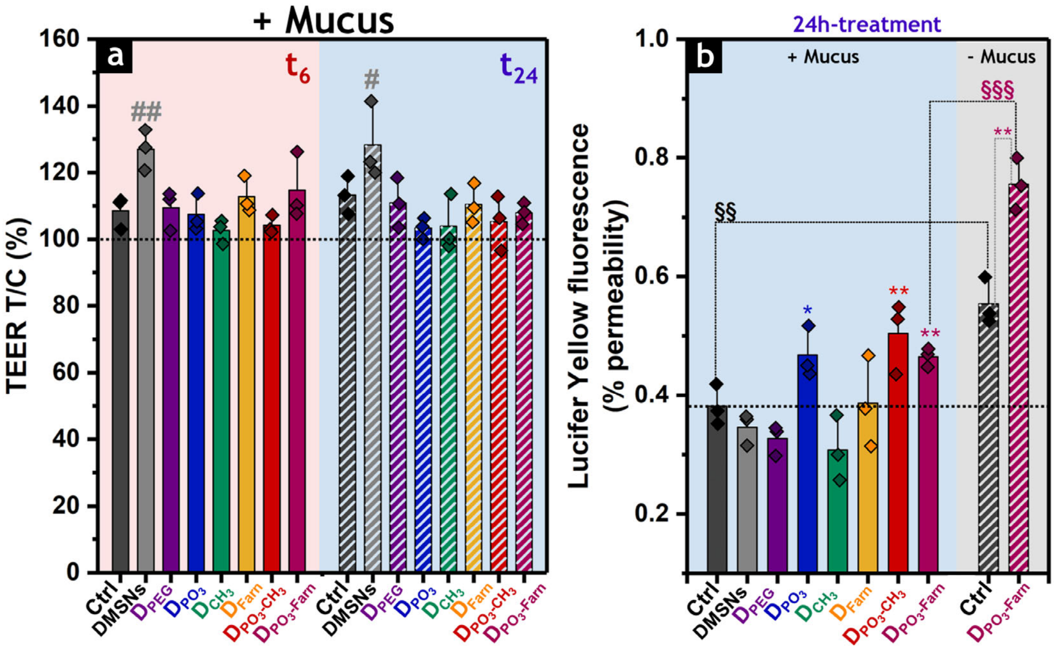

Figure S12. (a) Transepithelial electrical resistance (TEER) measured after 6 h and 24 h of incubation of Caco-2/HT29-MTX cells with non-fluorescent silica nanoparticles (*i.e.*, **DMSNs**, **D-PEG**, **D-PO3**, **D-CH3**, **D-Farn**, **DPO3-CH3**, and **DPO3-Farn**) or cell culture medium (control, *i.e.*, non-treated cells). The TEER (%) values were calculated as a percentage of the TEER before the addition of the particles (dashed line). The treatment with calcined **DMSNs** was significantly different (#) from the treatments with functionalized particles and the respective control after incubations for 6 h (##, $p < 0.01$) and 24 h (#, $p < 0.05$) according to the Student's *t*-test. (b) Lucifer Yellow fluorescence intensity in the basolateral compartment after incubation for 24 h with **DMSNs**, **D-PEG**, **D-PO3**, **D-CH3**, **D-Farn**, **DPO3-CH3**, and **DPO3-Farn** in standard conditions (+ Mucus), as well after mucus removal and subsequent cell treatment with **DPO3-Farn** for 24 h (– Mucus). The dashed line represents the % permeability corresponding to the control (+ Mucus). Statistically significant differences according to Student's *t*-test when treatments were compared to the respective controls (*i.e.*, non-treated cells) were indicated with * ($p < 0.05$) or ** ($p < 0.01$). Statistically significant differences when the effect of mucus is compared (§) were indicated with §§ ($p < 0.01$) or §§§ ($p < 0.001$). All data were obtained from three independent cell preparations (biological triplicates) and presented as mean ± standard deviations (n=3).

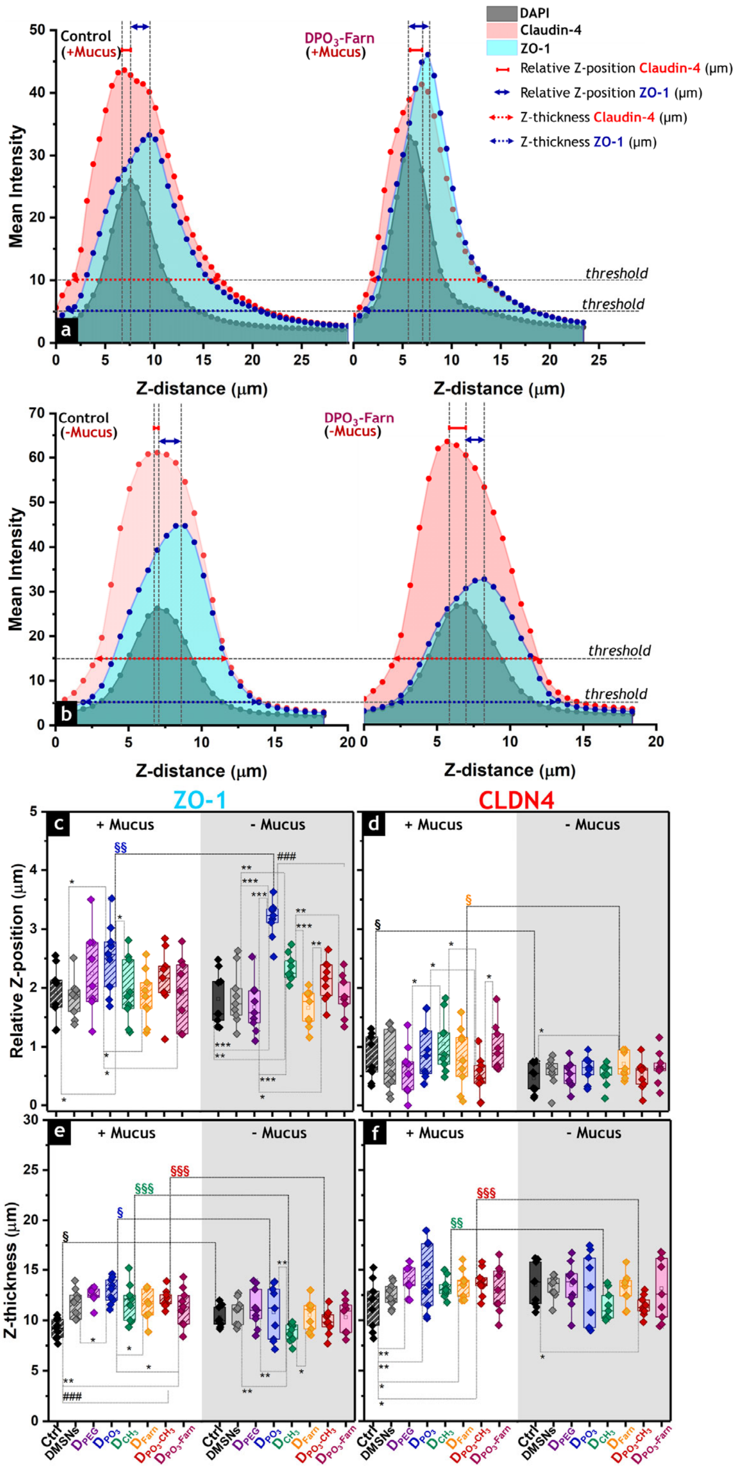

Figure S13. Representative Z-plot profiles of ZO-1 (blue), CLDN4 (red), and DAPI stainings (grey) obtained from 3D reconstructions of Control (*i.e.*, non-treated cells) and **DPO_3-Farn** treatment (a) in the presence or (b) absence of mucus. (c, d) Quantification of the relative Z-position (μm) and (e, f) Z-thickness (μm) of (c, e) ZO-1 and (d, f) CLDN4 distributions. All data were obtained from three biological replicates (n = 9 optical fields). Statistically significant differences according to one-way ANOVA and Fisher Tests when treatments are compared in the presence or absence of mucus (*), when the effect of mucus is compared for the same treatment (§), or when a specific treatment is compared with a group of others (#), are indicated with */§/# ($p < 0.05$), **/§§/## ($p <0.01$), or ***/§§§/### ($p < 0.001$).

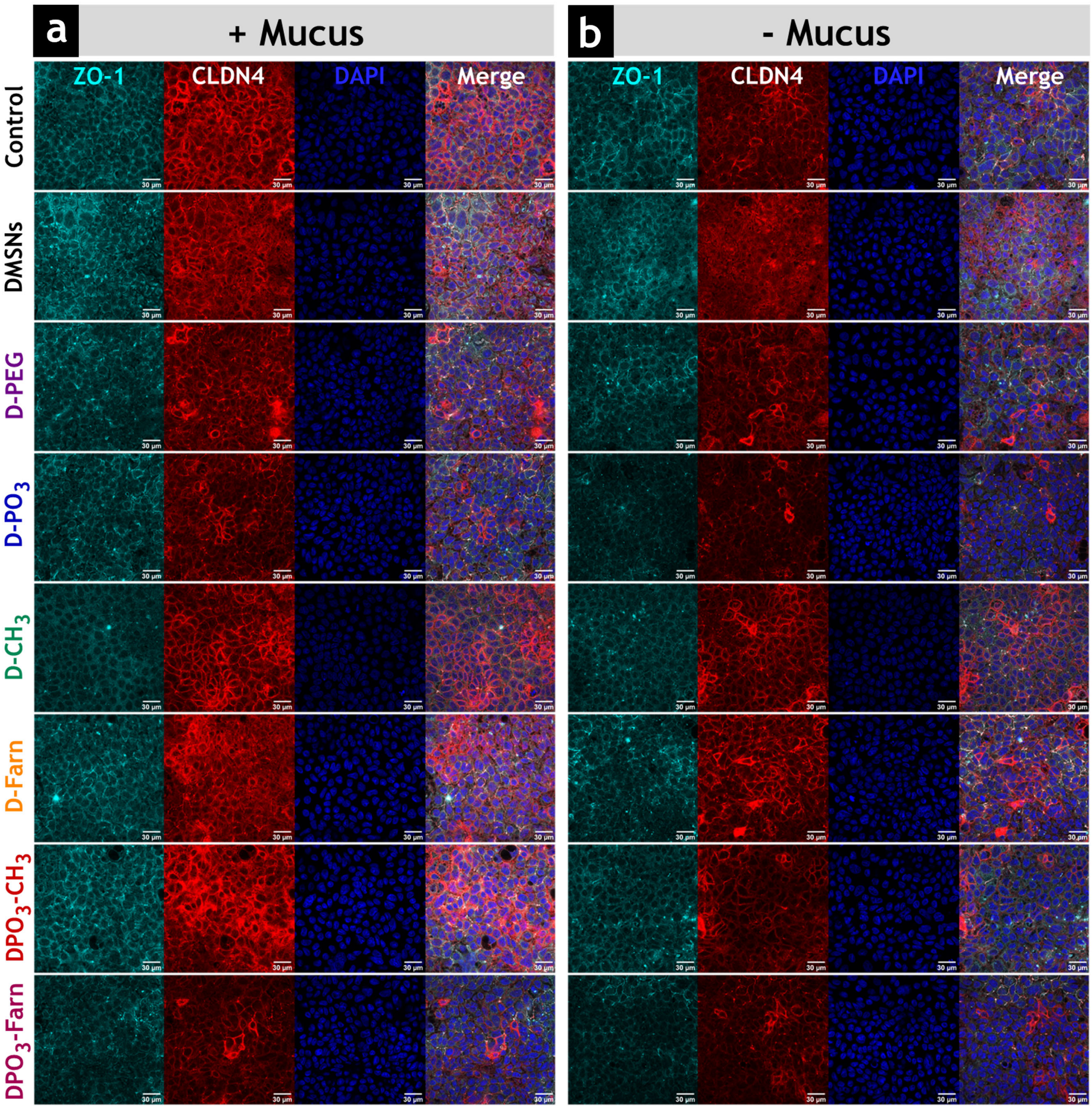

Figure S14. Immunofluorescence staining of tight junction proteins performed after 6 h of treatment of Caco-2/HT29-MTX-E12 cells with silica nanoparticles in the presence or absence of mucus. The control corresponds to non-treated cells incubated in the complete cell culture medium. ZO-1 staining is represented in cyan, CLDN4 in red, and the nuclei (stained with DAPI) in blue. The scale bars stand for 30 µm.

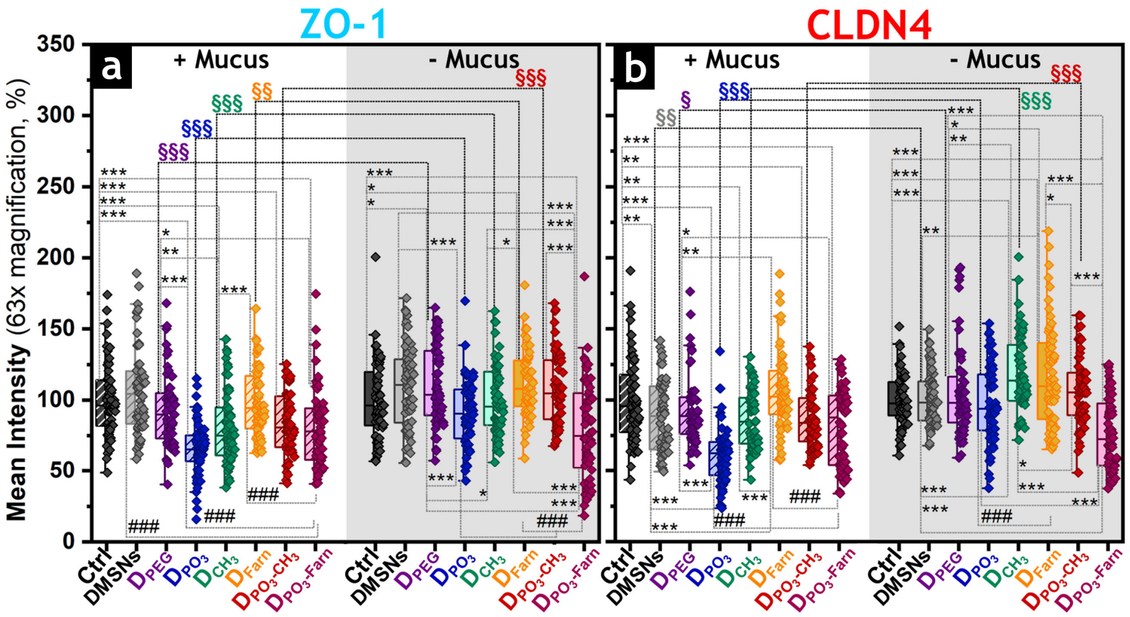

Figure S15. Quantification of the mean fluorescence intensities of (a) ZO-1 and (b) CLDN4 after 6 h-treatment with FITC-labeled silica nanoparticles. The control corresponds to non-treated cells incubated in the complete cell culture medium. Each dataset resulted from the analysis of $n \geq 60$ cells from three independent preparations (biological triplicates). Statistically significant differences according to one-way ANOVA and Fisher Tests when treatments are compared in the presence or absence of mucus (*), when the effect of mucus is compared for the same treatment (§), or when a specific treatment is compared with a group of others (#), are indicated with */§/# ($p < 0.05$), **/§§/## ($p < 0.01$), or ***/§§§/### ($p < 0.001$).

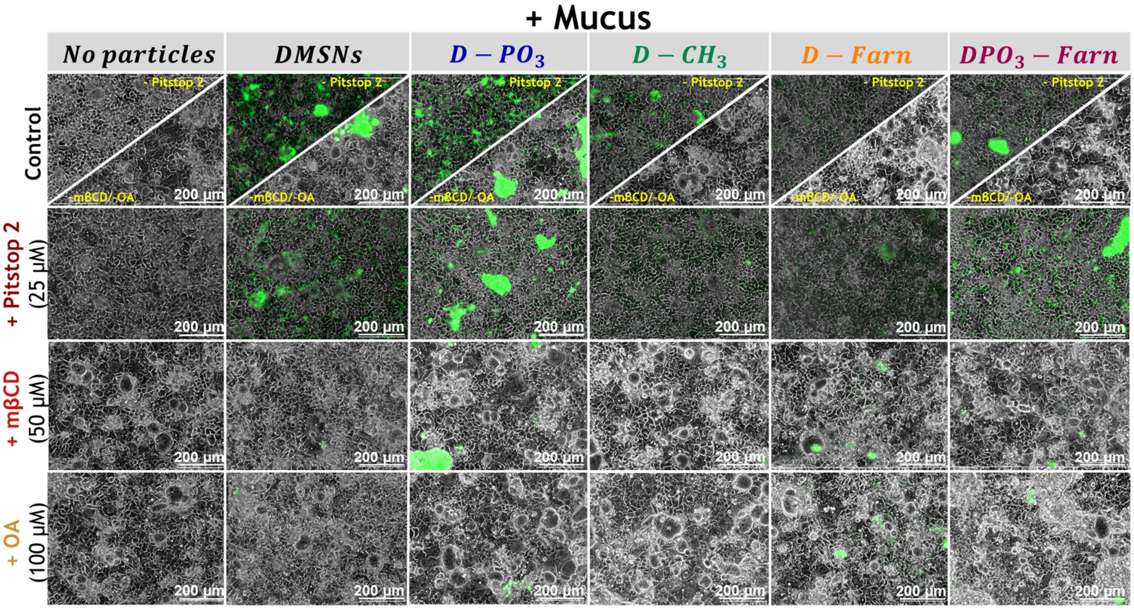

Figure S16. (a) Appearance of Caco-2/HT29-MTX-E12 cells after pre-incubation with Pitstop 2 (25 µM, 0.3% DMSO), mβCD (50 µM, 0.05% DMSO), and OA (100 µM, 0.03% DMSO) followed by 6 h-treatment with FITC-labeled particles in the presence of mucus (10× magnification). The representative phase contrast images of the negative controls correspond to cells pre-incubated with serum-free medium (– Pitstop **2**, 0.3% DMSO, 10 min) or in the presence of BSA (– mβCD/– OA, 0.05% DMSO, 20 h) and subsequently treated with particles. Scale bars stand for 200 µm.

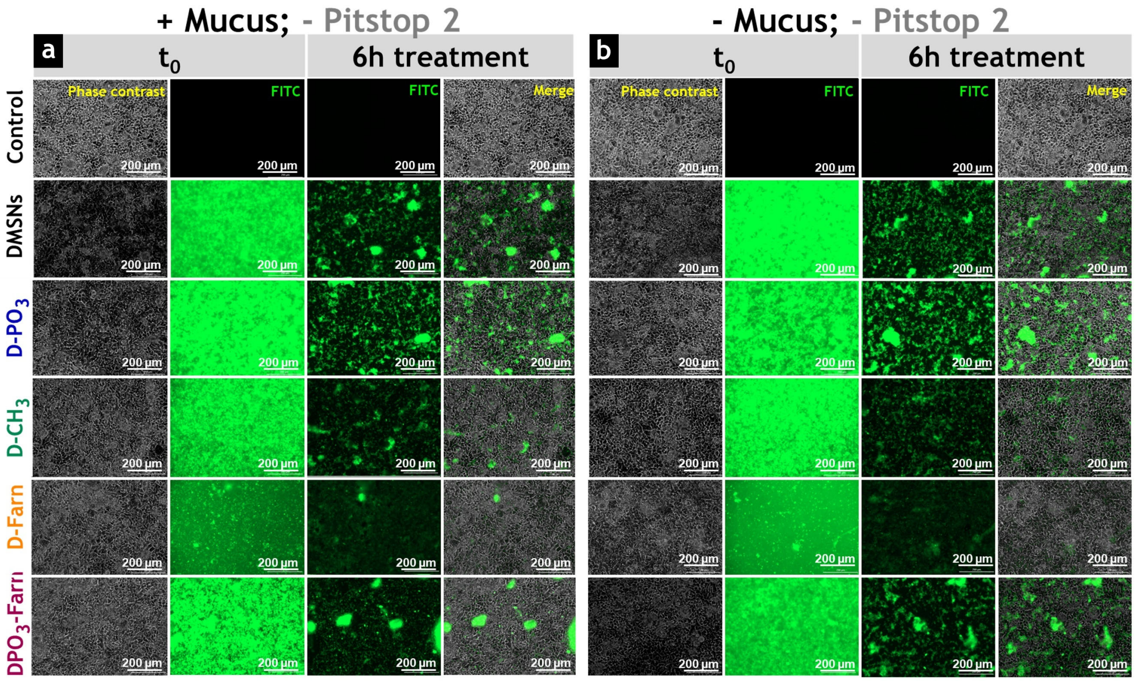

Figure S17. Representative phase contrast images (10× magnification) obtained after pre-incubation with serum-free medium (+ 0.3% DMSO, 10 min) followed by the application of serum-free medium (Control) or FITC-labeled nanoparticles (*i.e.*, **DMSNs**, **D-PO$_3$**, **D-CH$_3$**, **D-Farn**, and **DPO$_3$-Farn**). Live cell imaging was performed (a) in the presence or (b) absence of mucus. The images were acquired immediately after silica nanoparticle treatments (t_0) and after 6 h-incubation at 37 °C (t_6) in a serum-free medium at the exact coordinates in both time points of the assay. The change in the focus position and mean intensity corresponding to the FITC-labeled particles (GFP [469, 525 nm] channel) was detected after 6 h-incubation. Scale bars stand for 200 µm.

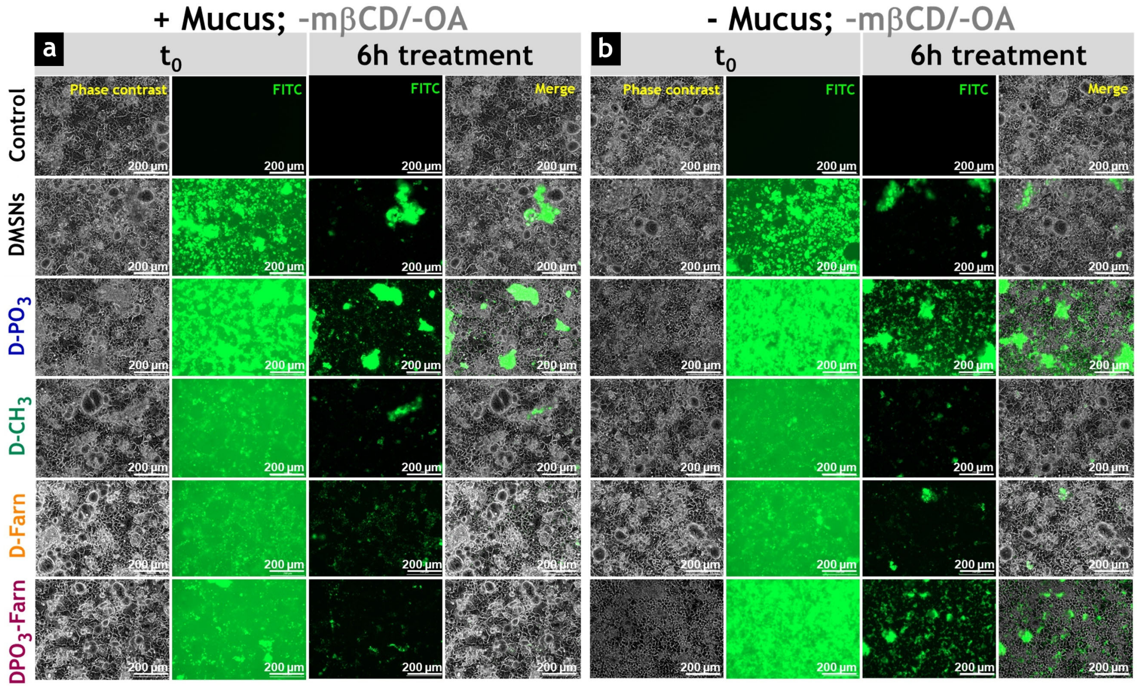

Figure S18. Representative phase contrast images (10× magnification) obtained after pre-incubation with serum-free medium containing BSA (1 mg·mL^{-1}, + 0.05% DMSO, 20 h) followed by the application of serum-free medium (Control) or FITC-labeled nanoparticles (*i.e.*, **DMSNs**, **D-PO$_3$**, **D-CH$_3$**, **D-Farn**, and **DPO$_3$-Farn**). Live cell imaging was performed (a) in the presence or (b) absence of mucus. The images were acquired immediately after silica nanoparticle treatments (t_0) and after 6 h-incubation at 37 °C (t_6) in a serum-free medium at the exact coordinates in both time points of the assay. The change in the focus position and mean intensity corresponding to the FITC-labeled particles (GFP [469, 525 nm] channel) was detected after 6 h-incubation. Scale bars stand for 200 µm.

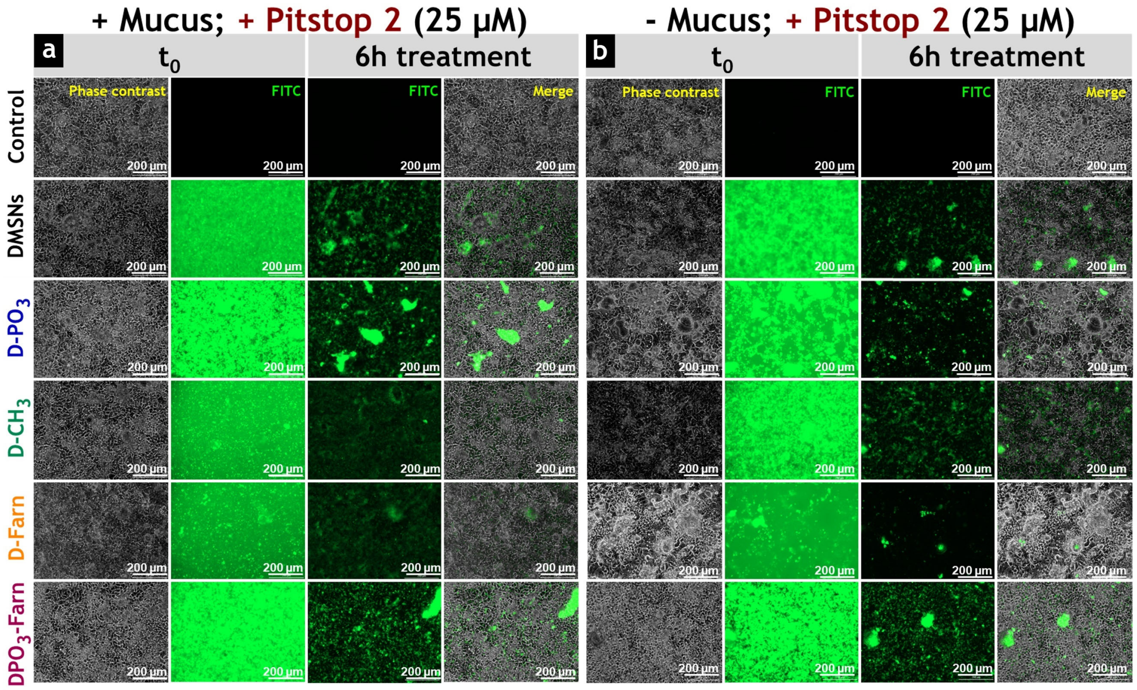

Figure S19. Representative phase contrast images (10× magnification) obtained after treatment with Pitstop 2 (25 µM, 0.3% DMSO, 10 min) and subsequent incubation with serum-free medium (Control) or with FITC-labeled nanoparticles (*i.e.*, **DMSNs**, **D-PO**$_3$, **D-CH**$_3$, **D-Farn**, and **DPO**$_3$**-Farn**). Live cell imaging was performed (a) in the presence or (b) absence of mucus. The images were acquired immediately after silica nanoparticle treatments (t_0) and after 6 h-incubation at 37 °C (t_6) in a serum-free medium at the exact coordinates in both time points of the assay. The change in the focus position and mean intensity corresponding to the FITC-labeled particles (GFP [469, 525 nm] channel) was detected after 6 h-incubation. Scale bars stand for 200 µm.

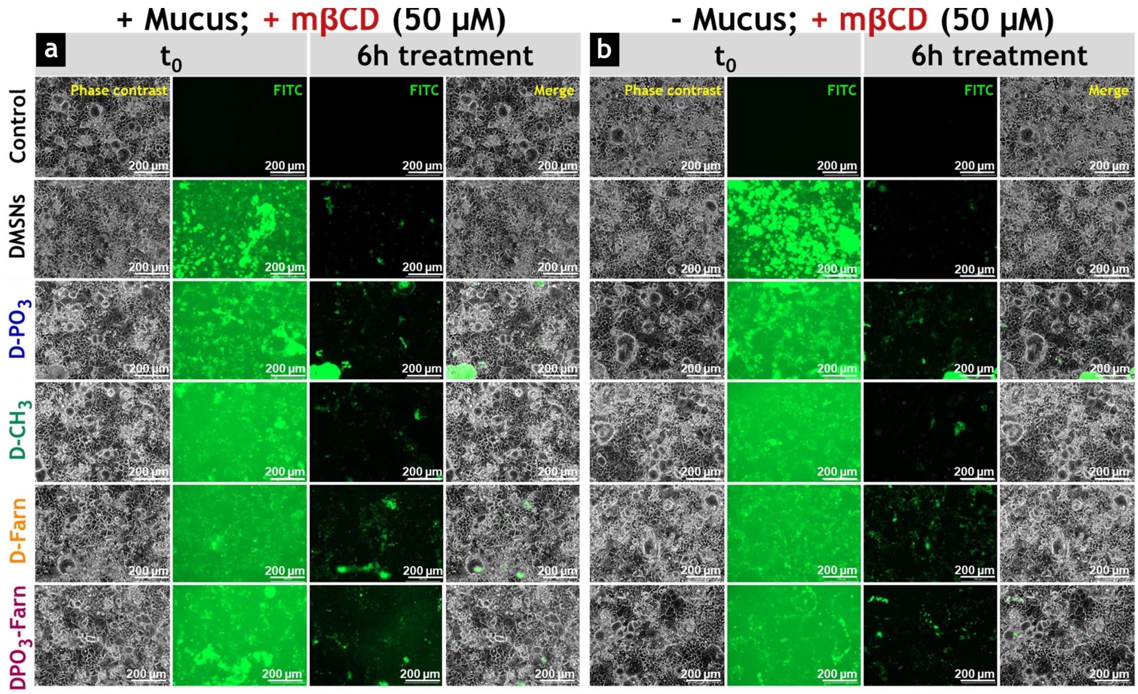

Figure S20. Representative phase contrast images (10× magnification) obtained after treatment with mβCD (50 µM, 0.05% DMSO, 20 h) and subsequent incubation with serum-free medium (Control) or with FITC-labeled nanoparticles (*i.e.*, **DMSNs**, **D-PO3**, **D-CH3**, **D-Farn**, and **DPO3-Farn**). Live cell imaging was performed (a) in the presence or (b) absence of mucus. The images were acquired immediately after silica nanoparticle treatments (t_0) and after 6 h-incubation at 37 °C (t_6) in serum-free medium at the exact coordinates in both time points of the assay. The change in the focus position and mean intensity corresponding to the FITC-labeled particles (GFP [469, 525 nm] channel) was detected after 6 h-incubation. Scale bars stand for 200 µm.

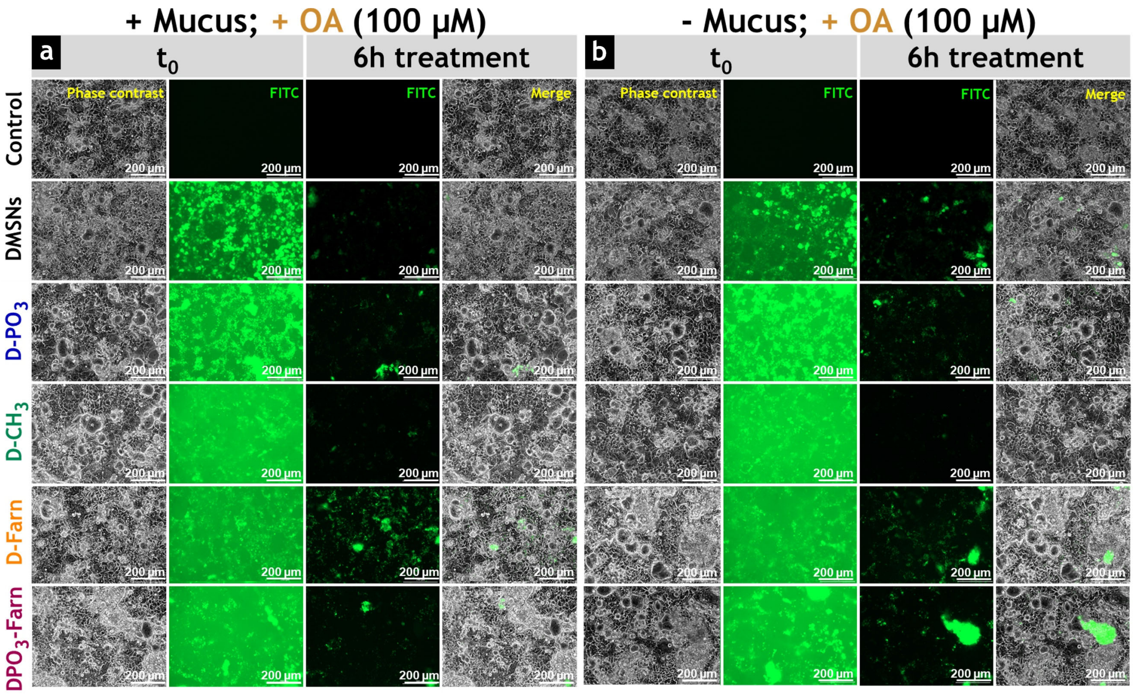

Figure S21. Representative phase contrast images (10× magnification) obtained after treatment with OA (100 µM, 0.03% DMSO, 20 h) and subsequent incubation with serum-free medium (Control) or with FITC-labeled nanoparticles (*i.e.*, **DMSNs**, **D-PO$_3$**, **D-CH$_3$**, **D-Farn**, and **DPO$_3$-Farn**). Live cell imaging was performed (a) in the presence or (b) absence of mucus. The images were acquired immediately after silica nanoparticle treatments (t_0) and after 6 h-incubation at 37 °C (t_6) in serum-free medium at the exact coordinates in both time points of the assay. The change in the focus position and mean intensity corresponding to the FITC-labeled particles (GFP [469, 525 nm] channel) was detected after 6 h-incubation. Scale bars stand for 200 µm.

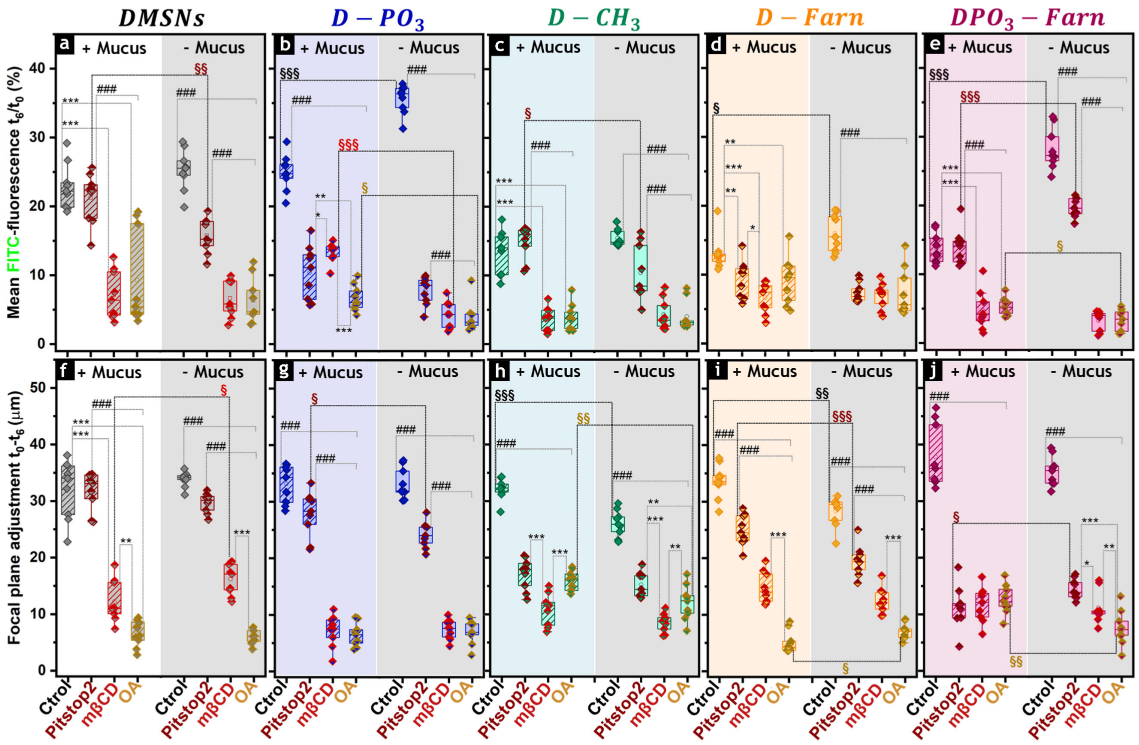

Figure S22. Extended comparison among particle treatments in the presence of different chemical modulators. (a-e) Quantification of the residual FITC fluorescence due to particle–cell interactions (%) with and without the mucus layer when the cells were pre-incubated in control conditions (– mβCD/ – OA) or in the presence of Pitstop 2, mβCD, or OA followed by 6 h-treatment with (a) **DMSNs**, (b) **D-PO₃**, (c) **D-CH₃**, (d) **D-Farn**, or (e) **D-PO₃-Farn**. (f-j) Quantification of the focal plane adjustment obtained from the difference between the optical parameters set immediately after FITC-labeled nanoparticles treatment (t_0) and after 6 h-incubation (t_6) of cells with (f) **DMSNs**, (g) **D-PO₃**, (h) **D-CH₃**, (i) **D-Farn**, or (j) **D-PO₃-Farn**. In the negative controls (– mβCD/ – OA), the cells were pre-incubated in serum-free medium containing BSA (1 mg·mL^{-1}, + 0.05% DMSO, 20 h) and then were treated with FITC-labeled nanoparticles dispersed in serum-free medium. Experiments were performed in biological triplicates, and at least 9 paired images were analyzed before and after focus adjustment (n = 9). Statistically significant differences according to one-way ANOVA and Fisher Tests when treatments are compared in the presence or absence of mucus (*), when the effect of mucus is compared for the same treatment (§), or when a specific treatment is compared with a group of others (#), are indicated with */§/# (p <0.5), **/§§/## (p <0.01), or ***/§§§/### (p < 0.001).

REFERENCES

[1] E. Juère, R. Caillard, D. Marko, G. Del Favero, F. Kleitz, *Chem. - A Eur. J.* **2020**, *26*, 5195.

[2] C. von Baeckmann, H. Kählig, M. Lindén, F. Kleitz, *J. Colloid Interface Sci.* **2021**, *589*, 453.

[3] M. Bouchoucha, M. F. Côté, R. C.-Gaudreault, M. A. Fortin, F. Kleitz, *Chem. Mater.* **2016**, *28*, 4243.

[4] B. R. Moser, S. C. Cermak, K. M. Doll, J. A. Kenar, B. K. Sharma, *JAOCS, J. Am. Oil Chem. Soc.* **2022**, *99*, 801.

[5] L. Doan, H. Yagi, D. M. Jerina, D. L. Whalen, *J. Org. Chem.* **2004**, *69*, 8012.

[6] M. Hohagen, P. Guggenberger, E. Kiss, H. Kählig, D. Marko, G. Del Favero, F. Kleitz, *J. Colloid Interface Sci.* **2022**, *623*, 962.

[7] C. Iriarte-Mesa, M. Jobst, J. Bergen, E. Kiss, R. Ryoo, J. C. Kim, F. Crudo, D. Marko, F. Kleitz, G. Del Favero, G. Del Favero, *Nano Lett.* **2023**, *23*, 7758−7766.

[8] I. Behrens, A. I. Vila Pena, M. J. Alonso, T. Kissel, *Pharm. Res.* **2002**, *19*, 1185.

[9] J. Beisl, G. Pahlke, H. Abeln, M. Ehling-Schulz, G. Del Favero, E. Varga, B. Warth, M. Sulyok, W. Abia, C. N. Ezekiel, D. Marko, *Arch. Toxicol.* **2020**, *94*, 833.

[10] J. Beisl, G. Pahlke, M. Ehling-Schulz, G. Del Favero, D. Marko, *Toxins (Basel).* **2022**, *14*, 151.

[11] G. Repetto, A. del Peso, J. L. Zurita, *Nat. Protoc.* **2008**, *3*, 1125.

[12] J. Groestlinger, V. Spindler, G. Pahlke, M. Rychlik, G. Del Favero, D. Marko, *Chem. Res. Toxicol.* **2022**, *35*, 731.

[13] J. Beisl, E. Varga, D. Braun, B. Warth, M. Ehling-Schulz, G. Del Favero, D. Marko, *Toxins (Basel).* **2021**, *13*, 189.

[14] J. Groestlinger, C. Seidl, E. Varga, G. Del Favero, D. Marko, *Front. Nutr.* **2022**, *9*, 882222.

[15] S. Brunauer, P. H. Emmett, E. Teller, *J. Am.Chem.Soc.* **1938**, *60*, 309.

[16] R. Guillet-Nicolas, F. Bérubé, M. Thommes, M. T. Janicke, F. Kleitz, *J. Phys. Chem. C* **2017**, *121*, 24505.

[17] I. S. Protsak, Y. M. Morozov, W. Dong, Z. Le, D. Zhang, I. M. Henderson, *Nanoscale Res. Lett.* **2019**, *14*, 160.

[18] J. Cui, P. Chatterjee, I. I. Slowing, T. Kobayashi, *Microporous Mesoporous Mater.* **2022**, *339*, 112019.
